# Supplementary material for: Uncovering a miltiradiene biosynthetic gene cluster in the Lamiaceae reveals a dynamic evolutionary trajectory
Source: Nat Commun. 2023 Jan 20;14:343. doi: 10.1038/s41467-023-35845-1 (PMC9860074; doi:10.1038/s41467-023-35845-1)
Supplement: Supplementary file 1 — Supplementary Information [file 41467_2023_35845_MOESM1_ESM.pdf]

Uncovering a miltiradiene biosynthetic gene cluster in the Lamiaceae reveals a dynamic evolutionary trajectory

Bryson and Lanier *et al.*

Supplementary Table 1. A collection of Lamiaceae genomes published to date. (*genome assembly used in this study)

Species	Publication	Estimated genome size (Mb)	Assembly coverage (%)	BUSCO (% complete)	N50 contig	N50 scaffold
Callicarpa americana	*, [1]	538	94.0%	93.8%	7.5 Mb	29 Mb
Hyssopus officinalis	*, [2]	341	--	91.4%	n.r.	29 Mb
Lavandula angustifolia	[3]	870	79.1%	89.7%	n.r.	96 Kb
	[4]	1016	90.0%	91.4%	1.2 Mb	36 Mb
Mentha longifolia	[5]	400	88.0%	71.0%	n.r.	4 Kb
	[6]	469	--	91.5%	517 Kb	37 Mb
Nepta cataria	[2]	493	--	91.4%	n.r.	296 Kb
Nepta mussini	[2]	232	--	90.2%	n.r.	503 Kb
Ocimum basilicum	[7]	2360	87.6%	86.7%	48 Kb	1.5 Mb
Ocimum sanctum	[8]	386	--	n.r.	12 Kb	303 Kb
Ocimum tenuiflorum	[9]	612	61.0%	n.r.	n.r.	27.1 Kb
Origanum majorana	[7]	858	88.8%	89.5%	35 Kb	1.3 Mb
Origanum vulgare	[7]	705	89.4%	90.1%	26 Kb	157 Kb
Perilla citriodora	[10]	--	--	92.6%	68 Kb	1.2 Mb
Perilla frutescens	[10]	1379	97.5%	92.7%	3.21 Mb	62.6 Mb
Pogostemon cablin	*, [11]	2080	91.8%	90.6%	34 Kb	699 Kb
	[12]	1576	73.0%	n.r.	416 bp	1 Kb
	[13]	1040	186.5%	98.7%	7.8 Mb	30 Mb
Rosmarinus officinalis	[7]	1180	85.8%	90.1%	21 Kb	368 Kb
Salvia bowleyana	[14]	538	95.6%	92.2%	1.1 Mb	58 Mb
Saliva miltiorrhiza	[15]	615	87.5%	n.r.	12 kb	51 Kb
	[16]	645	99.2%	n.r.	82 kb	1.2 Mb
	*, [17]	600	99.1%	92.5%	2.7 Mb	69 Mb
	*, [18]	557	91.5%	91.1%	500 Kb	1.2 Mb
Salvia splendens	[19]	711	92.2%	92.1%	2.2 Mb	3.1 Mb
	[20]	807	95.0%	92.0%	3.8 Mb	35.1 Mb
Scutellaria baicalensis	[21]	408	93.0%	94.0%	1.3 Mb	33.2 Mb
	*, [22]	440	85.3%	91.5%	2.1 Mb	40.8 Mb
Scutellaria barbata	[22]	405	87.2%	93.0%	2.5 Mb	23.7 Mb
Tectona grandis	[23]	465	79.8%	n.r.	277 Kb	357 Kb
	*, [24]	325	104.0%	92.3%	3 Mb	16 Mb
Thymus quinquecostatus	[25]	536	98.5%	97.4%	n.r.	8 Mb

Supplementary Table 2. Supernova genome assembly metrics for *Leonotis leonurus*, *Plectranthus barbatus*, and *Prunella vulgaris*.

	Leonotis leonurus	***Plectranthus barbatus***	***Prunella vulgaris***
Number of reads	297.57 M	531.03 M	360.00 M
Raw coverage	64.90x	61.72x	61.95x
Effective read coverage	48.06x	42.62x	41.80x
Fraction of Q30 bases in read 2	92.32%	92.17%	91.67%
Median insert size	389 bp	387 bp	412 bp
Fraction of proper read pairs	92.65%	92.38%	92.59%
Estimated genome size	687.80 Mb	1.29 Gb	871.63 Mb
Mean distance between heterozygous SNPs	16.90 Kb	961 bp	272 bp
Long Scaffolds	4.44 K	12.72 K	8.55 K
Edge N50	12.02 Kb	13.51 Kb	14.95 Kb
Contig N50	69.69 Kb	44.24 Kb	50.23 Kb
Phaseblock N50	23.56 Kb	374.06 Kb	673.35 Kb
Scaffold N50	1.35 Mb	314.68 Kb	626.96 Kb
Missing 10 kb	24.71%	9.76%	15.33%
Assembly size	482.20 Mb	979.55 Mb	646.84 Mb

Supplementary Table 3. Gene annotation metrics for *Leonotis leonurus, Plectranthus barbatus*, and *Prunella vulgaris*.

	Leonotis leonurus	***Plectranthus barbatus***	***Prunella vulgaris***
Number of genes	148,846	413,222	229,613
Mean gene length	2,045	1,617	1,746
Mean exon length	376	381	368
Mean intron length	405	350	339
Mean CDS length	1,172	1,020	1,078
Mean exons per mRNA	3	3	3
Mean introns per mRNA	2	2	2

Supplementary Table 4. Repetitive sequencing in *Leonotis leonurus, Plectranthus barbatus*, and *Prunella vulgaris* genomes.

	L. leonurus		***P. barbatus***		***P. vulgaris***	
	**Total length(bp)**	**Percentage of sequence**	**Total length(bp)**	**Percentage of sequence**	**Total length(bp)**	**Percentage of sequence**
Masked Repetitive Sequence	338,798,899	57.89%	930,364,827	74.43%	560,464,006	68.33%
Retroelements	151,474,504	25.88%	544,678,180	43.58%	315,027,174	38.41%
DNA transposons	21,417,562	3.66%	22,952,357	1.84%	20,418,154	2.49%
Rolling-circles	1,477,372	0.25%	2,827,808	0.23%	2,002,766	0.24%
Unclassified	162,943,174	27.84%	358,986,403	28.72%	222,203,816	27.09%
Small RNA	978,284	0.17%	487,208	0.04%	385,209	0.05%
Satellites	100,172	0.02%	8,107	0.00%	90,361	0.01%
Simple repeats	402,036	0.07%	422,102	0.03%	334,293	0.04%
Low complexity	7,327	0.00%	3,487	0.00%	5,921	0.00%

Supplementary Table 5. Primers used in this study.

Gene	Direction	Sequence (5’-3’)
CamCYP76AH64	F	ATGGATTTCTTTGCTCTTTTCG
CamCYP76AH64	R	CTATGGTTCACACTTGATTGGA
CamCYP76AH65	F	ATGGATCTCTTTGAGCTCCTCGTT
CamCYP76AH65	R	TCAGAGCTTGATCGGAGTAGCCTT
CamCYP76AH66	F	ATGGATTTCTTTGCATTTTTTATTG
CamCYP76AH66	R	TCATGGTTTGATAGGAATTGC
CamCYP76AH67	F	GAACAAATTGAGATGGATCT
CamCYP76AH67	R	CATATTTCATAAGATCAAGGCT
CamCYP76AH68	F	ATGGATCTCTATGCCCTTTT
CamCYP76AH68	R	ATATTTCACAAAATCATCAGGG
CamCYP76AH69	F	CACTAGTCTCATGGAGTTT
CamCYP76AH69	R	ATAATCATTTGACCTGCTTGAAA
CamCYP71D716	F	TTTGTCAACCTTATGGAGTTT
CamCYP71D716	R	AAAAGGTCTCCTAACAGTG
CamCYP71D717	F	ATGGAGTTTGAGTTCCCATC
CamCYP71D717	R	TCATTTGACCTGCTTGAAAG
CamTPS7	F	ATGACCTCTATATCCACTGTTCT
CamTPS7	R	TCATACAACTGGTTCAAATAGTACT
CamTPS9	F	ATGTCACTCAGTTTCAACCTCA
CamTPS9	R	GCTTCATGAGGAGGAAGGAAGT
CamTPS10	F	ACCAATCCCATGAAACTTTCAT
CamTPS10	R	TATACATAAACCAAAATACCCAAATCG

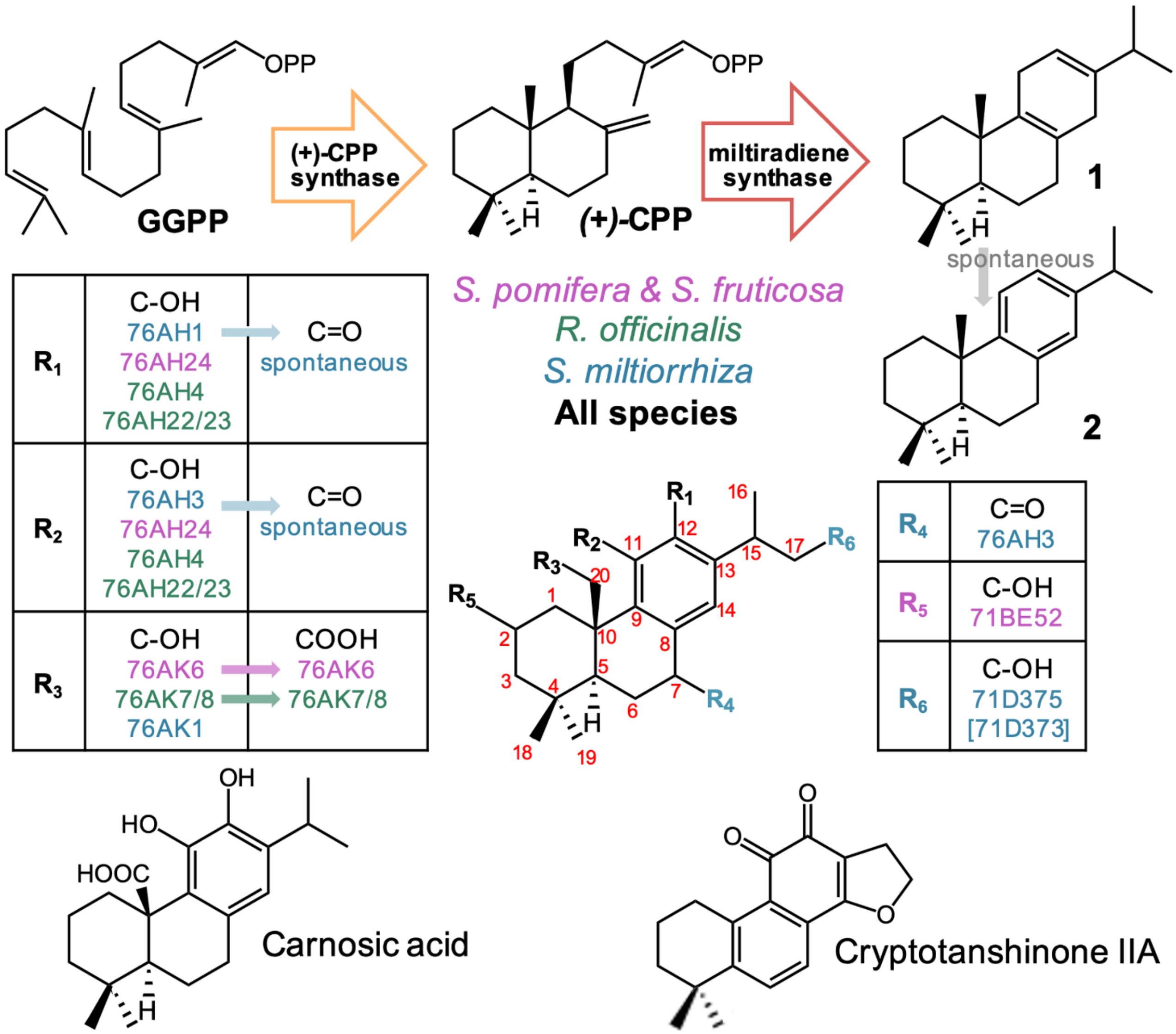

Supplementary Figure 1. Summary of oxidation positions for CYPs involved in the biosynthesis of tanshinones (*S. miltiorrhiza*), carnosic acid (*S. pomifera, S. fruticosa, R. officinalis*), and related diterpenoids. These CYPs have been shown to be part of a metabolic network, and intermediates required for sequential oxidation are condensed here for brevity. Aromatization of miltiradiene to abietatriene, the precursor for most oxidations shown, is presumed to be spontaneous. Enzymes from different species (color code in figure) that oxidize at the same position are also orthologous.

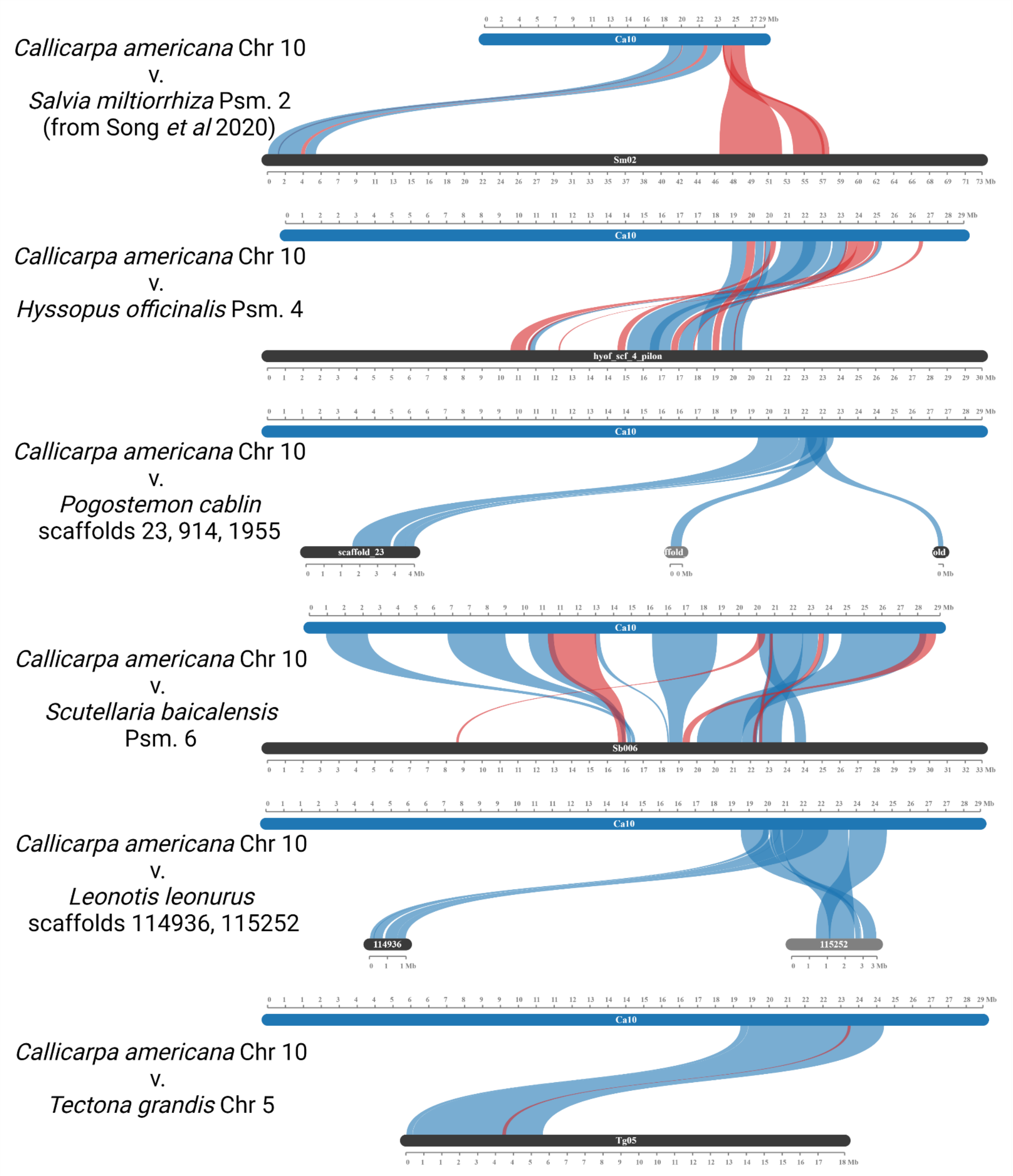

Supplementary Figure 2. Chromosome/Pseudomolecule/Scaffold-level synteny of C. americana and compared to each other species containing the syntenic BGC. Forward syntenic blocks are shown in blue; reverse in red. Note that there are larger syntenic blocks surrounding the region containing the BGC. Source data are provided as a Source Data file.

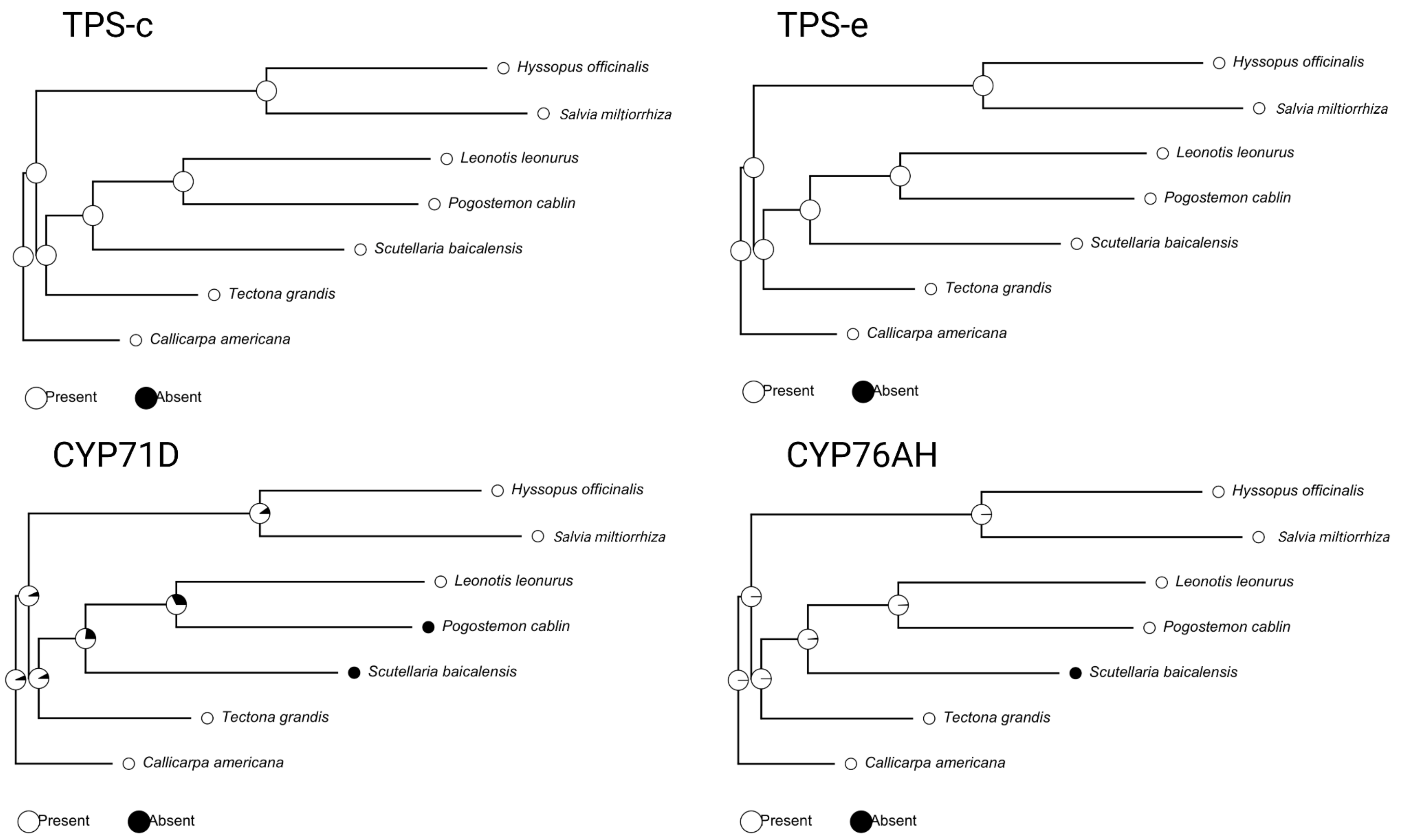

Supplementary Figure 3. Ancestral reconstruction of syntenic genes present across all examined species. Circles at each node represent presence/absence (white/black, respectively) of each gene. Analysis was performed using the R package phytools.

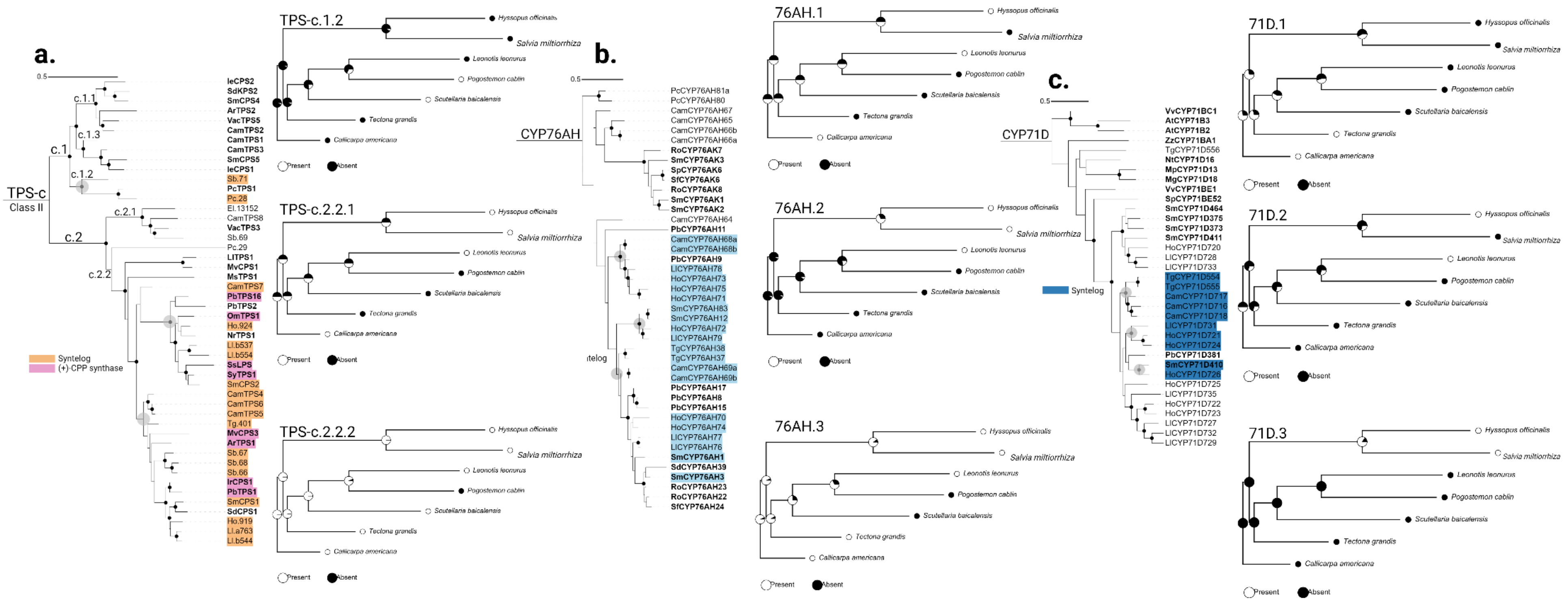

Supplementary Figure 4. Ancestral reconstruction of syntenic genes present across all examined species in a lineage-central manner. (a) TPS-c class II diTPS and clades with ancestral reconstruction. (b) Cytochrome P450 subfamily CYP76AH/CYP76AK and clades with ancestral reconstruction. (c) Cytochrome P450 subfamily CYP71D and clades with ancestral reconstruction. Circles at each node represent presence/absence (white/black, respectively) of each gene. Each ancestral reconstruction is represented by gray nodes on the main enzyme phylogeny. Analysis was performed using the R package phytools. Syntelogs and (+)-CPP synthases color coded as given in figure.

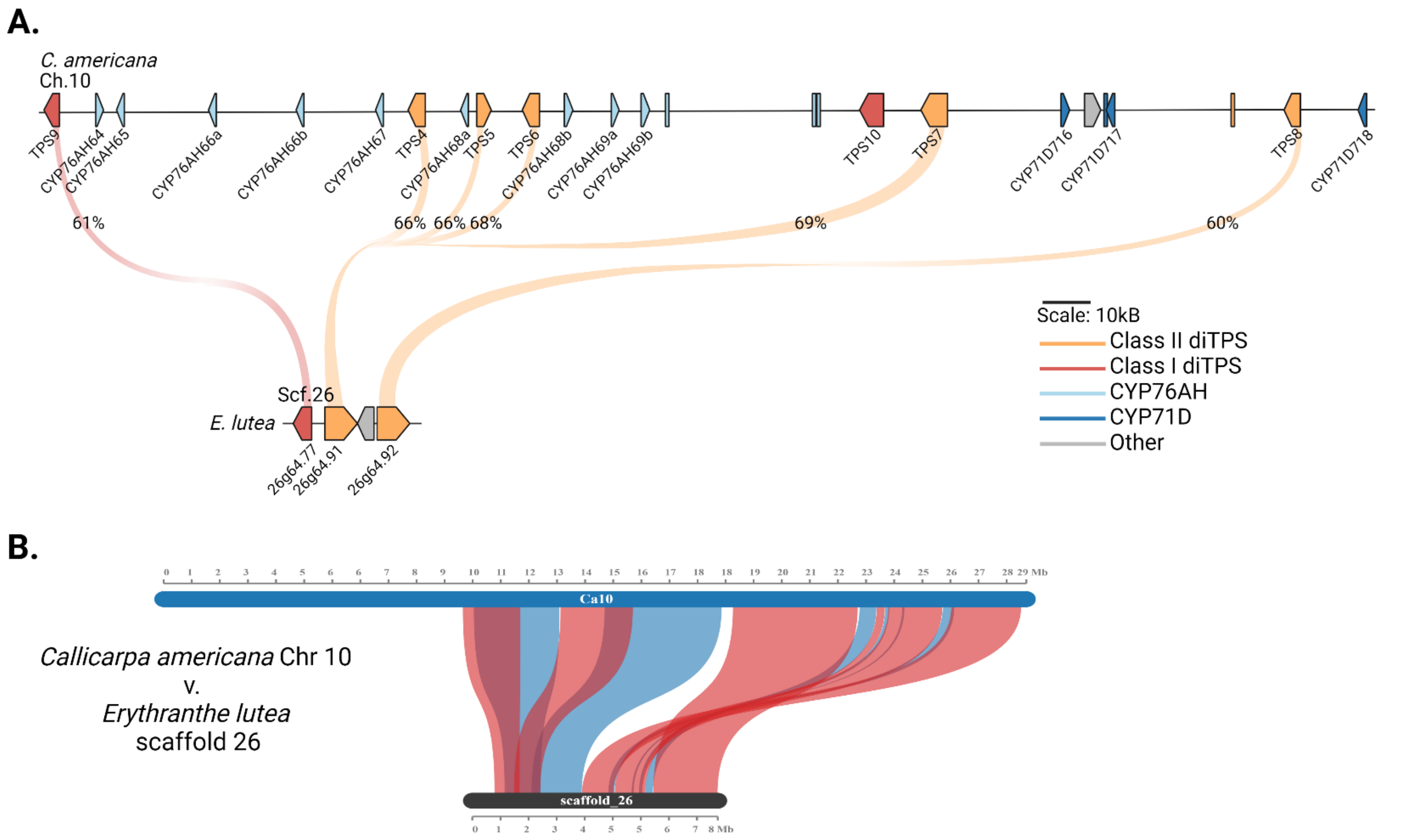

Supplementary Figure 5. There appears to be a related miltiradiene cluster-like region in close Lamiales outgroup *Erythranthe lutea*. (**a**) Orthologs between C. *americana* and *E. lutea* shown at the gene level. Percent identity (BLASTp algorithm) noted on each line. Color code for TPSs, CYPs and other genes as given in the figure. (**b**) Synteny between *C. americana* chromosome 10 and *E. lutea* scaffold 26 where the syntenic genes are present. Forward syntenic blocks are shown in blue; reverse in red. We believe this BGC may predate the Lamiaceae based on evidence shown here; however, a more widespread examination of additional Lamiales is needed to provide more support.

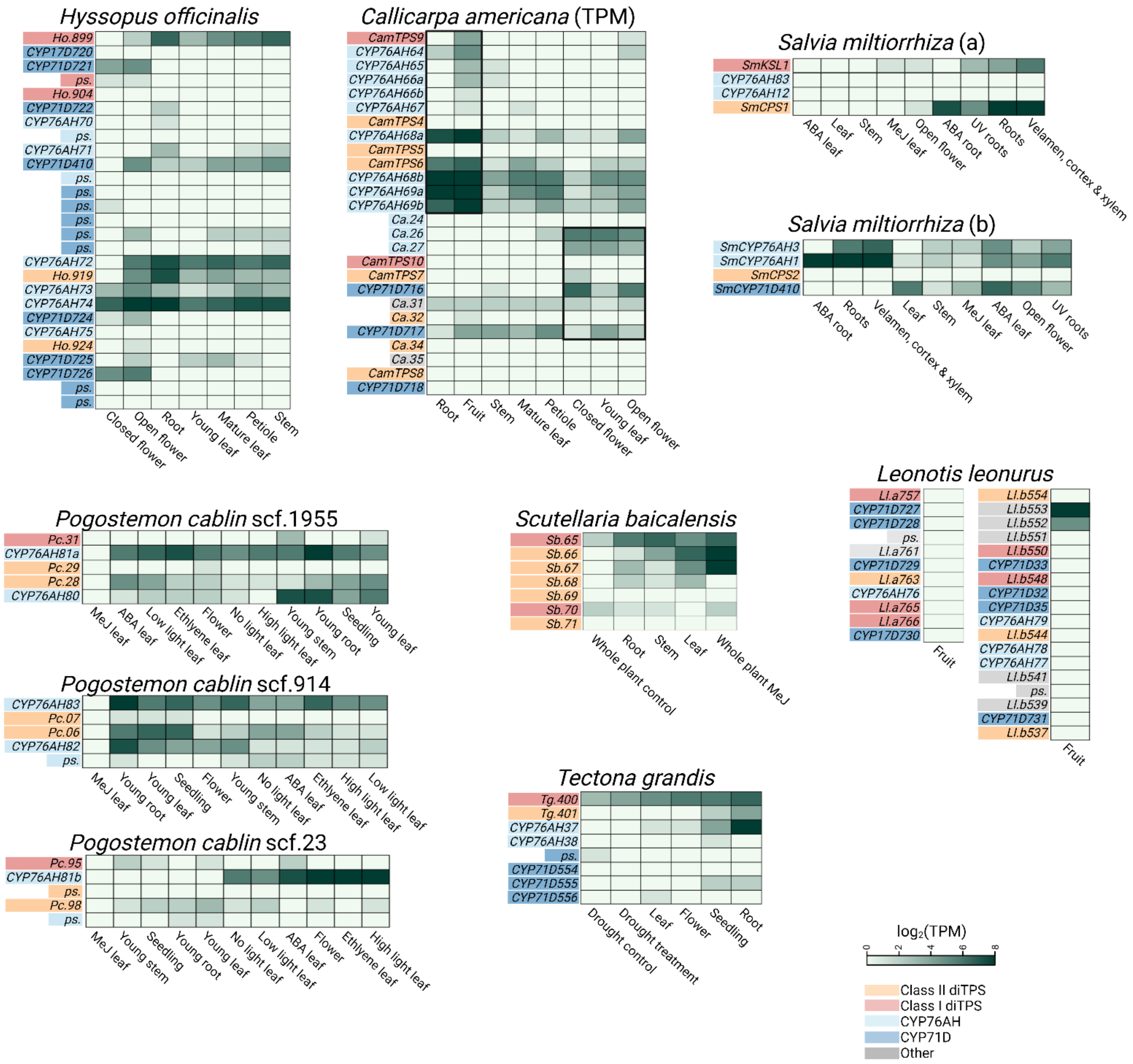

Supplementary Figure 6. Expression patterns of each syntenic BGC explored. Data shown in transcript per million. Source data are provided as a Source Data file.

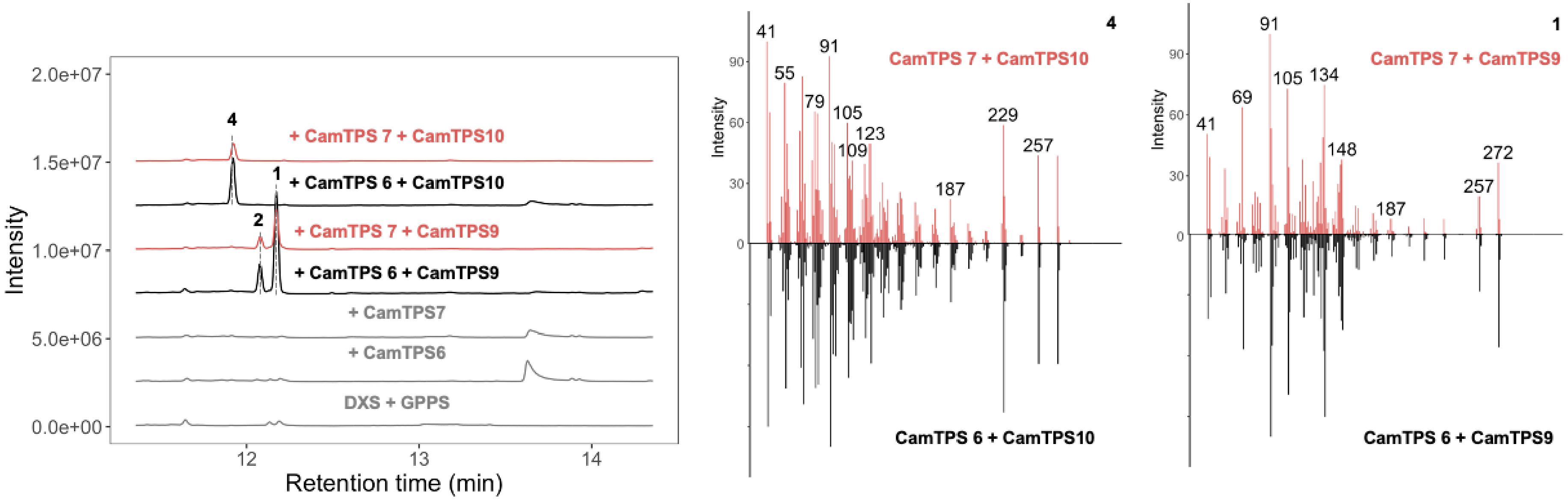

Supplementary Figure 7. Characterization of CamTPS7 as a (+)-CPP synthase by comparison with CamTPS6. Both produce 1 & 2 when paired with CamTPS9 (miltiradiene synthase) and 4 when paired with CamTPS10 ((+)-kaurene synthase). Source data are provided as a Source Data file.

^{13}C NMR	δ	^{1}H NMR	δ
1	39.5	$1H_a$<br>$1H_b$	1.64<br>0.78
2	18.48	$1H_a$<br>$1H_b$	1.36<br>1.56
3	42.04	$1H_a$<br>$1H_b$	1.37<br>1.13
4	33.22	-	-
5	56.56	1H	0.83
6	20.31	$1H_a$<br>$1H_b$	1.23<br>1.52
7	34.02	$1H_a$<br>$1H_b$	1.57<br>1.49
8	43.66	-	-
9	56.94	1H	1.1
10	37.87	-	-
11	19.01	2H	1.48
12	41.06	2H	1.51
13	42.72	1H	2.52
14	50.36	$1H_a$<br>$1H_b$	1.5<br>1.15
15	41.48	$1H_a$<br>$1H_b$	2.76<br>1.8
16	157.81	-	
17	102.3	$1H_a$<br>$1H_b$	4.73<br>4.7
18	21.9	3H	0.8
19	33.69	3H	0.85
20	15.12	3H	0.91

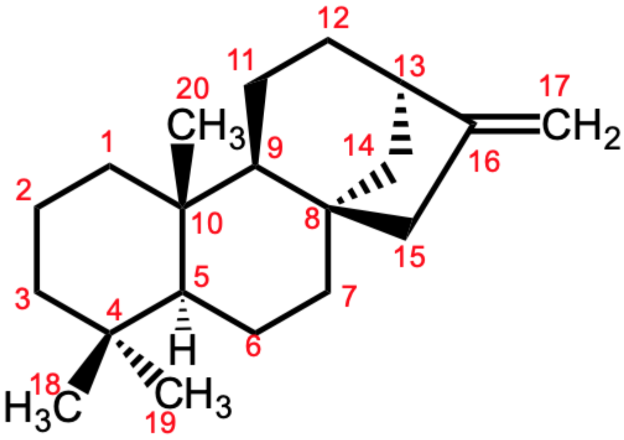

Supplementary Figure 8. NMR analysis confirms that 4 is (+)-kaurene. Connectivity was deduced from ^{1}H, ^{13}C, HSQC, HMBC, and COSY correlations (see Supplementary Fig. 9-10). CDCl3 was used as the solvent, and CDCl3 peaks were referenced to 7.26 and 77.00 ppm for ^{1}H and ^{13}C spectra, respectively. Relative stereochemistry was assigned based on NOESY correlations. Selected NOESY correlations are shown on a 3D representation of (+)-kaurene generated with the Cahn–Ingold–Prelog CIP Tool[22]. Absolute stereochemistry was assigned based on the configuration of (+)-CPP, the precursor of 4.

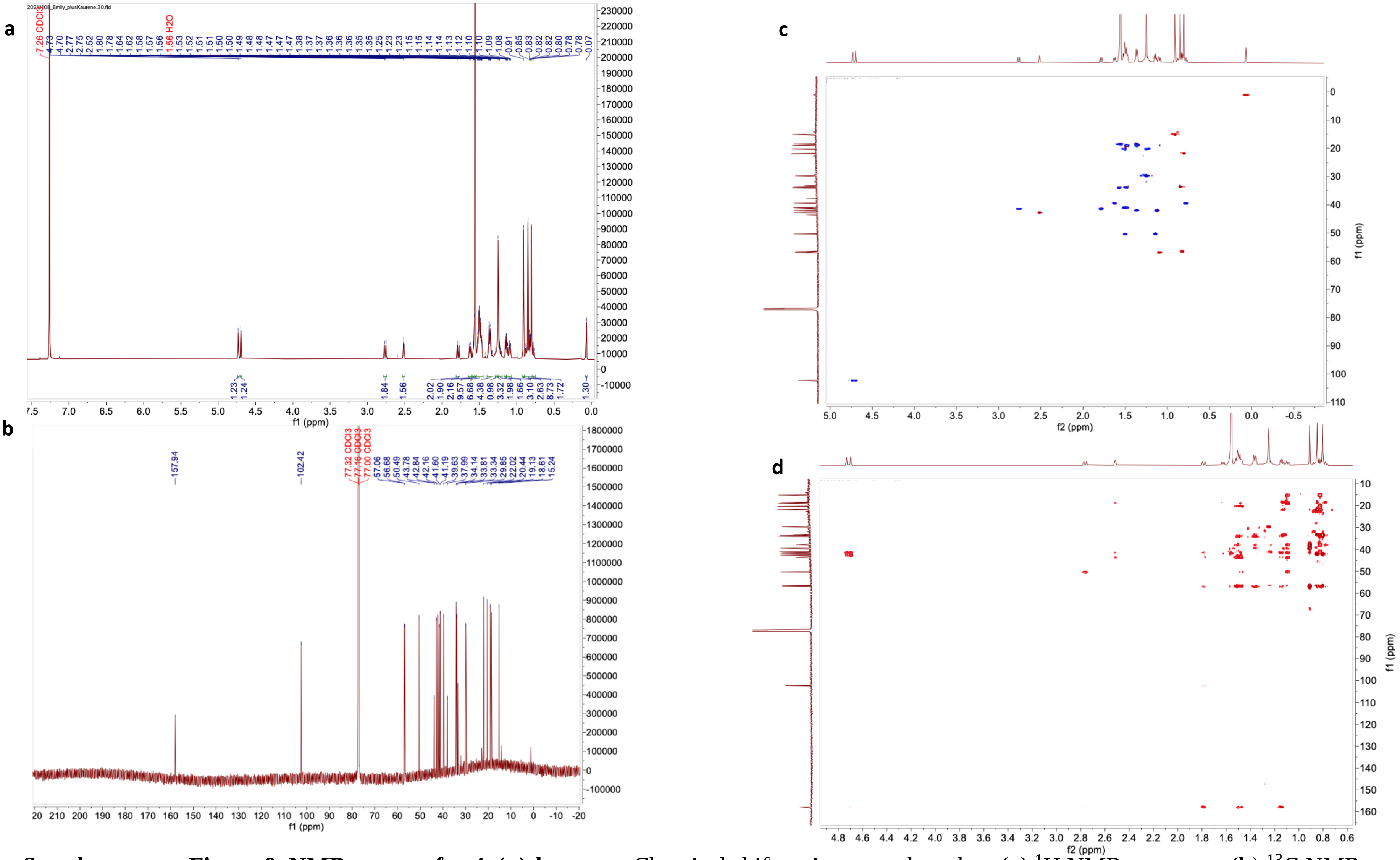

Supplementary Figure 9. NMR spectra for 4, (+)-kaurene. Chemical shift assignments based on (**a**) ^{1}H NMR spectrum, (**b**) ^{13}C NMR spectrum, (**c**) HSQC spectrum, and (**d**) HMBC spectrum. CDCl3 was used as the solvent, and CDCl3 peaks were referenced to 7.26 and 77.00 ppm for ^{1}H and ^{13}C spectra, respectively.

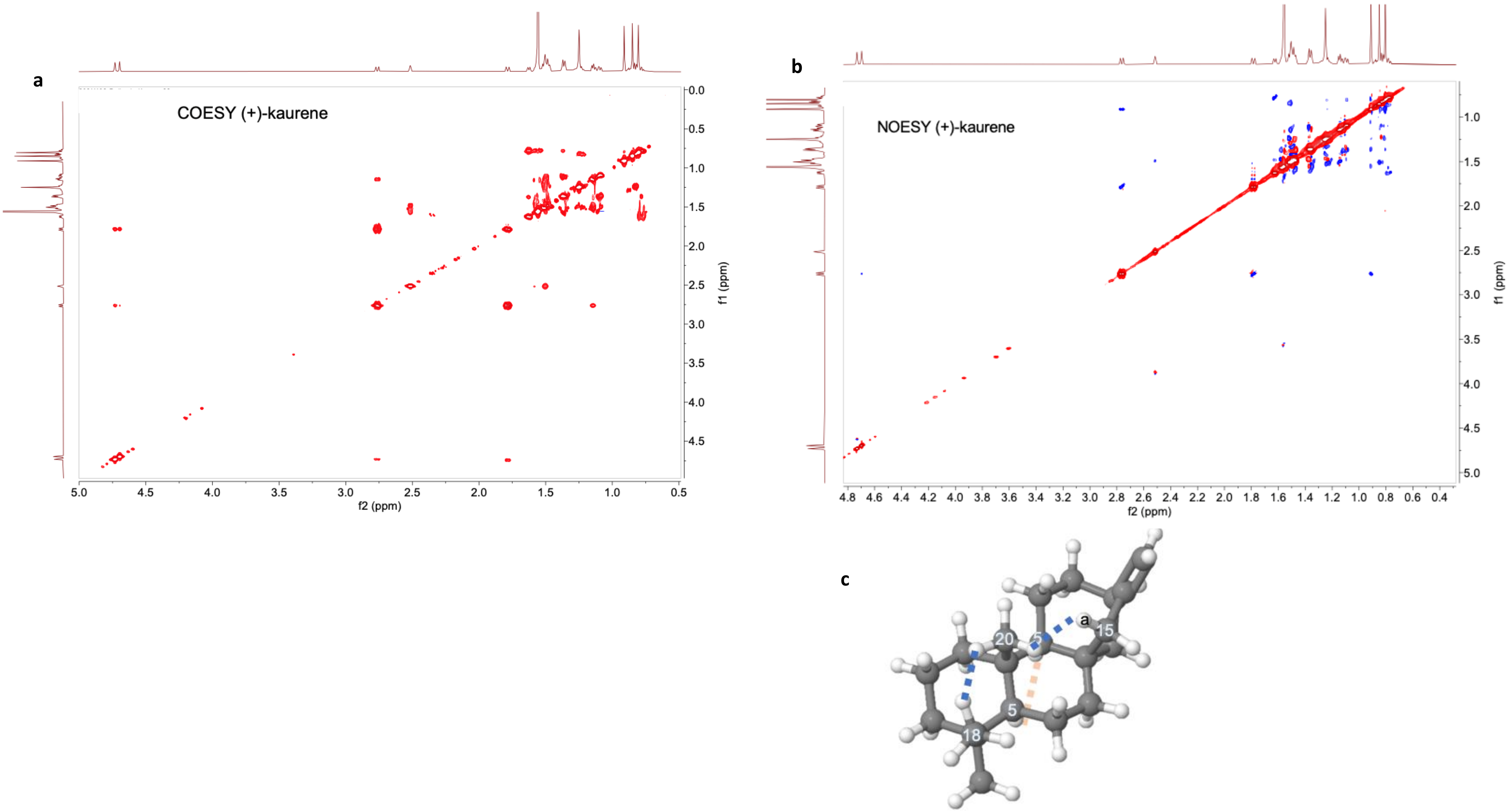

Supplementary Figure 10. Additional NMR spectra for 4, (+)-kaurene. Chemical shift assignments assisted by the (**a**) COSY NMR spectrum, and relative stereochemistry was assigned based on key NOESY (**b**) correlations. Selected NOESY correlations are shown on a 3D representation of (+)-kaurene (**c**) generated with the Cahn–Ingold–Prelog CIP Tool.[27]

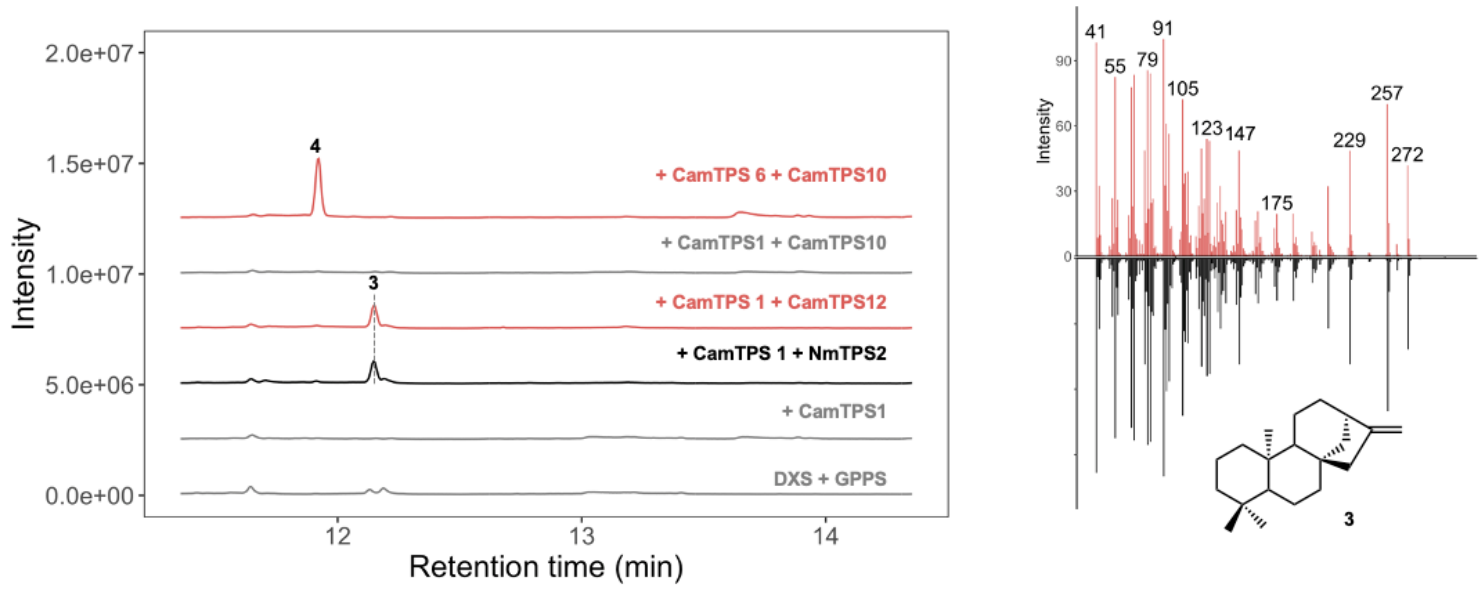

Supplementary Figure 11. Characterization of CamTPS12 as an *ent*-kaurene synthase by comparison with the product of NmTPS2[23] when combined with the *ent*-CPP synthase CamTPS1[1]. CamTPS12 was tested alongside NmTPS223, a known ent-kaurene synthase, in combination with the previously characterized *ent*-CPP synthase CamTPS11. Matching retention times (that differ from **4**, (+)-kaurene) and mass spectra for **3** confirmed the activity of CamTPS12. Source data are provided as a Source Data file.

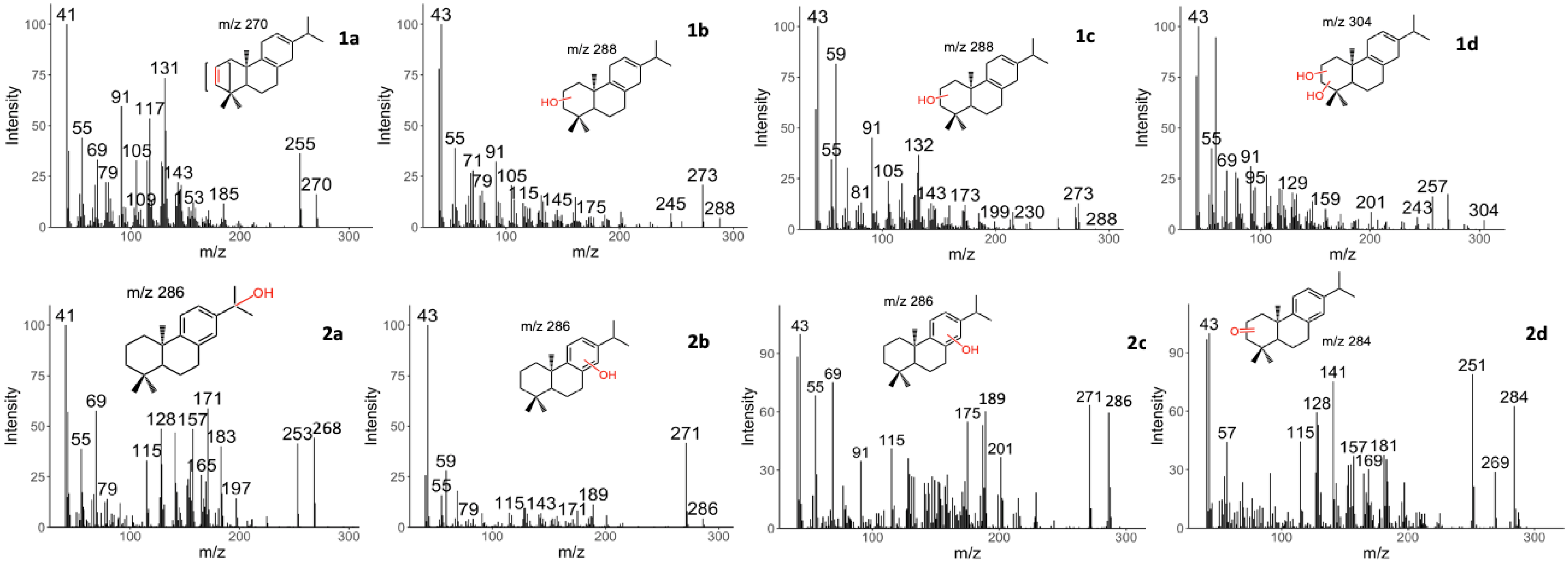

Supplementary Figure 12. Mass spectra of the CYP76AH oxidation products. EI mass spectrum of each product along with predicted structural information. Assignment of terpene backbone as abietatriene or miltiradiene is based on fragmentation pattern and visible molecular ion. NMR was only possible for **2a**, and the assigned structure is shown. **1a** closely matches the spectrum of miltiradiene shifted by (-2) m/z, consistent with an additional desaturation. However, this could also be the miltiradiene analogue of **2a**, which similarly does not have the hydroxylated molecular ion. **1b** and **1c** display molecular ions consistent with a single oxidation of miltiradiene while **1d** appears to be a doubly hydroxylated analogue. **2b** and **2c** have a molecular ion of 286, which matches a single oxidation of abietatriene. **2d** has a molecular ion of 284, consistent with keto-abietatriene. Source Data are provided as a Source Data file.

^{13}C NMR	δ	^{1}H NMR	δ
1	38.83	$1H_a$<br>$1H_b$	1.42<br>2.31
2	19.3	$1H_a$<br>$1H_b$	1.62<br>1.76
3	41.7	$1H_a$<br>$1H_b$	1.24<br>1.5
4	33.47	-	
5	50.38	1H	1.36
6	19.08	$1H_a$<br>$1H_b$	1.7<br>1.91
7	30.62	$1H_a$<br>$1H_b$	2.92<br>2.92
8	135.01	-	
9	145.73	-	
10	37.58	-	

^{13}C NMR	δ	^{1}H NMR	δ
11	124.31	1H	7.18
12	121.85	1H	7.24
13	148.71	-	
14	124.95	1H	7.18
15	72.3	-	
16	31.66	3H	1.59
17	31.66	3H	1.59
18	33.27	3H	0.98
19	21.67	3H	0.95
20	24.89	3H	1.21

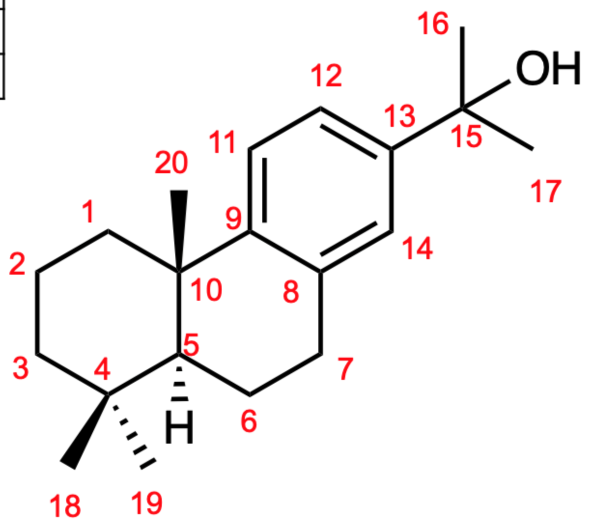

Supplementary Figure 13. NMR chemical shift assignments of (2a), 15-hydroxy-*ent*-abiet-8-11-13-triene. Connectivity was deduced from ^{1}H, ^{13}C, HSQC, HMBC, and COSY correlations (Supplementary Fig. 14-15) . CDCl3 was used as the solvent, and CDCl3 peaks were referenced to 7.26 and 77.00 ppm for ^{1}H and ^{13}C spectra, respectively. Absolute stereochemistry was assigned based on the configuration of abietatriene, the precursor of **2a**. NMR data also closely match previously published data for this compound[26].

a

b

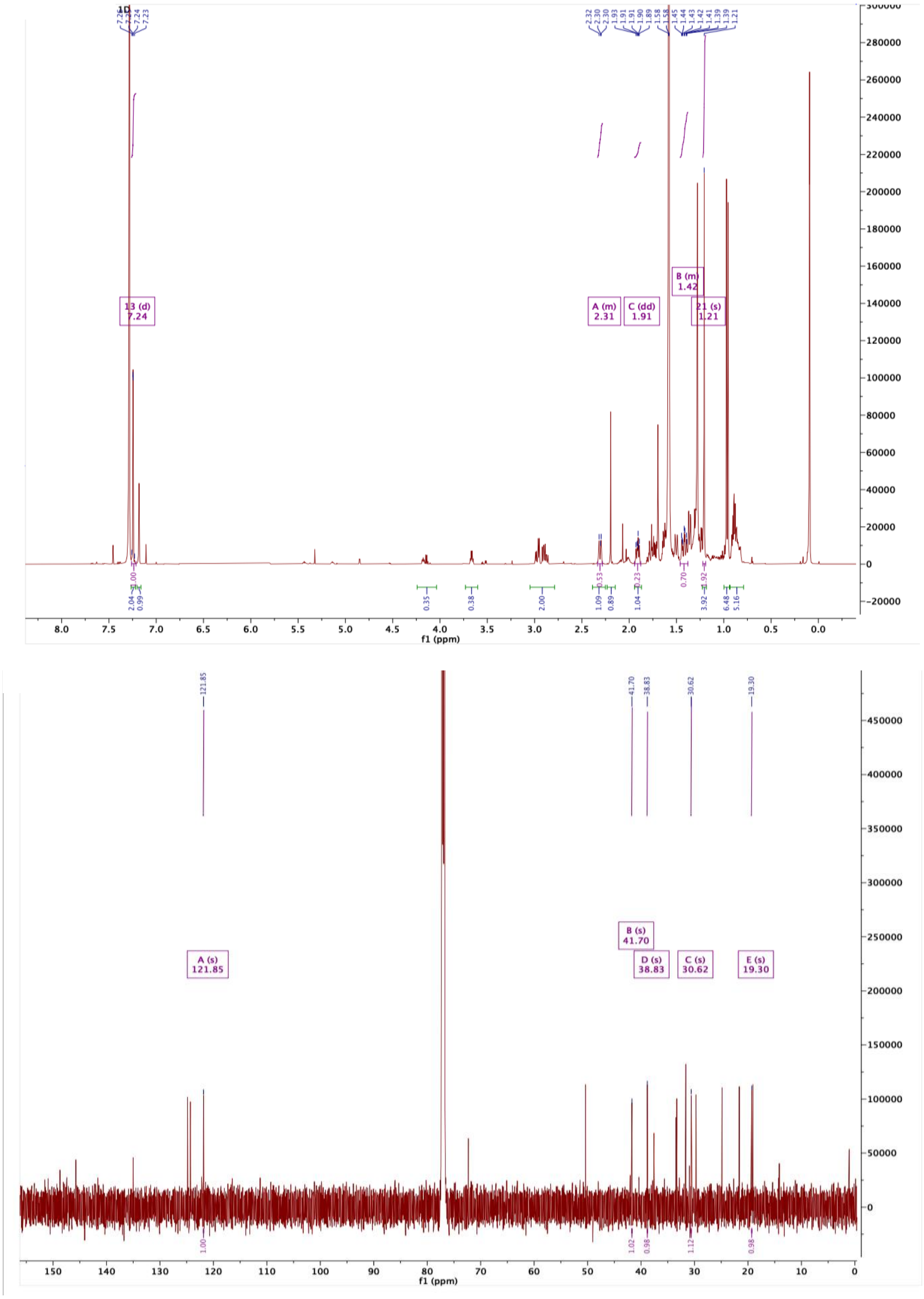

Supplementary Figure 14. NMR spectra of 15-hydroxy-*ent*-abiet-8-11-13-triene. (**a**)^{1} H NMR spectrum and (**b**) ^{13}C NMR spectrum. CDCl3 was used as the solvent, and CDCl3 peaks were referenced to 7.26 and 77.00 ppm for ^{1}H and ^{13}C spectra, respectively.

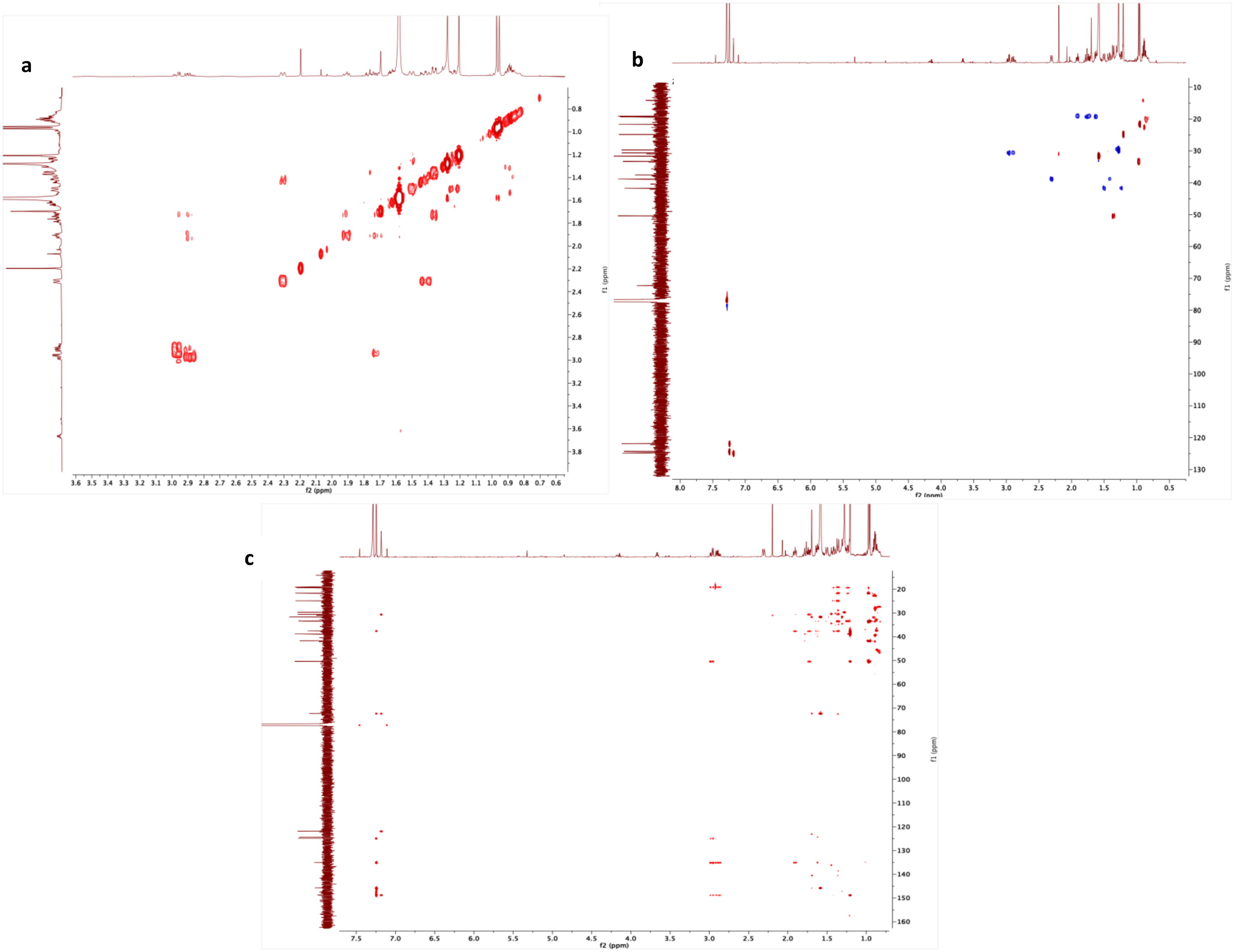

Supplementary Figure 15. Additional NMR spectra of 15-hydroxy-*ent*-abiet-8-11-13-triene. The (**a**) COSY NMR spectrum, (**b**) HSQC spectrum, and (**c**) HMBC spectrum aided in assigning the structure of **2a.** CDCl3 was used as the solvent, and CDCl3 peaks were referenced to 7.26 and 77.00 ppm for ^{1}H and ^{13}C spectra, respectively.

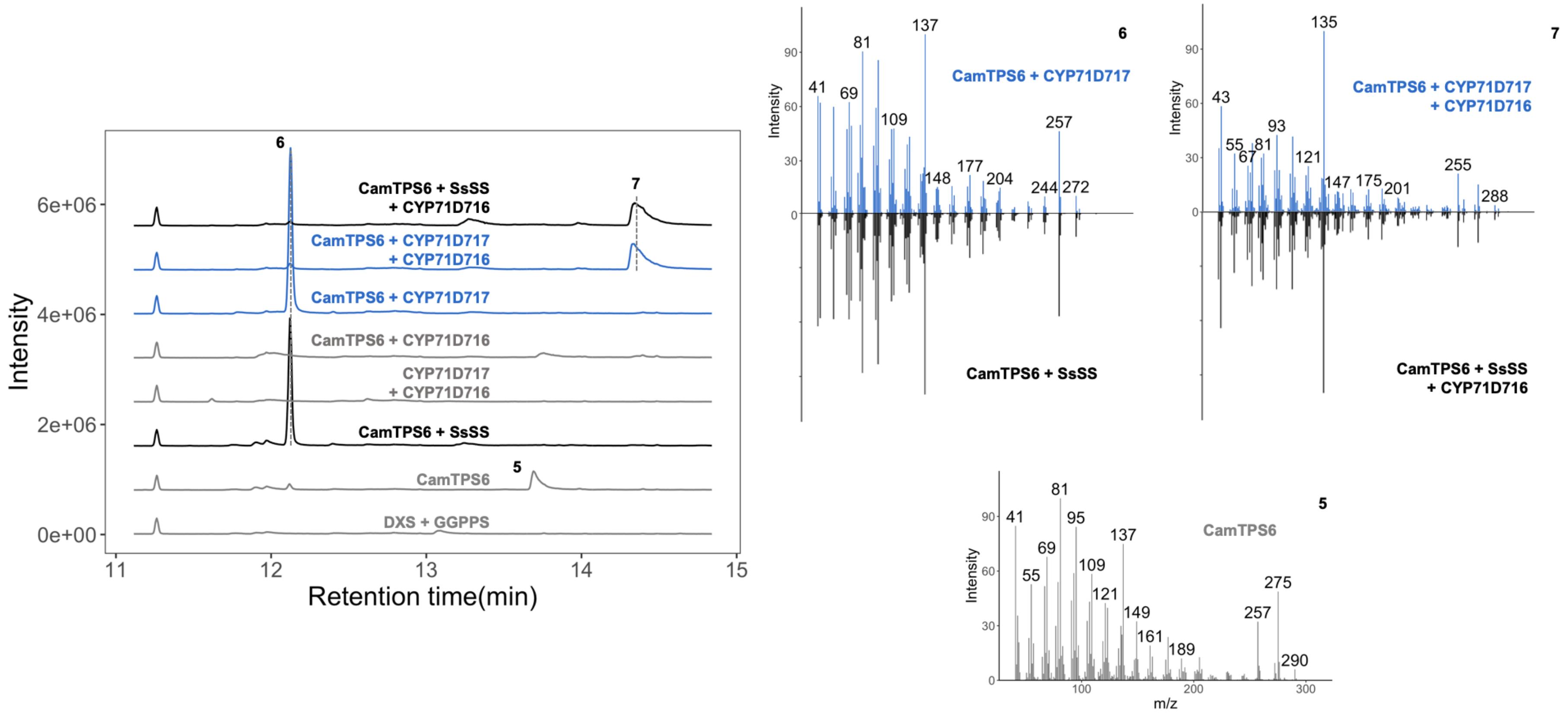

Supplementary Figure 16. Further analysis of CamCYP71D717 shows that it unexpectedly functions as a (+)-manool synthase when expressed in *N. benthamiana*. We compared retention times and mass spectra of products when SsSS, a promiscuous class I TPS known to afford (+)-manool from (+)-CPP[24], was substituted for CamCYP71D17. Based on recent work demonstrating CYP oxidation of kolavenol but not kolavenyl diphosophate[25], we suggest that CamCYP71D717 is catalyzing an isomerization of (+)-copalol (6) which is produced by endogenous phosphatases in *N. benthamiana* when a (+)-CPP synthase is expressed. Source data are provided as a Source Data file.

^{13}C NMR	δ	^{1}H NMR	δ
1	37.05	1H$_a$<br>1H$_b$	1.75<br>1.12
2	27.91	1H$_a$<br>1H$_b$	1.52<br>1.62
3	78.89	1H<br>1OH	3.19
4	39.13	-	
5	54.61	1H	1.01
6	23.99	1H$_a$<br>1H$_b$	1.3<br>1.67
7	38.07	1H$_a$<br>1H$_b$	2.34<br>1.89
8	148.1	-	
9	56.88	1H	1.46
10	39.56	-	

^{13}C NMR	δ	^{1}H NMR	δ
11	17.8	1H$_a$<br>1H$_b$	1.46<br>1.24
12	41.2	1H$_a$<br>1H$_b$	1.24<br>1.64
13	73.67	-	
14	145.05	1H	5.83
15	111.74	1H$_a$<br>1H$_b$	5.13<br>5.0
16	28.17	3H	1.2
17	106.79	1H$_a$<br>1H$_b$	4.76<br>4.42
18	15.41	3H	0.7
19	28.31	3H	0.92
20	14.47	3H	0.61

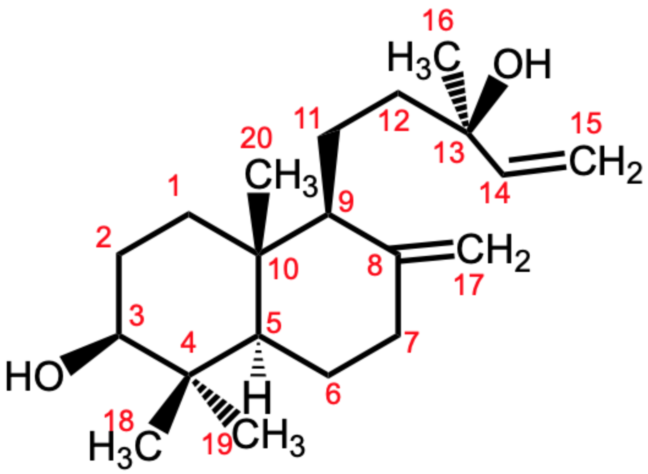

Supplementary Figure 17. NMR analysis of 7 supports assignment as 3(S)-hydroxy-(+)-manool. Connectivity was deduced from ^{1} H, ^{13}C, HSQC, HMBC, and COESY correlations. CDCl3 was used as the solvent, and CDCl3 peaks were referenced to 7.26 and 77.00 ppm for ^{1}H and ^{13}C spectra, respectively. Selected NOESY correlations are shown to support assignment of relative stereochemistry. Absolute stereochemistry was assigned based on the configuration of (+)-CPP, the precursor of **7**.

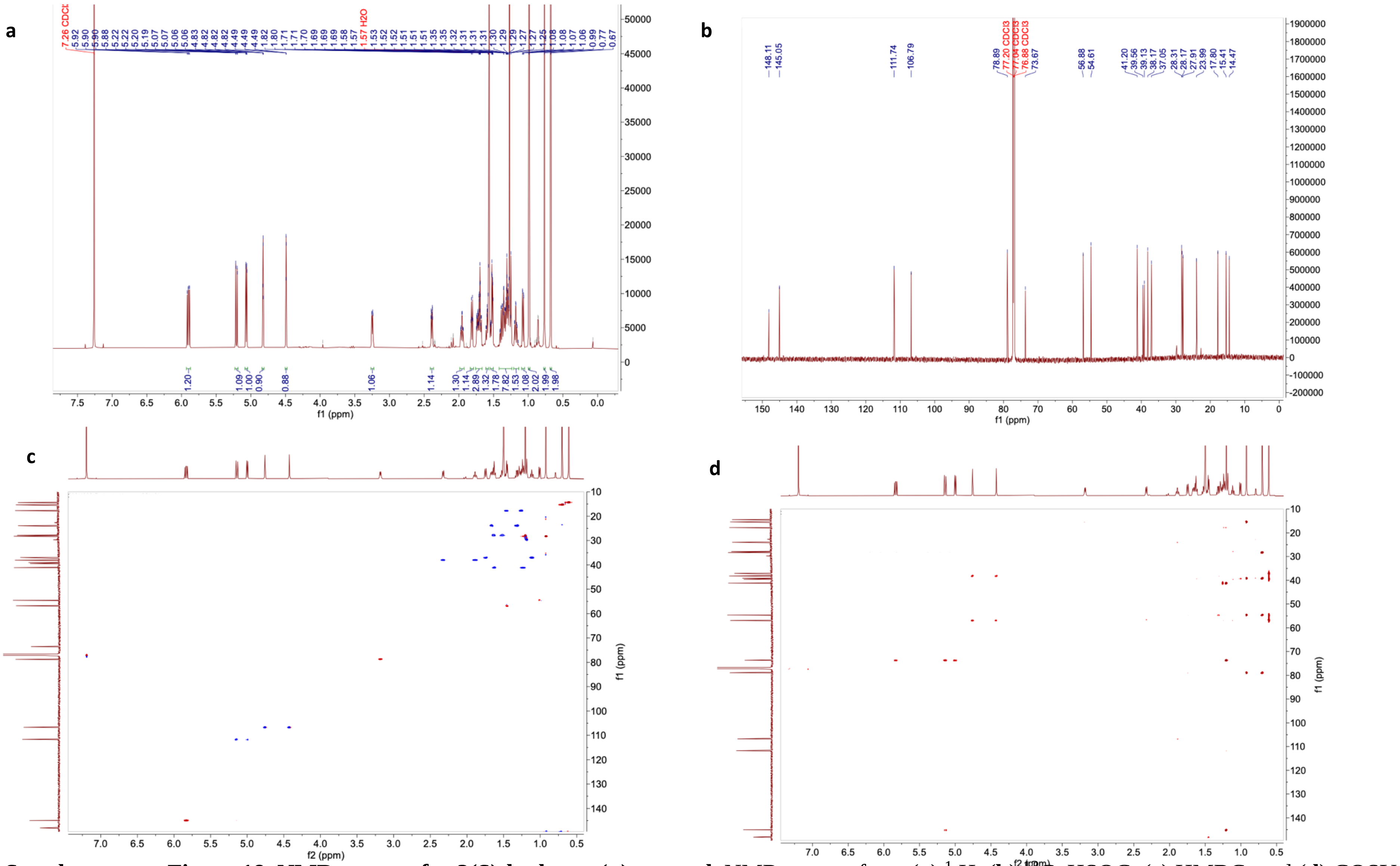

Supplementary Figure 18. NMR spectra for 3(S)-hydroxy-(+)-manool. NMR spectra from (**a**) ^{1}H, (**b**) ^{13}C, HSQC, (**c**) HMBC, and (**d**) COSY experiments. CDCl3 was used as the solvent, and CDCl3 peaks were referenced to 7.26 and 77.00 ppm for ^{1}H and ^{13}C spectra, respectively.

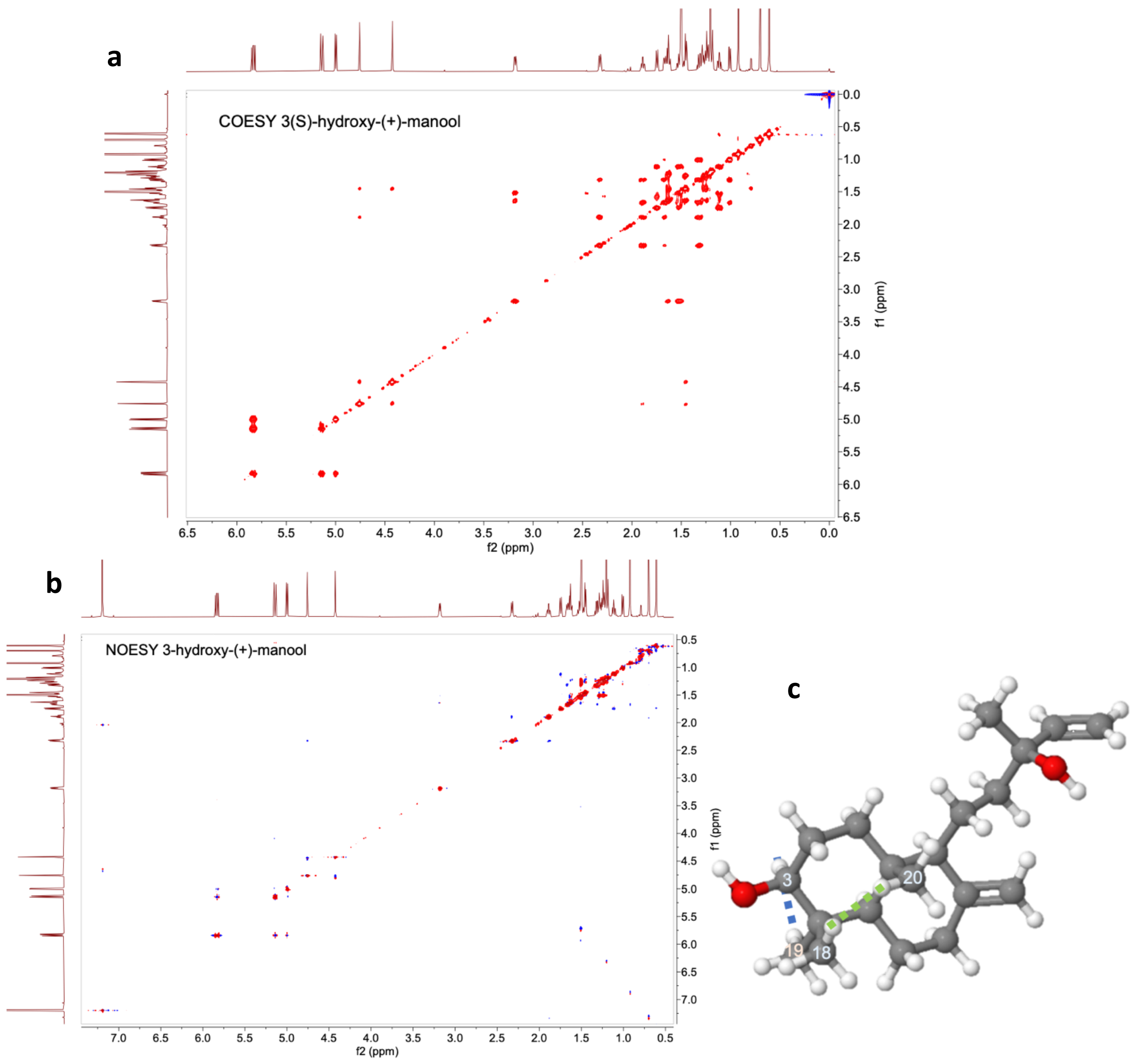

Supplementary Figure 19. Additional NMR spectra for 3(S)-hydroxy-(+)-manool. Chemical shift assignments assisted by the (**a**) COSY NMR spectrum, and relative stereochemistry was assigned based on key NOESY (**b**) correlations. Selected NOESY correlations are shown on a 3D representation of 3(S)-hydroxy-(+)-manool (**c**) generated with the Cahn–Ingold–Prelog CIP Tool[27].

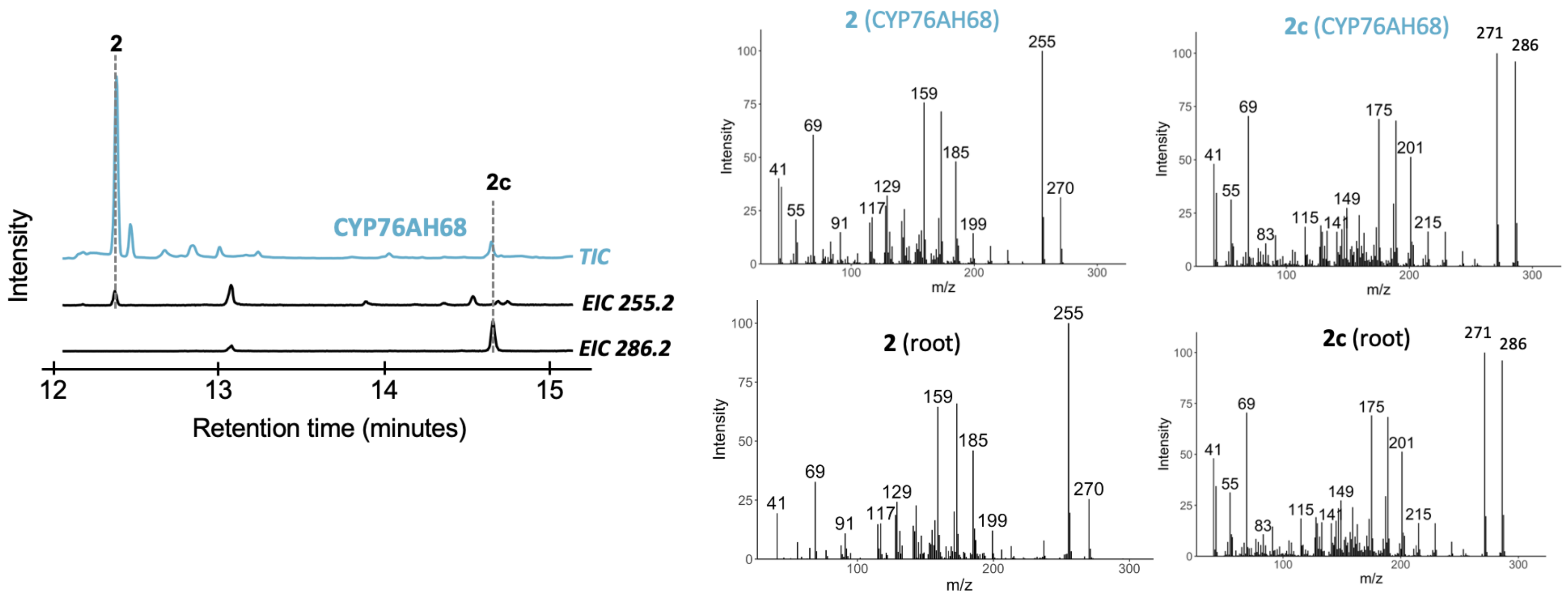

Supplementary Fig. 20. GC-MS analysis of an ethyl acetate root extract from *C. americana*. The root extract was analyzed alongside the products of CYP76AH68. Extracted ion chromatograms of m/z 255 and m/z 286 show two co-eluting peaks, both of which have matching mass spectra between the root extract and CYP76AH68 experiment. The first appears to be abietatriene (**2**) and the second (**2c**) is likely ferruginol or a close analogue based on its best match in the NIST (v2.3) database. Source data are provided as a Source Data file.

Supplementary references

1. Hamilton, J. P. *et al.* Generation of a chromosome-scale genome assembly of the insect-repellent terpenoid-producing Lamiaceae species, *Callicarpa americana*. *GigaScience* **9**, giaa093 (2020).
2. Lichman, B. R. *et al.* The evolutionary origins of the cat attractant nepetalactone in catnip. *Sci. Adv.* **6**, (2020).
3. Malli, R. P. N., Adal, A. M., Sarker, L. S., Liang, P. & Mahmoud, S. S. *De novo* sequencing of the *Lavandula angustifolia* genome reveals highly duplicated and optimized features for essential oil production. *Planta* **249**, 251–256 (2019).
4. Li, J. *et al.* The chromosome-based lavender genome provides new insights into Lamiaceae evolution and terpenoid biosynthesis. *Hortic. Res.* **8**, 1–14 (2021).
5. Vining, K. J. *et al.* Draft genome sequence of *Mentha longifolia* and development of resources for mint cultivar improvement. *Mol. Plant* **10**, 323–339 (2017).
6. Vining, K. J. *et al.* Chromosome-level genome assembly of *Mentha longifolia* L. reveals gene organization underlying disease resistance and essential oil traits. *G3 GenesGenomesGenetics* jkac112 (2022).
7. Bornowski, N. *et al.* Genome sequencing of four culinary herbs reveals terpenoid genes underlying chemodiversity in the Nepetoideae. *DNA Res.* **27**, dsaa016 (2020).
8. Rastogi, S. *et al.* Unravelling the genome of Holy basil: an "incomparable" "elixir of life" of traditional Indian medicine. *BMC Genomics* **16**, 413 (2015).
9. Upadhyay, A. K. *et al.* Genome sequencing of herb Tulsi (*Ocimum tenuiflorum*) unravels key genes behind its strong medicinal properties. *BMC Plant Biol.* **15**, 212 (2015).
10. Zhang, Y. *et al.* Incipient diploidization of the medicinal plant *Perilla* within 10,000 years. *Nat. Commun.* **12**, 5508 (2021).
11. He, Y. *et al.* Building an octaploid genome and transcriptome of the medicinal plant *Pogostemon cablin* from Lamiales. *Sci. Data* **5**, 180274 (2018).

12. He, Y. *et al.* Survey of the genome of *Pogostemon cablin* provides insights into its evolutionary history and sesquiterpenoid biosynthesis. *Sci. Rep.* **6**, 26405 (2016).

13. Shen, Y. *et al.* Chromosome-level and haplotype-resolved genome provides insight into the tetraploid hybrid origin of patchouli. *Nat. Commun.* **13**, 3511 (2022).

14. Zheng, X. *et al.* Insights into salvianolic acid B biosynthesis from chromosome-scale assembly of the *Salvia bowleyana* genome. *J. Integr. Plant Biol.* **63**, 1309–1323 (2021).

15. Xu, H. *et al.* Analysis of the genome sequence of the medicinal plant *Salvia miltiorrhiza*. *Mol. Plant* **9**, 949–952 (2016).

16. Zhang, G. *et al.* Hybrid de novo genome assembly of the Chinese herbal plant danshen (*Salvia miltiorrhiza* Bunge). *GigaScience* **4**, s13742-015-0104–3 (2015).

17. Song, Z. *et al.* A high-quality reference genome sequence of *Salvia miltiorrhiza* provides insights into tanshinone synthesis in its red rhizomes. *Plant Genome* **13**, e20041 (2020).

18. Ma, Y. *et al.* Expansion within the CYP71D subfamily drives the heterocyclization of tanshinones synthesis in *Salvia miltiorrhiza*. *Nat. Commun.* **12**, 685 (2021).

19. Dong, A.-X. *et al.* High-quality assembly of the reference genome for scarlet sage, *Salvia splendens*, an economically important ornamental plant. *GigaScience* **7**, giy068 (2018).

20. Jia, K.-H. *et al.* Chromosome-scale assembly and evolution of the tetraploid *Salvia splendens* (Lamiaceae) genome. *Hortic. Res.* **8**, 177 (2021).

21. Zhao, Q. *et al.* The reference genome sequence of *Scutellaria baicalensis* provides insights into the evolution of wogonin biosynthesis. *Mol. Plant* **12**, 935–950 (2019).

22. Xu, Z. *et al.* Comparative genome analysis of *Scutellaria baicalensis* and *Scutellaria barbata* reveals the evolution of active flavonoid biosynthesis. *Genomics Proteomics Bioinformatics* **18**, 230–240 (2020).

23. Yasodha, R. *et al.* Draft genome of a high value tropical timber tree, Teak (*Tectona grandis* L. f): Insights into SSR diversity, phylogeny and conservation. *DNA Res.* **25**, 409–419 (2018).

24. Zhao, D. *et al.* A chromosomal-scale genome assembly of *Tectona grandis* reveals the importance of tandem gene duplication and enables discovery of genes in natural product biosynthetic pathways. *GigaScience* **8**, (2019).

25. Sun, M. *et al.* Chromosome-level assembly and analysis of the *Thymus* genome provide insights on glandular secretory trichome formation and monoterpenoid biosynthesis in thyme. *Plant Commun.* 100413 (2022) doi:10.1016/j.xplc.2022.100413.

26. Isyaka, S. M. *et al.* Ent-abietane and ent-pimarane diterpenoids from *Croton mubango (Euphorbiaceae)*. *Phytochemistry* **170**, 112217 (2020).

27. Cahn-Ingold-Prelog Sterochemistry page. <https://chemapps.stolaf.edu/jmol/jsmol/cip.htm>. Accessed 4th April 2022.
