# Supplementary material for: Uncovering a miltiradiene biosynthetic gene cluster in the Lamiaceae reveals a dynamic evolutionary trajectory
Source: Nat Commun. 2023 Jan 20;14:343. doi: 10.1038/s41467-023-35845-1 (PMC9860074; doi:10.1038/s41467-023-35845-1)
Supplement: Supplementary file 3 — Reporting Summary [file 41467_2023_35845_MOESM3_ESM.pdf]

nature portfolio

Corresponding author(s): Bjoern Hamberger
Last updated by author(s): Dec 22, 2022

Reporting Summary

Nature Portfolio wishes to improve the reproducibility of the work that we publish. This form provides structure for consistency and transparency in reporting. For further information on Nature Portfolio policies, see our Editorial Policies and the Editorial Policy Checklist.

Statistics

For all statistical analyses, confirm that the following items are present in the figure legend, table legend, main text, or Methods section.

n/a	Confirmed	
☒	☐	The exact sample size (n) for each experimental group/condition, given as a discrete number and unit of measurement
☒	☐	A statement on whether measurements were taken from distinct samples or whether the same sample was measured repeatedly
☒	☐	The statistical test(s) used AND whether they are one- or two-sided<br>*Only common tests should be described solely by name; describe more complex techniques in the Methods section.*
☒	☐	A description of all covariates tested
☒	☐	A description of any assumptions or corrections, such as tests of normality and adjustment for multiple comparisons
☒	☐	A full description of the statistical parameters including central tendency (e.g. means) or other basic estimates (e.g. regression coefficient) AND variation (e.g. standard deviation) or associated estimates of uncertainty (e.g. confidence intervals)
☒	☐	For null hypothesis testing, the test statistic (e.g. F, t, r) with confidence intervals, effect sizes, degrees of freedom and P value noted<br>*Give P values as exact values whenever suitable.*
☒	☐	For Bayesian analysis, information on the choice of priors and Markov chain Monte Carlo settings
☒	☐	For hierarchical and complex designs, identification of the appropriate level for tests and full reporting of outcomes
☒	☐	Estimates of effect sizes (e.g. Cohen's d, Pearson's r), indicating how they were calculated

Our web collection on statistics for biologists contains articles on many of the points above.

Software and code

Policy information about availability of computer code

Data collection	10S Supernova (v.2.1.1)<br>Augustus (v.3.3)
Data analysis	BLAST (v.2.10.0)<br>MCScanX (v.1.0)<br>SynVisio (v1.0)<br>ClustalOmega (v.1.2.4)<br>Phytools R (v.0.7)<br>RAxML (v.8.2.12)<br>assembly-stats (v.1.0.1)<br>BUSCO (v.5.2.2)

For manuscripts utilizing custom algorithms or software that are central to the research but not yet described in published literature, software must be made available to editors and reviewers. We strongly encourage code deposition in a community repository (e.g. GitHub). See the Nature Portfolio guidelines for submitting code & software for further information.

Data

Policy information about availability of data

All manuscripts must include a data availability statement. This statement should provide the following information, where applicable:

- Accession codes, unique identifiers, or web links for publicly available datasets
- A description of any restrictions on data availability
- For clinical datasets or third party data, please ensure that the statement adheres to our policy

The data supporting the findings of this work are available within the paper and the Supplementary Information and Data files. The raw genomic reads generated in this study have been deposited in the NCBI BioSample database under the following accession codes Plectranthus barbatus (SAMN26547115), Leonotis leonurus (SAMN26547116), and Prunella vulgaris (SAMN26547117). The genome assemblies have been deposited in NCBI with accession codes Plectranthus barbatus (JAPKLW000000000), Leonotis leonurus (JAPKLX000000000), and Prunella vulgaris (JAPKLY000000000). The versions described in this paper are versions XXXXXX010000000. Sequences for the functionally characterized enzymes from Callicarpa americana can be found in the NCBI GenBank database: ON260868-ON260876. Data used to prepare figures is given as Source Data file. Additional Supplementary materials including genome assemblies and annotations, phylogenetic alignments, cluster sequences, and collinearity files can be found in our Dryad Repository: https://doi.org/10.5061/dryad.w9ghx3frg.130

Human research participants

Policy information about studies involving human research participants and Sex and Gender in Research.

Reporting on sex and gender	*Use the terms sex (biological attribute) and gender (shaped by social and cultural circumstances) carefully in order to avoid confusing both terms. Indicate if findings apply to only one sex or gender; describe whether sex and gender were considered in study design whether sex and/or gender was determined based on self-reporting or assigned and methods used. Provide in the source data disaggregated sex and gender data where this information has been collected, and consent has been obtained for sharing of individual-level data; provide overall numbers in this Reporting Summary. Please state if this information has not been collected. Report sex- and gender-based analyses where performed, justify reasons for lack of sex- and gender-based analysis.*
Population characteristics	*Describe the covariate-relevant population characteristics of the human research participants (e.g. age, genotypic information, past and current diagnosis and treatment categories). If you filled out the behavioural & social sciences study design questions and have nothing to add here, write "See above."*
Recruitment	*Describe how participants were recruited. Outline any potential self-selection bias or other biases that may be present and how these are likely to impact results.*
Ethics oversight	*Identify the organization(s) that approved the study protocol.*

Note that full information on the approval of the study protocol must also be provided in the manuscript.

Field-specific reporting

Please select the one below that is the best fit for your research. If you are not sure, read the appropriate sections before making your selection.

☒ Life sciences ☐ Behavioural & social sciences ☐ Ecological, evolutionary & environmental sciences

For a reference copy of the document with all sections, see nature.com/documents/nr-reporting-summary-flat.pdf

Life sciences study design

All studies must disclose on these points even when the disclosure is negative.

Sample size	All samples, i.e., members of the mint family with an identified published genome were considered for this study, including three de novo sequenced and assembled genomes of Prunella, Plectranthus and Leonotis (this work). Hence, the species investigated are based on data availability, assembly quality/coverage, and distribution throughout the Lamiaceae family.
Data exclusions	The genomes of Prunella vulgaris, Plectranthus barbatus, and R. officinalis were too fragmented to determine whether they were part of a larger cluster. They were used to confirm presence of the target TPS genes. Thus determination did not use pre-established criteria but was pragmatic. In brief, we detected the relevant genes in the genomes, but at the ends of smaller contigs. It is plausible that the BGSs exist, but without further genome sequencing we prefer not to speculate.<br>In detail: R. officinalis: This genome assembly has a relatively low coverage, of approximately 85%. We identified a scaffold that includes collinear regions to those surrounding, but not including those in the BGC in C. americana. When we searched the genome specifically for CamTPS9 orthologs (using the same >70% cutoff as the rest of the analysis), we found two candidates. Unfortunately, they were on very small scaffolds containing three and four genes, respectively. Taken together, we cannot confidently say that this species lacks this cluster but advise a more complete sequencing effort before any conclusions can be made.<br>P. vulgaris: This is a relatively fragmented assembly so drawing conclusions from genomic structure must be done cautiously. We found 3

physically clustered genes which share synteny with the BGC in C. americana; these 3 genes were on the end of a scaffold, suggesting the cluster may have been split during sequencing. Additionally, these genes were orthologous to a class-I TPS, a CYP76, and a CYP71 -- therefore these three alone do not constitute a full terpenoid pathway since it is at least missing a Class-II gene. The CamTPS9 ortholog(s) we identified with MCScanX as syntenic to the C. americana BGC were on the end of a small scaffold containing only 38 genes. Therefore, it is plausible that this cluster is present and whole but was split during sequencing; however in order to determine the presence or absence of this cluster more sequencing is required. Interestingly, the published miltiradiene synthase from P. vulgaris is not the same as those identified as syntenic by MCScanX, suggesting that perhaps this plant has multiple copies of this gene.
P. barbatus: Once again, highly congruous genomes seem to be crucial for mapping lengthy synteny. A miltiradiene synthase has been found and characterized in P. barbatus, but no genes were identified as syntenic according to my MCScanX analysis. However, we hesitate to conclude that this means the cluster is missing and suggest that a more contiguous assembly is necessary to determine cluster presence or absence.

Replication

Phylogenetic support was provided by maximum likelihood by RAxML (version 8.2.12)112 with support from 1000 bootstrap replicates. All transient expression experiments were performed in triplicates. All attempts at replication were successful. PCR, cloning and constructs were verified with genomic models and the transcriptomic data and fully consistent. Transcriptomic data was mapped to the corresponding genome sequences and verified. This information was used to build the gene models, which were for all cloned genes verified through Sanger sequencing.

Randomization

This study is an exploratory analysis of genomes of the mint family. All identified and available data was used in this study. As this study does not include differential treatments, or differential assessment of outcomes, randomization was not applicable. All experimental samples were treated equally, with standardized methods described in the main text. This ensured that the outcomes measured were as objective as possible. This was confirmed, where applicable, through replication. Conclusions drawn from biochemical analysis have been reproduced at least 3x each and are reproducible given the conditions and primers presented in the methods/supplemental. Conclusions drawn from genomic data are reproducible from publicly available genome assemblies and the given parameters of each software.

Blinding

This study is an exploratory analysis of genomes of the mint family. Blinding is not applicable for genome sequencing. For all heterologous expression of the recombinant enzymes standardized analytical procedures were applied, including validation of the results through replication.

Reporting for specific materials, systems and methods

We require information from authors about some types of materials, experimental systems and methods used in many studies. Here, indicate whether each material, system or method listed is relevant to your study. If you are not sure if a list item applies to your research, read the appropriate section before selecting a response.

Materials & experimental systems

n/a	Involved in the study	
☒	☐	Antibodies
☒	☐	Eukaryotic cell lines
☒	☐	Palaeontology and archaeology
☒	☐	Animals and other organisms
☒	☐	Clinical data
☒	☐	Dual use research of concern

Methods

n/a	Involved in the study	
☒	☐	ChIP-seq
☒	☐	Flow cytometry
☒	☐	MRI-based neuroimaging
