# Supplementary material for: Uncovering a miltiradiene biosynthetic gene cluster in the Lamiaceae reveals a dynamic evolutionary trajectory
Source: Nat Commun. 2023 Jan 20;14:343. doi: 10.1038/s41467-023-35845-1 (PMC9860074; doi:10.1038/s41467-023-35845-1)
Supplement: Supplementary file 4 — Source Data [file 41467_2023_35845_MOESM4_ESM.zip › Fig. 5 and Supplementary Fig. 6 Source Data.docx]

Datasets used in the creation of Fig. 5 (*C. americana* only) and Supplementary Fig. 6

| **Species** | **SRR identifier** | **Tissue Type** |
| --- | --- | --- |
| *Callicarpa americana* | SRR8927023 | open flower |
|  | SRR8927024 | root |
|  | SRR8927025 | fruit |
|  | SRR8927026 | closed flower |
|  | SRR8927027 | young leaf |
|  | SRR8927028 | mature leaf |
|  | SRR8927029 | petiole |
|  | SRR8927030 | stem |
| *Hyssopus officinalis* | SRR8931120 | mature leaf |
|  | SRR8931118 | stem |
|  | SRR8931117 | young leaf |
|  | SRR8931115 | petiole |
|  | SRR8931116 | root |
|  | SRR8931114 | closed flower |
|  | SRR8931113 | open flower |
| *Leonotis leonurus* | SRR5150731 | leaf |
| *Pogostemon cablin* | SRR13949260 | young leaf |
|  | SRR13949262 | young stem |
|  | SRR13949274 | young root |
|  | SRR13949268 | seedling |
|  | SRR8769985 | flower |
|  | SRR8820009 | leaf with high light |
|  | SRR8809557 | leaf with low light |
|  | SRR8793583 | leaf with no light |
|  | SRR8775238 | leaf with ethylene |
|  | SRR8767851 | leaf with abscisic acid |
|  | SRR8835225 | leaf with methyl jasmonate |
| *Salvia miltiorrhiza* | SRR17674928 | open flower |
|  | SRR13861127 | root |
|  | SRR11126657 | stem |
|  | SRR9678069 | roots with UVB |
|  | SRR1640458 | velamen, cortex and xylem |
|  | SRR1045051 | leaf |
|  | SRR946950 | leaf with methyl jasmonate |
|  | SRR19500593 | leaf with abscisic acid |
|  | SRR19500602 | root with abscisic acid |
| *Scutellaria baicalensis* | SRR8449931 | root |
|  | SRR8449932 | leaf |
|  | SRR8449935 | stem |
|  | SRR7689123 | flowers, flowers buds, leaves, root, and stems with methyl jasmonate |
|  | SRR7665600 | flowers, flowers buds, leaves, root, and stems |
| *Tectona grandis* | SRR4017280 | random sample of total transcriptome |
|  | SRR4017281 | random sample of total transcriptome after drought treatment |
|  | SRR2080156 | seedling |
|  | SRR2080154 | leaf |
|  | SRR2080155 | root |
|  | SRR2080153 | flower |
