# Supplementary material for: Uncovering a miltiradiene biosynthetic gene cluster in the Lamiaceae reveals a dynamic evolutionary trajectory
Source: Nat Commun. 2023 Jan 20;14:343. doi: 10.1038/s41467-023-35845-1 (PMC9860074; doi:10.1038/s41467-023-35845-1)
Supplement: Supplementary file 4 — Source Data [file 41467_2023_35845_MOESM4_ESM.zip › Figs. 6 and 7, Supplementary Figs. 7, 11, 12, 16, 20 Source Data Inventory of raw GC_MS data.docx]

Inventory of GC/MS raw data files

GCMS.tar.gz

Fig6 _Supplementary Figs. 7, 11_Caam_diTPSs/

(hexane extracts)

A_20220321_hex_EL01.CDF | DXS + GGPPS control

A_20220321_hex_EL03.CDF | CamTPS1

A_20220321_hex_EL04.CDF | CamTPS1 + NmTPS2

A_20220321_hex_EL05.CDF | CamTPS1 + CamTPS12

A_20220321_hex_EL06.CDF | CamTPS1 + CamTPS10

A_20220321_hex_EL07.CDF | CamTPS6

A_20220321_hex_EL08.CDF | CamTPS6 + PbTPS3

A_20220321_hex_EL09.CDF | CamTPS6 + CamTPS9

A_20220321_hex_EL10.CDF | CamTPS6 + CamTPS10

A_20220321_hex_EL11.CDF | CamTPS6 + CamTPS12

A_20220321_hex_EL13.CDF | CamTPS7

A_20220321_hex_EL14.CDF | CamTPS7 + CamTPS10

A_20220321_hex_EL15.CDF | CamTPS7 + CamTPS9

Fig7a_Supplementary Fig. 12 CYP76AHs/

(ethyl acetate extracts)

A_211015_004.CDF | DXS + GGPPS control

A_211015_008.CDF | CamTPS6 + CamTPS9

A_211015_017.CDF | CamTPS6 + CamTPS9 + CamCYP76AH67

A_211015_020.CDF | CamTPS6 + CamTPS9 + CamCYP76AH67 + CamCYP76AH68

A_211015_024.CDF | CamTPS6 + CamTPS9 + CamCYP76AH68

Fig7b_Supplementary Fig. 16_CYP71Ds/

(ethyl acetate extracts)

A_20220128_002.CDF | DXS + GGPPS control

A_20220128_005.CDF | CamTPS6

A_20220128_006.CDF | CamTPS6 + SsSS

A_20220128_010.CDF | CamCYP71D717 + CamCYP71D716

A_20220128_015.CDF | CamTPS6 + CamCYP71D716

A_20220128_016.CDF | CamTPS6 + CamCYP71D717

A_20220128_017.CDF | CamTPS6 + CamCYP71D716 + CamCYP71D717

A_20220128_018.CDF | CamTPS6 + SsSS + CamCYP71D716

Supplementary Fig. 20_Caamroot/

A_20220721_EL35.CDF| Caam root extract, ethyl acetate
