# Supplementary material for: Uncovering a miltiradiene biosynthetic gene cluster in the Lamiaceae reveals a dynamic evolutionary trajectory
Source: Nat Commun. 2023 Jan 20;14:343. doi: 10.1038/s41467-023-35845-1 (PMC9860074; doi:10.1038/s41467-023-35845-1)
Supplement: Supplementary file 4 — Source Data [file 41467_2023_35845_MOESM4_ESM.zip › Supplementary Fig. 12 Source Data 2.pdf]

Unknown; InLib=-496

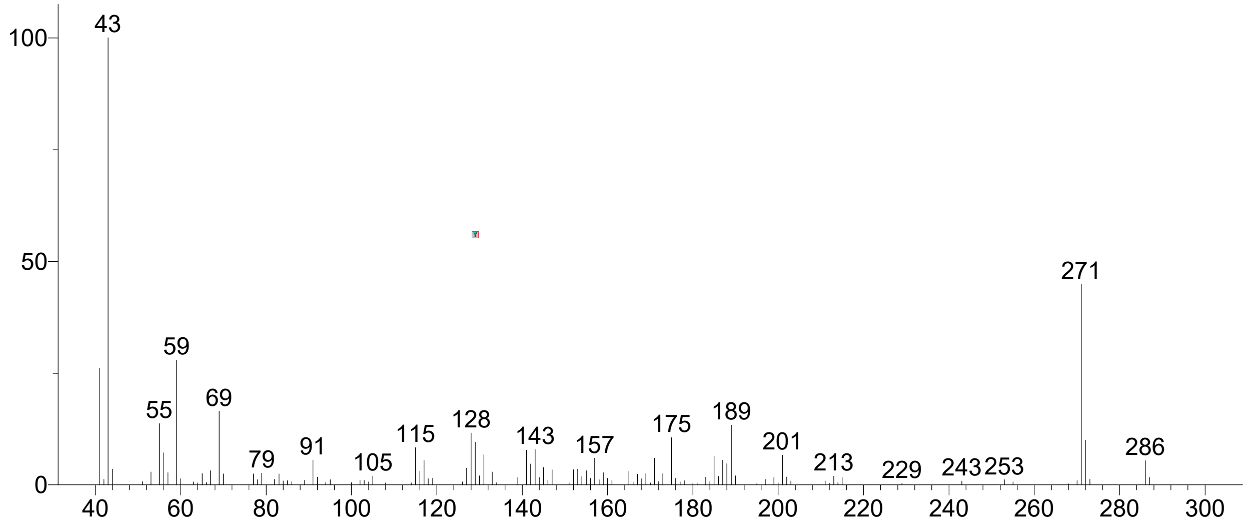


(Text File) Component at scan 2264 (13.660 min) [Model = +43u] in X:\ABBY GCMS\20211015\A_211015_017.D\DATA.MS

Name: Component at scan 2264 (13.660 min) [Model = +43u] in X:\ABBY GCMS\20211015\A_211015_017.D\DATA.
MS
MW: N/A ID#: 1015 DB: Text File
10 largest peaks:

| | | | | | | | | | |
|---|---|---|---|---|---|---|---|---|---|
| 43 999 | 271 448 | 59 279 | 41 260 | 69 165 | 55 138 | 189 134 | 128 116 | 175 106 | 272 99 |

116 m/z Values and Intensities:

| | | | | | | | | | |
|---|---|---|---|---|---|---|---|---|---|
| 41 260 | 42 12 | 43 999 | 44 35 | 51 7 | 53 28 | 55 138 | 56 71 | 57 27 | 59 279 |
| 60 13 | 63 6 | 64 4 | 65 25 | 66 5 | 67 31 | 69 165 | 70 24 | 77 24 | 78 11 |
| 79 27 | 82 12 | 83 24 | 84 8 | 85 9 | 86 7 | 89 10 | 91 56 | 92 17 | 94 5 |
| 95 11 | 100 5 | 102 10 | 103 10 | 104 6 | 105 20 | 108 4 | 114 4 | 115 84 | 116 30 |
| 117 54 | 118 13 | 119 14 | 126 6 | 127 37 | 128 116 | 129 95 | 130 20 | 131 67 | 133 28 |
| 134 5 | 139 16 | 141 77 | 142 46 | 143 79 | 144 16 | 145 38 | 146 10 | 147 33 | 151 5 |
| 152 33 | 153 35 | 154 18 | 155 31 | 156 14 | 157 60 | 158 11 | 159 27 | 160 14 | 161 10 |
| 165 30 | 166 6 | 167 23 | 168 13 | 169 25 | 170 4 | 171 59 | 172 7 | 173 25 | 175 106 |
| 176 14 | 177 6 | 178 9 | 180 3 | 181 4 | 183 17 | 184 7 | 185 63 | 186 18 | 187 55 |
| 188 47 | 189 134 | 190 20 | 195 3 | 197 12 | 199 16 | 200 5 | 201 67 | 202 17 | 203 8 |
| 211 8 | 212 3 | 213 20 | 214 5 | 215 16 | 229 4 | 243 9 | 253 12 | 255 6 | 268 2 |
| 270 9 | 271 448 | 272 99 | 273 12 | 286 55 | 287 16 | | | | |

Synonyms:
no synonyms.

Hit 1 : 2-Phenanthrenol, 4b,5,6,7,8,8a,9,10-octahydro-4b,8,8-trimethyl-1-(1-methylethyl)-, (4bS-trans)-
C20H30O; MF: 753; RMF: 775; Prob 61.2%; CAS: 511-15-9; Lib: mainlib; ID: 238936.

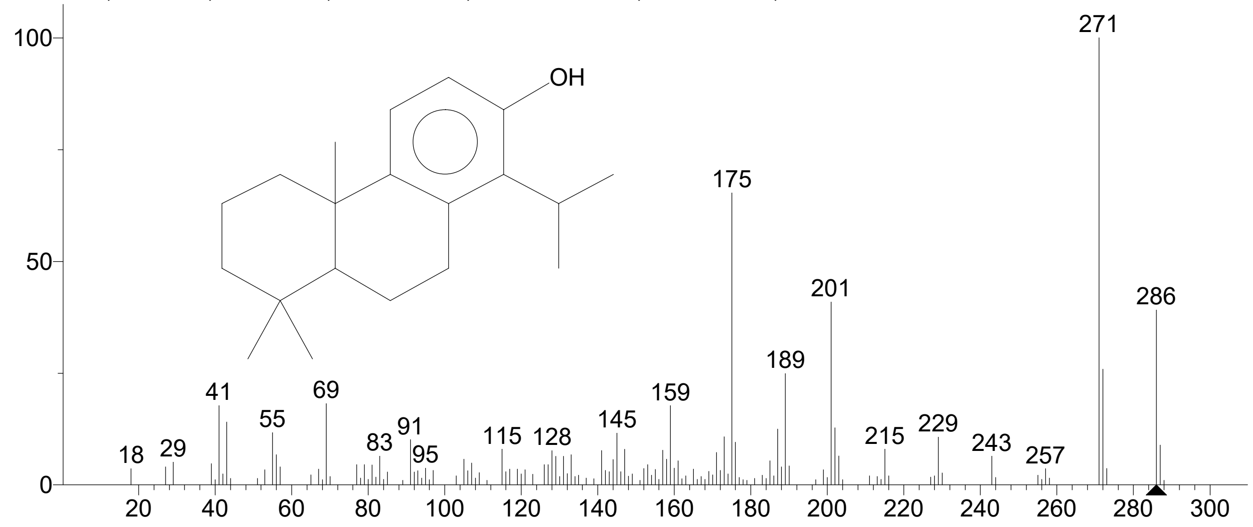


(mainlib) 2-Phenanthrenol, 4b,5,6,7,8,8a,9,10-octahydro-4b,8,8-trimethyl-1-(1-methylethyl)-, (4bS-trans)-

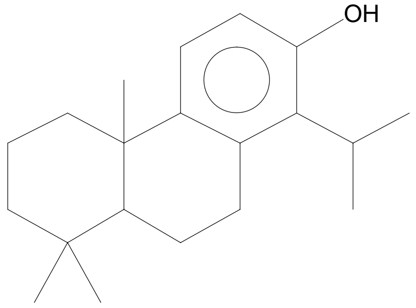


Name: 2-Phenanthrenol, 4b,5,6,7,8,8a,9,10-octahydro-4b,8,8-trimethyl-1-(1-methylethyl)-, (4bS-trans)-
Formula: $C_{20}H_{30}O$
MW: 286 Exact Mass: 286.229666 CAS#: 511-15-9 NIST#: 42592 ID#: 238936 DB: mainlib
Other DBs: NIH
Contributor: R RYHAGE MS-LAB KAROLINSKA INSTITUTET STOCKHOLM SWEDEN
InChIKey: ZRVDANDJSTYELM-UHFFFAOYSA-N Non-stereo
10 largest peaks:

| | | | | | | | | | |
|---|---|---|---|---|---|---|---|---|---|
| 271 999 | 175 653 | 201 409 | 286 391 | 272 258 | 189 249 | 69 182 | 41 178 | 159 177 | 43 140 |

136 m/z Values and Intensities:

| | | | | | | | | | |
|---|---|---|---|---|---|---|---|---|---|
| 18 37 | 27 40 | 29 51 | 39 47 | 40 11 | 41 178 | 42 24 | 43 140 | 44 14 | 51 14 |
| 53 33 | 55 117 | 56 67 | 57 40 | 65 22 | 67 35 | 68 11 | 69 182 | 70 18 | 77 45 |
| 78 15 | 79 45 | 80 12 | 81 44 | 82 17 | 83 65 | 84 12 | 85 28 | 89 10 | 91 101 |
| 92 28 | 93 31 | 94 15 | 95 38 | 96 11 | 97 32 | 103 20 | 105 57 | 106 31 | 107 48 |
| 108 15 | 109 27 | 111 10 | 115 80 | 116 29 | 117 35 | 119 35 | 120 24 | 121 33 | 123 23 |
| 126 45 | 127 45 | 128 77 | 129 63 | 130 18 | 131 63 | 132 25 | 133 67 | 134 18 | 135 21 |
| 137 15 | 139 13 | 141 76 | 142 32 | 143 29 | 144 56 | 145 116 | 146 29 | 147 79 | 148 19 |

| | | | | | | | | | |
|---|---|---|---|---|---|---|---|---|---|
| 149 24 | 151 10 | 152 36 | 153 45 | 154 21 | 155 34 | 156 12 | 157 77 | 158 57 | 159 177 |
| 160 37 | 161 53 | 162 13 | 163 20 | 165 35 | 166 12 | 167 18 | 168 12 | 169 30 | 170 22 |
| 171 72 | 172 32 | 173 107 | 174 24 | 175 653 | 176 95 | 177 16 | 178 11 | 179 10 | 181 14 |
| 183 21 | 184 14 | 185 53 | 186 20 | 187 124 | 188 40 | 189 249 | 190 42 | 197 11 | 199 33 |
| 200 16 | 201 409 | 202 127 | 203 64 | 204 11 | 211 20 | 213 18 | 214 12 | 215 80 | 216 20 |
| 227 17 | 228 20 | 229 107 | 230 26 | 243 64 | 244 16 | 255 21 | 256 12 | 257 36 | 258 15 |
| 271 999 | 272 258 | 273 36 | 286 391 | 287 88 | 288 10 | | | | |

Synonyms:
1.Podocarpa-8,11,13-trien-13-ol, 14-isopropyl-
2.Totarol
3.TOTAROL (B637832K176)
4.(+)-Totarol
5.14-Isopropylpodocarpa-8,11,13-trien-13-ol #
6.trans-Totarol

Experimental RI median±deviation (#data)
Semi-standard non-polar:2302±0 (14)
Standard non-polar: 2268±15 (7)

Hit 2 : 13-Isopropylpodocarpen-12-ol-20-al
C20H28O2; MF: 712; RMF: 746; Prob 13.9%; CAS: 24035-37-8; Lib: replib; ID: 37061.

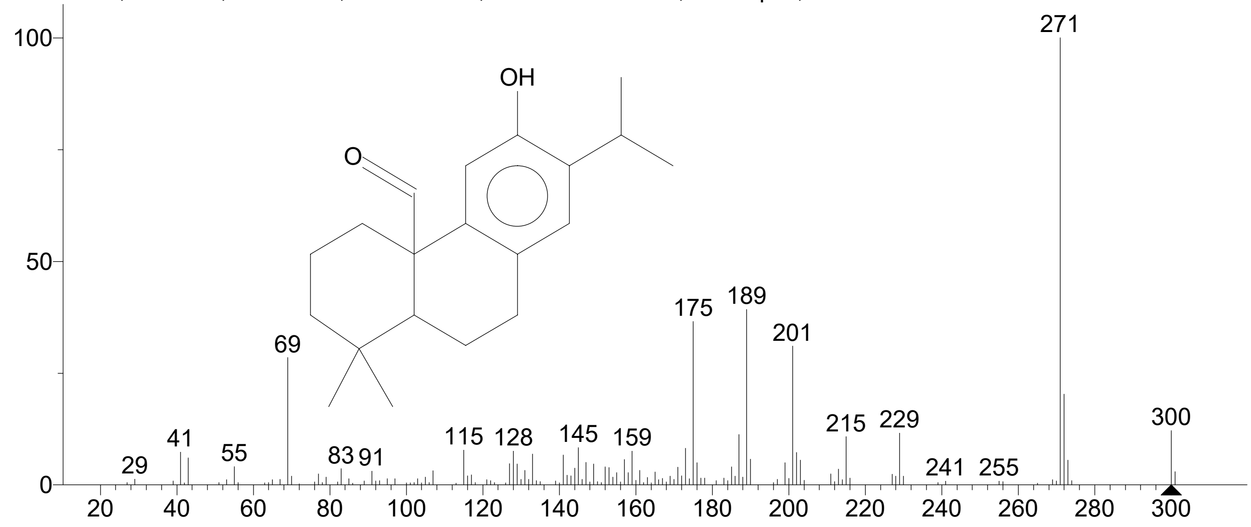


(replib) 13-Isopropylpodocarpen-12-ol-20-al

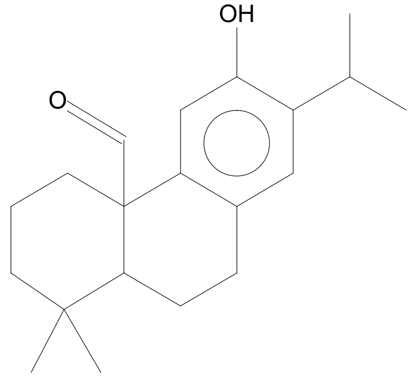


Name: 13-Isopropylpodocarpen-12-ol-20-al
Formula: $C_{20}H_{28}O_2$
MW: 300 Exact Mass: 300.208931 CAS#: 24035-37-8 NIST#: 414191 ID#: 37061 DB: replib
Other DBs: None
Contributor: NIST Mass Spectrometry Data Center
InChIKey: YPWYNONCSGZEQQ-UHFFFAOYSA-N   Non-stereo
10 largest peaks:

| | | | | | | | | | |
|---|---|---|---|---|---|---|---|---|---|
| 271 999 | 189 392 | 175 365 | 201 310 | 69 284 | 272 202 | 300 122 | 229 116 | 187 112 | 215 108 |

137 m/z Values and Intensities:

| | | | | | | | | | |
|---|---|---|---|---|---|---|---|---|---|
| 27 5 | 29 13 | 39 8 | 41 73 | 42 4 | 43 60 | 51 5 | 53 11 | 55 41 | 56 5 |
| 63 4 | 64 5 | 65 11 | 67 12 | 69 284 | 70 19 | 72 2 | 76 6 | 77 24 | 78 5 |
| 79 17 | 82 6 | 83 36 | 85 13 | 86 3 | 89 8 | 91 31 | 92 8 | 93 9 | 95 13 |
| 97 13 | 100 4 | 101 4 | 102 5 | 103 13 | 104 4 | 105 17 | 106 5 | 107 31 | 113 3 |
| 115 78 | 116 20 | 117 22 | 118 5 | 121 11 | 122 9 | 123 5 | 126 4 | 127 47 | 128 76 |
| 129 46 | 130 12 | 131 32 | 132 12 | 133 68 | 134 9 | 135 7 | 139 8 | 140 4 | 141 66 |
| 142 21 | 143 20 | 144 37 | 145 84 | 146 12 | 147 50 | 148 6 | 149 46 | 150 7 | 151 5 |

| | | | | | | | | | |
|---|---|---|---|---|---|---|---|---|---|
| 152 40 | 153 38 | 154 17 | 155 27 | 156 6 | 157 56 | 158 27 | 159 76 | 160 10 | 161 32 |
| 162 5 | 163 16 | 164 4 | 165 28 | 166 11 | 167 14 | 168 6 | 169 19 | 170 12 | 171 39 |
| 172 20 | 173 81 | 174 13 | 175 365 | 176 49 | 177 15 | 178 15 | 181 9 | 183 15 | 184 9 |
| 185 40 | 186 19 | 187 112 | 188 17 | 189 392 | 190 57 | 196 5 | 197 12 | 199 49 | 200 14 |
| 201 310 | 202 72 | 203 55 | 204 10 | 211 24 | 212 7 | 213 35 | 214 11 | 215 108 | 216 15 |
| 227 23 | 228 19 | 229 116 | 230 19 | 239 5 | 241 9 | 255 9 | 256 7 | 265 3 | 269 11 |
| 270 8 | 271 999 | 272 202 | 273 55 | 274 9 | 300 122 | 301 29 | | | |

Synonyms:
1.12-Hydroxyabieta-9(11),8(14),12-trien-20-al #

Experimental RI median±deviation (#data)
Semi-standard non-polar:2569±N/A (1)
Standard non-polar: 2502±0 (2)

Hit 3 : 13-Isopropylpodocarpen-12-ol-20-al
C20H28O2; MF: 710; RMF: 753; Prob 13.9%; CAS: 24035-37-8; Lib: mainlib; ID: 238951.

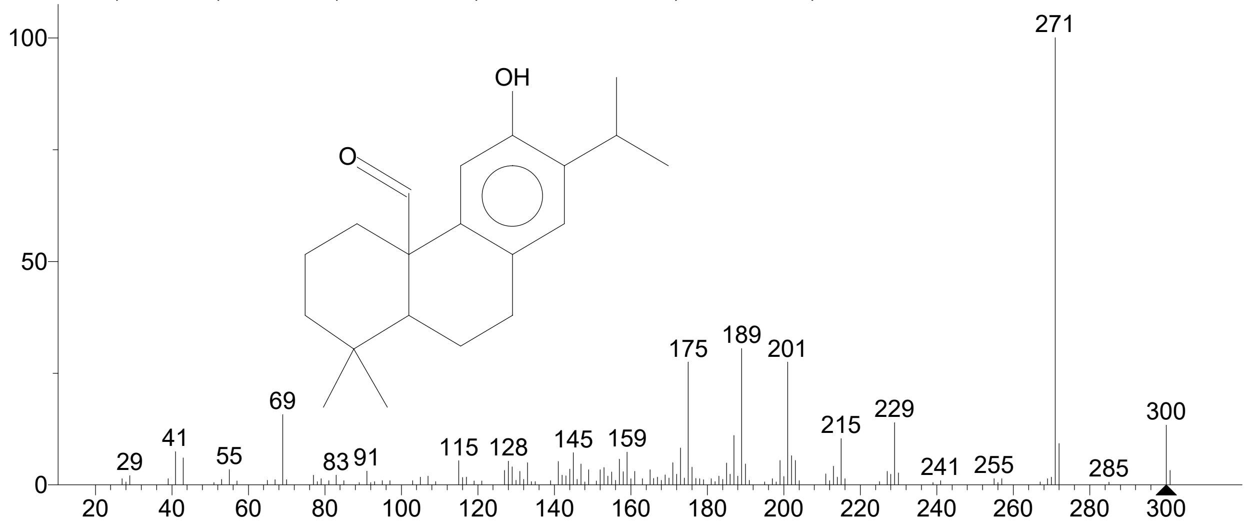


(mainlib) 13-Isopropylpodocarpen-12-ol-20-al

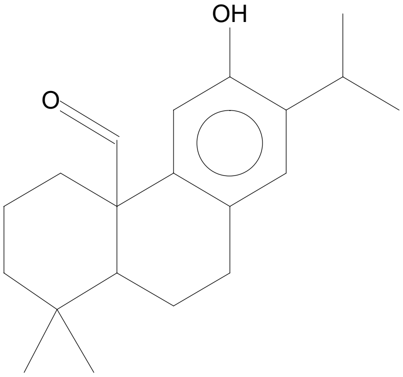


Name: 13-Isopropylpodocarpen-12-ol-20-al
Formula: $C_{20}H_{28}O_2$
MW: 300 Exact Mass: 300.208931 CAS#: 24035-37-8 NIST#: 42992 ID#: 238951 DB: mainlib
Other DBs: None
Contributor: R RYHAGE MS-LAB KAROLINSKA INSTITUTET STOCKHOLM SWEDEN
InChIKey: YPWYNONCSGZEQQ-UHFFFAOYSA-N Non-stereo
10 largest peaks:

| | | | | | | | | | |
|---|---|---|---|---|---|---|---|---|---|
| 271 999 | 189 305 | 175 274 | 201 274 | 69 158 | 229 140 | 300 134 | 187 110 | 215 104 | 272 92 |

125 m/z Values and Intensities:

| | | | | | | | | | |
|---|---|---|---|---|---|---|---|---|---|
| 27 13 | 28 6 | 29 21 | 39 13 | 41 75 | 43 60 | 51 5 | 53 12 | 55 35 | 57 8 |
| 65 10 | 67 11 | 69 158 | 70 11 | 77 21 | 78 7 | 79 13 | 81 9 | 83 22 | 85 9 |
| 89 5 | 91 31 | 92 5 | 93 7 | 95 9 | 97 9 | 103 9 | 105 17 | 107 19 | 109 7 |
| 115 54 | 116 16 | 117 17 | 119 8 | 121 8 | 127 32 | 128 53 | 129 40 | 130 10 | 131 30 |
| 132 12 | 133 49 | 134 7 | 135 7 | 139 9 | 141 52 | 142 21 | 143 20 | 144 35 | 145 72 |
| 146 12 | 147 46 | 148 6 | 149 33 | 151 8 | 152 33 | 153 38 | 154 19 | 155 28 | 156 10 |
| 157 57 | 158 29 | 159 74 | 160 13 | 161 30 | 163 13 | 165 33 | 166 14 | 167 17 | 168 10 |

| | | | | | | | | | |
|---|---|---|---|---|---|---|---|---|---|
| 169 22 | 170 15 | 171 49 | 172 23 | 173 82 | 174 15 | 175 274 | 176 39 | 177 14 | 178 13 |
| 179 11 | 181 13 | 182 7 | 183 19 | 184 12 | 185 48 | 186 23 | 187 110 | 188 19 | 189 305 |
| 190 46 | 191 10 | 195 6 | 197 13 | 198 6 | 199 54 | 200 18 | 201 274 | 202 65 | 203 54 |
| 204 9 | 211 24 | 212 9 | 213 41 | 214 17 | 215 104 | 216 13 | 225 7 | 227 30 | 228 23 |
| 229 140 | 230 26 | 239 5 | 241 10 | 255 15 | 256 5 | 257 14 | 267 6 | 269 13 | 270 17 |
| 271 999 | 272 92 | 285 7 | 300 134 | 301 32 | | | | | |

Synonyms:

1.12-Hydroxyabieta-9(11),8(14),12-trien-20-al #

Experimental RI median±deviation (#data)

Semi-standard non-polar:2569±N/A (1)

Standard non-polar: 2502±0 (2)

Hit 4 : Ferruginol
C20H30O; MF: 699; RMF: 706; Prob 9.01%; CAS: 514-62-5; Lib: mainlib; ID: 243935.

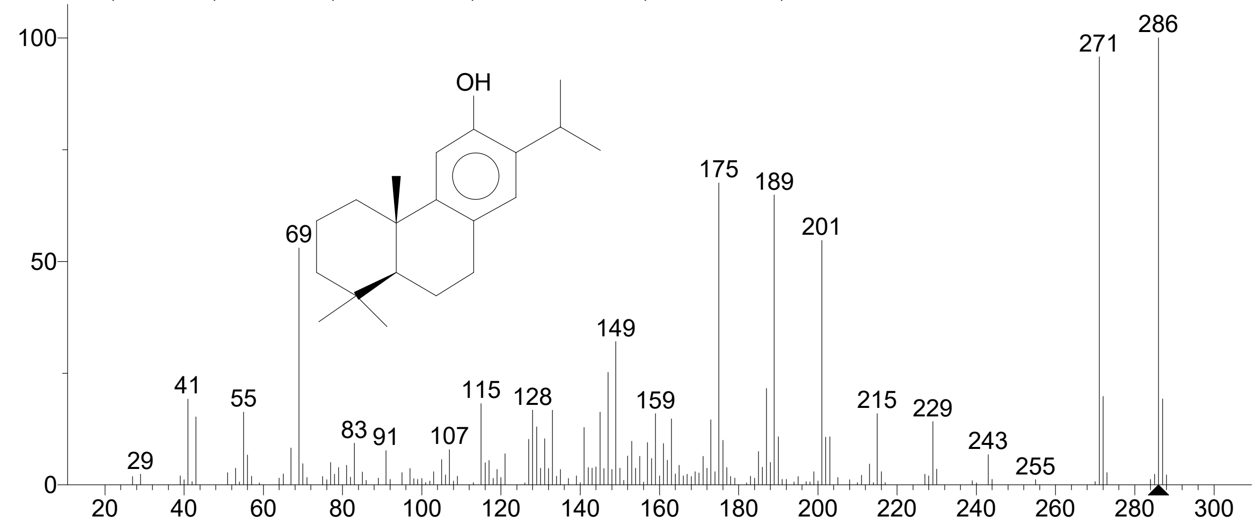


(mainlib) Ferruginol

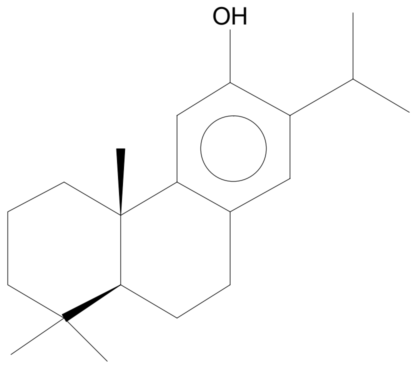


Name: Ferruginol
Formula: $C_{20}H_{30}O$
MW: 286 Exact Mass: 286.229666 CAS#: 514-62-5 NIST#: 413241 ID#: 243935 DB: mainlib
Other DBs: None
Contributor: NIST Mass Spectrometry Data Center
InChIKey: QXNWVJOHUAQHLM-AZUAARDMSA-N Non-stereo
10 largest peaks:

| | | | | | | | | | | | | | | | | | | | |
|---|---|---|---|---|---|---|---|---|---|---|---|---|---|---|---|---|---|---|---|
| 286 | 999 | 271 | 956 | 175 | 675 | 189 | 648 | 201 | 546 | 69 | 530 | 149 | 320 | 147 | 251 | 187 | 215 | 272 | 197 |

153 m/z Values and Intensities:

| | | | | | | | | | | | | | | | | | | | |
|---|---|---|---|---|---|---|---|---|---|---|---|---|---|---|---|---|---|---|---|
| 27 | 18 | 29 | 24 | 39 | 20 | 40 | 11 | 41 | 192 | 42 | 7 | 43 | 151 | 51 | 27 | 53 | 37 | 54 | 6 |
| 55 | 163 | 56 | 66 | 57 | 19 | 59 | 4 | 64 | 15 | 65 | 24 | 67 | 82 | 69 | 530 | 70 | 47 | 71 | 16 |
| 75 | 18 | 76 | 11 | 77 | 50 | 78 | 23 | 79 | 38 | 81 | 43 | 82 | 17 | 83 | 93 | 85 | 28 | 86 | 10 |
| 89 | 15 | 91 | 77 | 92 | 12 | 95 | 27 | 97 | 36 | 98 | 13 | 99 | 12 | 100 | 14 | 101 | 5 | 102 | 8 |
| 103 | 29 | 105 | 56 | 106 | 22 | 107 | 79 | 108 | 8 | 109 | 19 | 113 | 5 | 115 | 182 | 116 | 49 | 117 | 54 |
| 118 | 14 | 119 | 34 | 120 | 16 | 121 | 69 | 126 | 4 | 127 | 101 | 128 | 167 | 129 | 129 | 130 | 37 | 131 | 103 |
| 132 | 36 | 133 | 166 | 134 | 19 | 135 | 34 | 137 | 14 | 139 | 20 | 140 | 5 | 141 | 128 | 142 | 38 | 143 | 37 |

144 40 | 145 162 | 146 36 | 147 251 | 148 34 | 149 320 | 150 37 | 151 10 | 152 64 | 153 97 |
154 37 | 155 63 | 156 6 | 157 94 | 158 58 | 159 159 | 160 20 | 161 92 | 162 55 | 163 147 |
164 24 | 165 43 | 166 20 | 167 23 | 168 18 | 169 29 | 170 26 | 171 63 | 172 37 | 173 145 |
174 29 | 175 675 | 176 99 | 177 38 | 178 18 | 179 15 | 182 4 | 183 19 | 184 15 | 185 74 |
186 39 | 187 215 | 188 50 | 189 648 | 190 107 | 191 12 | 192 12 | 194 6 | 195 18 | 197 7 |
198 6 | 199 29 | 200 8 | 201 546 | 202 106 | 203 107 | 205 16 | 208 11 | 210 5 | 211 21 |
213 46 | 214 5 | 215 160 | 216 29 | 217 5 | 227 23 | 228 20 | 229 141 | 230 35 | 239 9 |
240 4 | 243 68 | 244 12 | 255 12 | 270 7 | 271 956 | 272 197 | 273 27 | 284 12 | 285 23 |
286 999 | 287 191 | 288 22 |

Synonyms:
1.Abieta-9(11),8(14),12-trien-12-ol #
2.trans-Ferruginol
3.Podocarpa-8,11,13-trien-12-ol, 13-isopropyl-

Experimental RI median±deviation (#data)
Semi-standard non-polar:2325±0 (16)
Standard non-polar: 2283±N/A (1)
Polar: 2295±N/A (1)

Hit 5 : Pisiferol
C20H30O2; MF: 684; RMF: 733; Prob 5.46%; CAS: 24035-36-7; Lib: mainlib; ID: 238950.

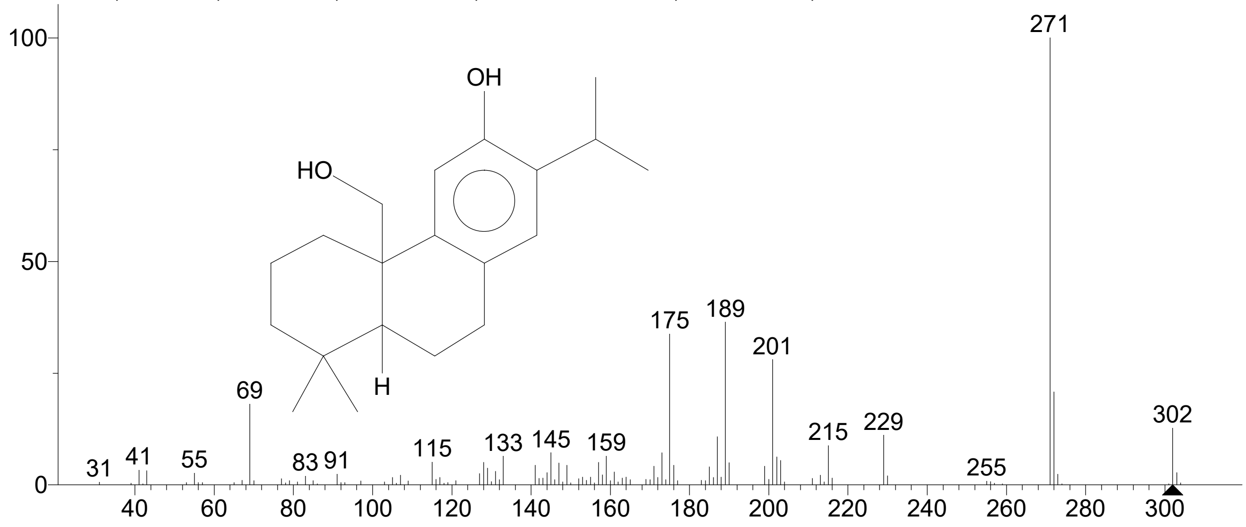


(mainlib) Pisiferol

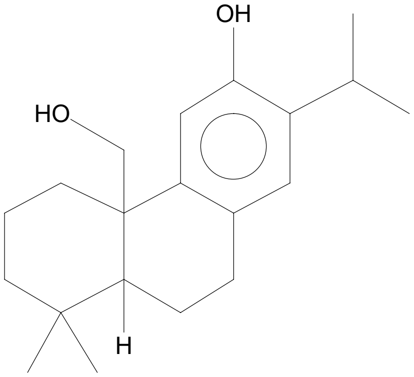


Name: Pisiferol
Formula: $C_{20}H_{30}O_2$
MW: 302 Exact Mass: 302.22458 CAS#: 24035-36-7 NIST#: 414875 ID#: 238950 DB: mainlib
Other DBs: None
Contributor: NIST Mass Spectrometry Data Center
InChIKey: NKGGFTFDYGTUSL-UHFFFAOYSA-N Non-stereo
10 largest peaks:

| | | | | | | | | | |
|---|---|---|---|---|---|---|---|---|---|
| 271 999 | 189 364 | 175 337 | 201 280 | 272 207 | 69 181 | 302 128 | 229 111 | 187 107 | 215 88 |

108 m/z Values and Intensities:

| | | | | | | | | | |
|---|---|---|---|---|---|---|---|---|---|
| 31 7 | 39 3 | 41 33 | 42 2 | 43 31 | 53 5 | 55 27 | 56 5 | 57 5 | 65 5 |
| 67 10 | 69 181 | 70 9 | 77 13 | 78 4 | 79 9 | 81 6 | 83 20 | 85 9 | 86 2 |
| 91 25 | 92 5 | 93 5 | 97 8 | 103 6 | 105 16 | 106 3 | 107 21 | 109 8 | 115 51 |
| 116 12 | 117 16 | 118 3 | 119 5 | 121 9 | 127 25 | 128 50 | 129 37 | 130 7 | 131 30 |
| 132 11 | 133 64 | 141 43 | 142 14 | 143 15 | 144 27 | 145 72 | 146 11 | 147 48 | 148 6 |
| 149 43 | 150 4 | 152 13 | 153 17 | 154 10 | 155 17 | 156 4 | 157 50 | 158 22 | 159 65 |
| 160 9 | 161 28 | 162 6 | 163 15 | 164 17 | 165 11 | 169 12 | 170 11 | 171 41 | 172 16 |

| | | | | | | | | | |
|---|---|---|---|---|---|---|---|---|---|
| 173 71 | 174 11 | 175 337 | 176 43 | 177 9 | 183 10 | 184 9 | 185 40 | 186 16 | 187 107 |
| 188 17 | 189 364 | 190 49 | 199 41 | 200 12 | 201 280 | 202 62 | 203 54 | 204 6 | 211 14 |
| 213 21 | 214 6 | 215 88 | 216 15 | 229 111 | 230 20 | 255 9 | 256 7 | 257 3 | 259 2 |
| 271 999 | 272 207 | 273 23 | 274 2 | 301 2 | 302 128 | 303 27 | 304 4 | | |

Synonyms:
1.4a(2H)-Phenanthrenemethanol, 1,3,4,9,10,10a-hexahydro-6-hydroxy-1,1-dimethyl-7-(1-methylethyl)-, (4aR,10aS)-
2.4a(2H)-Phenanthrenemethanol, 1,3,4,9,10,10a-hexahydro-6-hydroxy-1,1-dimethyl-7-(1-methylethyl)-, (4aR-trans)-
3.Podocarpa-8,11,13-triene-12,17-diol, 13-isopropyl-
4.(+)-Pisiferol

Experimental RI median±deviation (#data)
Semi-standard non-polar:2615±N/A (1)

Hit 6 : (4Br,8As)-(+)-4B,5,6,7,8,8A,9,10-octahydro-2-isopropyl-4B-((methoxymethoxy)methyl)-8,8-dimethylphenanthre
C22H34O3; MF: 680; RMF: 769; Prob 4.61%; Lib: mainlib; ID: 238799.

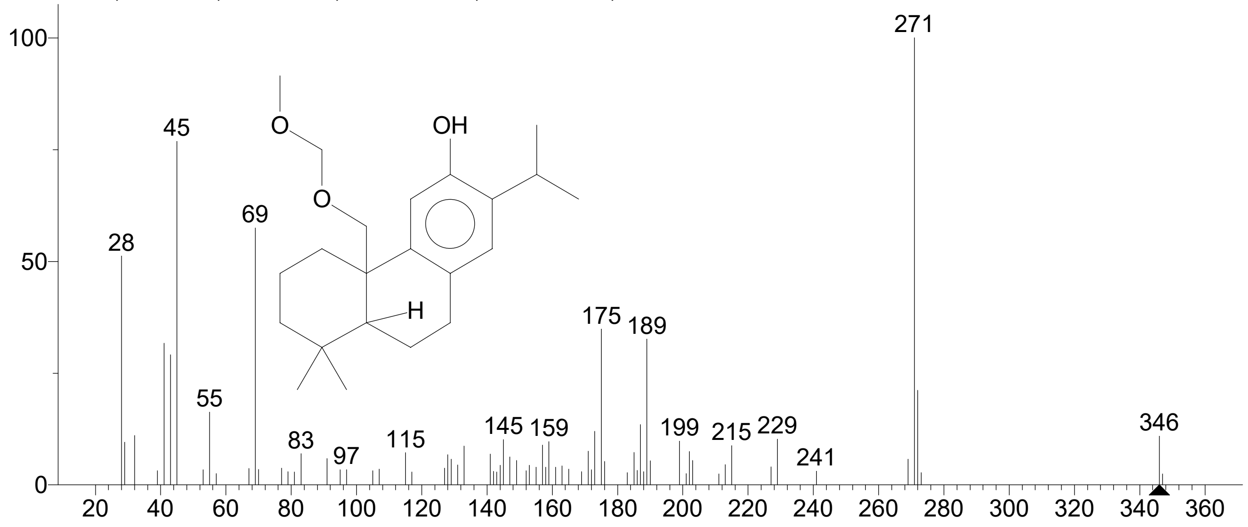


(mainlib) (4Br,8As)-(+)-4B,5,6,7,8,8A,9,10-octahydro-2-isopropyl-4B-((methoxymethoxy)methyl)-8,8-dimethylphenanthren-3-ol

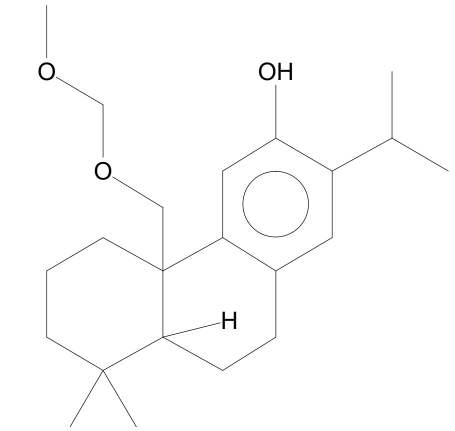


Name: (4Br,8As)-(+)-4B,5,6,7,8,8A,9,10-octahydro-2-isopropyl-4B-((methoxymethoxy)methyl)-8,8
-dimethylphenanthren-3-ol
Formula: $C_{22}H_{34}O_3$
MW: 346 Exact Mass: 346.250795 NIST#: 426722 ID#: 238799 DB: mainlib
Contributor: HAGIWARA H, CHEM. RES. INST. OF NON-AQUEOUS SOLUTIONS, TOHOKU UNIV.; Used with permission, courtesy of Funatsu Laboratory, University of Tokyo.The copyright reserved by Funatsu Laboratory.
InChIKey: MODQAVGXDPNSOD-UHFFFAOYSA-N Non-stereo
10 largest peaks:

| | | | | | | | | | |
|---|---|---|---|---|---|---|---|---|---|
| 271 999 | 45 768 | 69 575 | 28 511 | 175 348 | 189 326 | 41 316 | 43 290 | 272 211 | 55 163 |

74 m/z Values and Intensities:

| | | | | | | | | | |
|---|---|---|---|---|---|---|---|---|---|
| 28 511 | 29 95 | 32 110 | 39 31 | 41 316 | 43 290 | 45 768 | 53 33 | 55 163 | 57 25 |
| 67 36 | 69 575 | 70 34 | 77 37 | 79 29 | 81 28 | 83 70 | 91 58 | 95 33 | 97 35 |
| 105 31 | 107 35 | 115 72 | 117 28 | 127 37 | 128 67 | 129 57 | 131 44 | 133 86 | 141 68 |
| 142 30 | 143 28 | 144 43 | 145 102 | 147 62 | 149 54 | 152 31 | 153 43 | 155 39 | 157 88 |
| 158 39 | 159 97 | 161 39 | 163 42 | 165 35 | 169 29 | 171 75 | 172 33 | 173 119 | 175 348 |
| 176 52 | 183 27 | 185 72 | 186 32 | 187 134 | 188 29 | 189 326 | 190 53 | 199 98 | 201 25 |

202 74 | 203 54 | 211 24 | 213 45 | 215 88 | 227 40 | 229 103 | 241 31 | 269 57 | 271 999 |
272 211 | 273 27 | 346 110 | 347 24 |

Synonyms:

1.Pisiferol (methoxymethoxy)methyl ethre

Hit 7 : 2-Phenanthrenol, 4b,5,6,7,8,8a,9,10-octahydro-4b,8,8-trimethyl-1-(1-methylethyl)-, acetate, (4bS-trans)-
C22H32O2; MF: 657; RMF: 684; Prob 1.68%; CAS: 15340-82-6; Lib: mainlib; ID: 239104.

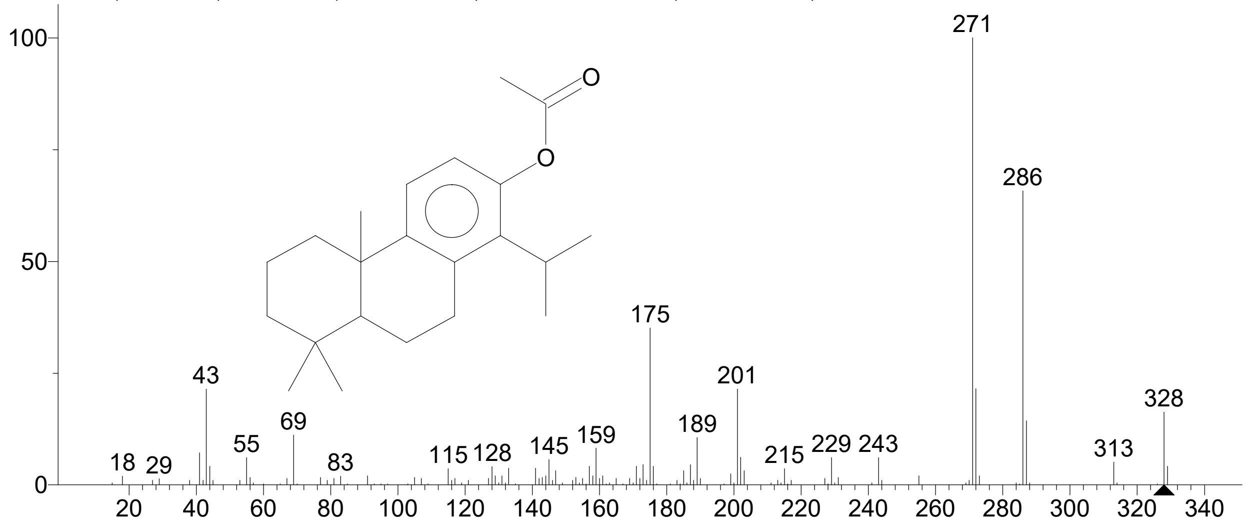


(mainlib) 2-Phenanthrenol, 4b,5,6,7,8,8a,9,10-octahydro-4b,8,8-trimethyl-1-(1-methylethyl)-, acetate, (4bS-trans)-

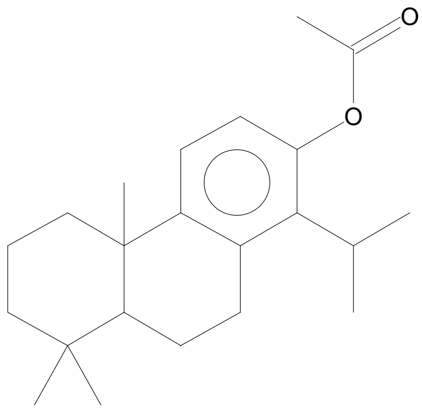


Name: 2-Phenanthrenol, 4b,5,6,7,8,8a,9,10-octahydro-4b,8,8-trimethyl-1-(1-methylethyl)-, acetate, (4bS-trans)-
Formula: $C_{22}H_{32}O_2$
MW: 328 Exact Mass: 328.24023 CAS#: 15340-82-6 NIST#: 15316 ID#: 239104 DB: mainlib
Other DBs: None
InChIKey: ORVBSFQTFRBNRP-UHFFFAOYSA-N   Non-stereo
10 largest peaks:

| | | | | | | | | | |
|---|---|---|---|---|---|---|---|---|---|
| 271 999 | 286 657 | 175 351 | 43 214 | 201 214 | 272 214 | 328 163 | 287 143 | 69 112 | 189 106 |

121 m/z Values and Intensities:

| | | | | | | | | | |
|---|---|---|---|---|---|---|---|---|---|
| 15 4 | 18 20 | 27 10 | 29 14 | 38 10 | 41 71 | 42 10 | 43 214 | 44 41 | 45 10 |
| 53 10 | 55 61 | 56 16 | 57 4 | 60 2 | 65 2 | 67 14 | 69 112 | 70 2 | 77 16 |
| 79 10 | 81 14 | 83 20 | 85 2 | 91 20 | 93 2 | 95 2 | 97 2 | 103 2 | 105 16 |
| 107 14 | 109 2 | 115 37 | 116 10 | 117 14 | 119 4 | 121 10 | 127 14 | 128 41 | 129 20 |
| 130 4 | 131 20 | 132 4 | 133 37 | 135 2 | 141 37 | 142 14 | 143 16 | 144 20 | 145 57 |
| 146 10 | 147 31 | 149 4 | 152 10 | 153 16 | 154 4 | 155 14 | 156 2 | 157 41 | 158 20 |
| 159 82 | 160 14 | 161 20 | 162 2 | 163 4 | 165 14 | 167 2 | 169 14 | 170 4 | 171 41 |
| 172 14 | 173 45 | 174 10 | 175 351 | 176 41 | 177 2 | 181 2 | 183 10 | 184 2 | 185 31 |

186 4 | 187 45 | 188 10 | 189 106 | 190 14 | 197 2 | 199 24 | 200 4 | 201 214 | 202 61 |
203 31 | 211 4 | 213 10 | 214 4 | 215 37 | 216 2 | 217 10 | 227 14 | 228 2 | 229 61 |
230 4 | 231 16 | 241 4 | 243 61 | 244 10 | 255 20 | 269 4 | 270 10 | 271 999 | 272 214 |
273 20 | 284 4 | 285 2 | 286 657 | 287 143 | 288 4 | 313 51 | 314 4 | 326 2 | 328 163 |
329 41 |

Synonyms:

1.Podocarpa-8,11,13-trien-13-ol, 14-isopropyl-, acetate
2.Totaryl acetate
3.14-Isopropylpodocarpa-8,11,13-trien-13-yl acetate #
4.Totarol acetate
5.2-Phenanthrenol, 4b,5,6,7,8,8a,9,10-octahydro-4b,8,8-trimethyl-1-(1-methylethyl)-, 2-acetate, (4bS,8aS)-
6.2-Phenanthrenol, 4b,5,6,7,8,8a,9,10-octahydro-4b,8,8-trimethyl-1-(1-methylethyl)-, acetate, (4bS,8aS)-

Experimental RI median±deviation (#data)
Semi-standard non-polar:2417±0 (2)
Standard non-polar: 2437±0 (2)

Hit 8 : 1-Phenanthrenemethanol, 1,2,3,4,4a,9,10,10a-octahydro-1,4a-dimethyl-7-(1-methylethyl)-, [1S-(1α,4aα,10aβ)]-
$C_{20}H_{30}O$; MF: 639; RMF: 711; Prob 0.86%; CAS: 24035-43-6; Lib: mainlib; ID: 238904.

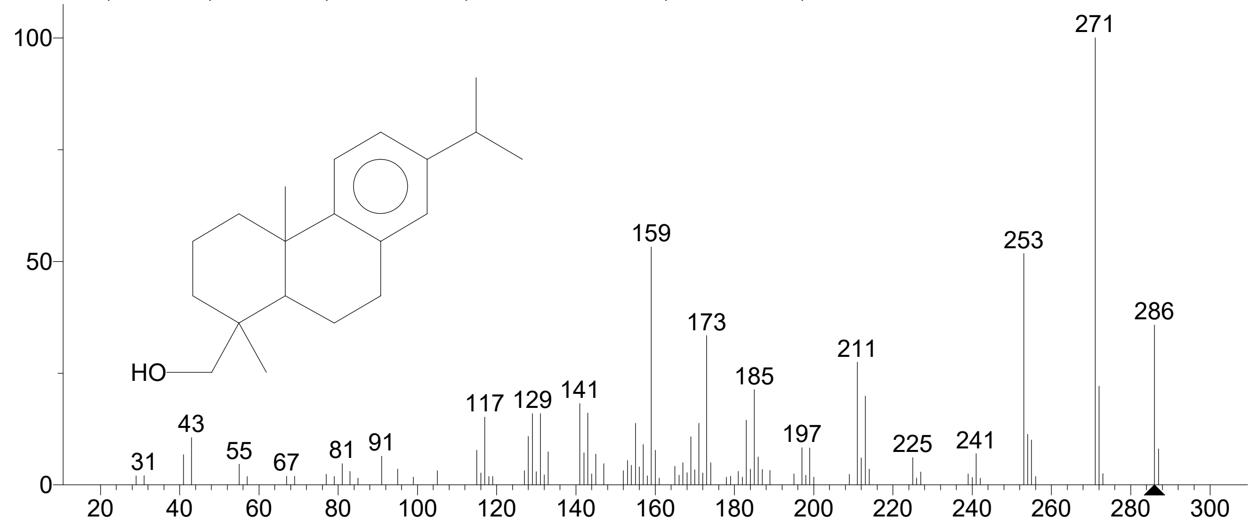

(mainlib) 1-Phenanthrenemethanol, 1,2,3,4,4a,9,10,10a-octahydro-1,4a-dimethyl-7-(1-methylethyl)-, [1S-(1α,4aα,10aβ)]-

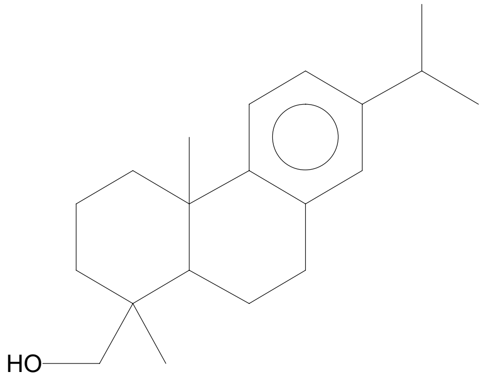


Name: 1-Phenanthrenemethanol, 1,2,3,4,4a,9,10,10a-octahydro-1,4a-dimethyl-7-(1-methylethyl)-, [1S-(1α,4aα,10aβ)
]-
Formula: $C_{20}H_{30}O$
MW: 286 Exact Mass: 286.229666 CAS#: 24035-43-6 NIST#: 42590 ID#: 238904 DB: mainlib
Other DBs: None
Contributor: R RYHAGE MS-LAB KAROLINSKA INSTITUTET STOCKHOLM SWEDEN
InChIKey: WSKGRAGZAQRSED-UHFFFAOYSA-N Non-stereo
10 largest peaks:

| | | | | | | | | | |
|---|---|---|---|---|---|---|---|---|---|
| 271 999 | 159 532 | 253 517 | 286 357 | 173 334 | 211 274 | 272 220 | 185 213 | 213 198 | 141 182 |

91 m/z Values and Intensities:

| | | | | | | | | | |
|---|---|---|---|---|---|---|---|---|---|
| 29 20 | 31 21 | 41 67 | 43 106 | 55 46 | 57 18 | 67 20 | 69 20 | 77 23 | 79 18 |
| 81 48 | 83 30 | 85 15 | 91 65 | 95 35 | 99 17 | 105 31 | 115 77 | 116 26 | 117 152 |
| 118 18 | 119 18 | 127 31 | 128 108 | 129 160 | 130 29 | 131 160 | 132 22 | 133 73 | 141 182 |
| 142 71 | 143 160 | 144 24 | 145 68 | 147 47 | 152 31 | 153 54 | 154 43 | 155 137 | 156 40 |
| 157 90 | 158 20 | 159 532 | 160 77 | 161 15 | 165 41 | 166 21 | 167 49 | 168 27 | 169 107 |
| 170 33 | 171 137 | 172 26 | 173 334 | 174 49 | 178 17 | 179 19 | 181 30 | 182 17 | 183 144 |

| | | | | | | | | | |
|---|---|---|---|---|---|---|---|---|---|
| 184 35 | 185 213 | 186 62 | 187 34 | 189 32 | 195 24 | 197 84 | 198 21 | 199 82 | 200 17 |
| 209 23 | 211 274 | 212 60 | 213 198 | 214 35 | 225 61 | 226 15 | 227 28 | 239 24 | 240 16 |
| 241 70 | 242 15 | 253 517 | 254 113 | 255 100 | 256 18 | 271 999 | 272 220 | 273 25 | 286 357 |
| 287 80 | | | | | | | | | |

Synonyms:
1.4-Epiabietol, dehydro-
2.Podocarpa-8,11,13-trien-16-ol, 13-isopropyl-
3.1-Phenanthrenemethanol, 1,2,3,4,4a,9,10,10a-octahydro-1,4a-dimethyl-7-(1-methylethyl)-, (1S,4aS,10aR)-
4.4-Epidehydroabietol
5.4-epi-Dehydroabietol

Hit 9 : 1-Phenanthrenemethanol, 1,2,3,4,4a,9,10,10a-octahydro-1,4a-dimethyl-7-(1-methylethyl)-, [1R-(1α,4aβ,10aα)]-
$C_{20}H_{30}O$; MF: 628; RMF: 630; Prob 0.59%; CAS: 3772-55-2; Lib: replib; ID: 36422.

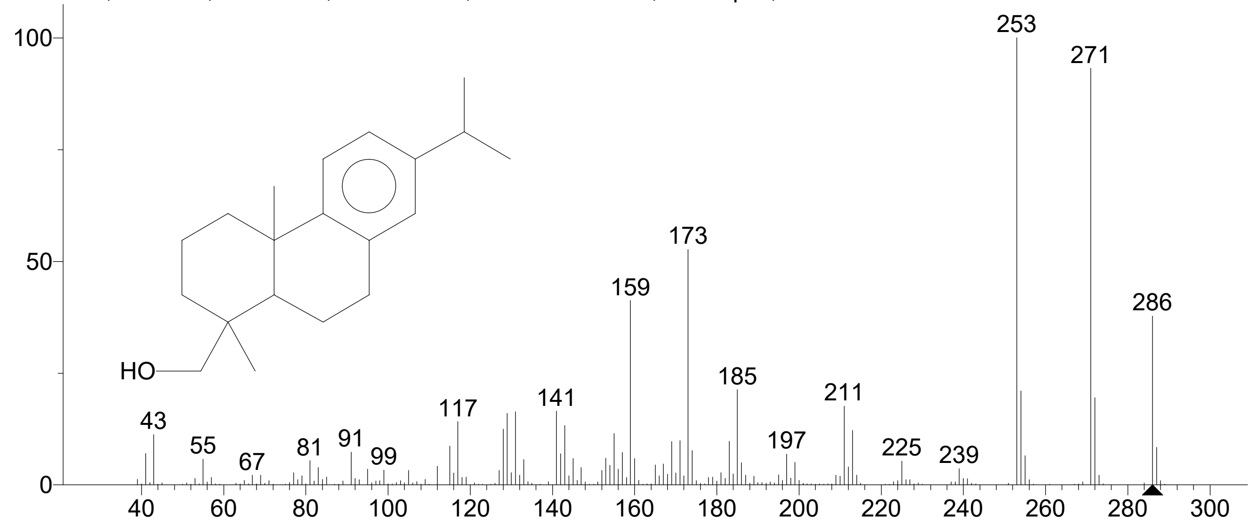

(replib) 1-Phenanthrenemethanol, 1,2,3,4,4a,9,10,10a-octahydro-1,4a-dimethyl-7-(1-methylethyl)-, [1R-(1α,4aβ,10aα)]-

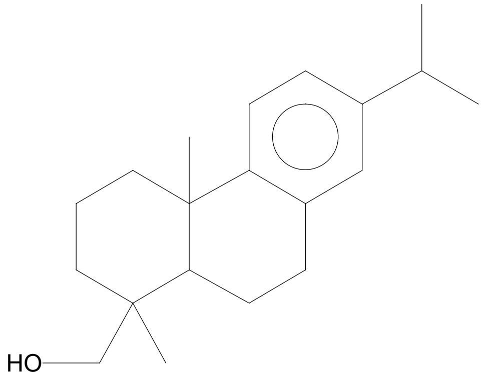


Name: 1-Phenanthrenemethanol, 1,2,3,4,4a,9,10,10a-octahydro-1,4a-dimethyl-7-(1-methylethyl)-, [1R-(1α,4aβ,10aα)
]-
Formula: $C_{20}H_{30}O$
MW: 286 Exact Mass: 286.229666 CAS#: 3772-55-2 NIST#: 385640 ID#: 36422 DB: replib
Other DBs: TSCA, EINECS
Contributor: T. Huret, J. Little, Eastman Chemical Co.
InChIKey: WSKGRAGZAQRSED-UHFFFAOYSA-N Non-stereo
10 largest peaks:
253 999 | 271 930 | 173 526 | 159 412 | 286 378 | 185 213 | 254 209 | 272 194 | 211 176 | 141 165 |
193 m/z Values and Intensities:

| | | | | | | | | | |
|---|---|---|---|---|---|---|---|---|---|
| 39 12 | 40 2 | 41 70 | 42 5 | 43 113 | 44 1 | 45 4 | 50 1 | 51 4 | 52 1 |
| 53 14 | 54 2 | 55 58 | 56 6 | 57 16 | 58 2 | 59 1 | 63 3 | 64 2 | 65 10 |
| 66 2 | 67 22 | 68 2 | 69 22 | 70 3 | 71 9 | 72 1 | 75 1 | 76 5 | 77 27 |
| 78 11 | 79 20 | 80 2 | 81 54 | 82 8 | 83 38 | 84 12 | 85 17 | 87 1 | 88 1 |
| 89 8 | 91 74 | 92 14 | 93 11 | 95 35 | 96 4 | 97 8 | 98 9 | 99 33 | 100 2 |
| 101 2 | 102 5 | 103 9 | 104 4 | 105 32 | 106 4 | 107 7 | 108 1 | 109 12 | 110 1 |

| | | | | | | | | | |
|---|---|---|---|---|---|---|---|---|---|
| 112 41 | 115 86 | 116 26 | 117 141 | 118 16 | 119 17 | 121 4 | 122 1 | 123 1 | 125 1 |
| 126 3 | 127 32 | 128 124 | 129 159 | 130 27 | 131 163 | 132 21 | 133 56 | 134 7 | 135 3 |
| 137 1 | 139 7 | 141 165 | 142 69 | 143 132 | 144 20 | 145 58 | 146 10 | 147 38 | 148 6 |
| 149 1 | 150 1 | 151 6 | 152 32 | 153 59 | 154 43 | 155 114 | 156 35 | 157 72 | 158 16 |
| 159 412 | 160 58 | 161 10 | 162 1 | 163 2 | 164 2 | 165 44 | 166 20 | 167 46 | 168 23 |
| 169 96 | 170 26 | 171 98 | 172 20 | 173 526 | 174 76 | 175 9 | 176 3 | 177 2 | 178 16 |
| 179 17 | 180 8 | 181 27 | 182 14 | 183 97 | 184 24 | 185 213 | 186 49 | 187 21 | 188 3 |
| 189 19 | 190 5 | 191 5 | 192 3 | 193 6 | 194 4 | 195 22 | 196 10 | 197 69 | 198 15 |
| 199 50 | 200 10 | 201 3 | 202 2 | 203 2 | 204 1 | 205 2 | 206 1 | 207 2 | 208 2 |
| 209 21 | 210 19 | 211 176 | 212 40 | 213 121 | 214 21 | 215 3 | 221 1 | 222 1 | 223 7 |
| 224 9 | 225 53 | 226 11 | 227 11 | 228 2 | 229 4 | 230 1 | 237 6 | 238 6 | 239 37 |
| 240 14 | 241 13 | 242 3 | 243 2 | 251 3 | 253 999 | 254 209 | 255 65 | 256 11 | 257 1 |
| 261 1 | 267 1 | 268 2 | 269 6 | 271 930 | 272 194 | 273 21 | 274 2 | 284 4 | 286 378 |
| 287 83 | 288 9 | 289 1 | | | | | | | |

Synonyms:
1.Podocarpa-8,11,13-trien-15-ol, 13-isopropyl-
2.Abietyl alcohol, dehydro-
3.Dehydroabeityl alcohol
4.Dehydroabietinol
5.Dehydroabietol
6.Abieta-8,11,13-trien-18-ol #

Experimental RI median±deviation (#data)
Semi-standard non-polar:2360±3 (6)
Standard non-polar: 2318±N/A (1)
Polar: 3100±N/A (1)

Hit 10 : 1-Phenanthrenemethanol, 1,2,3,4,4a,9,10,10a-octahydro-1,4a-dimethyl-7-(1-methylethyl)-, [1R-(1α,4aβ,10aα)
$C_{20}H_{30}O$; MF: 627; RMF: 684; Prob 0.59%; CAS: 3772-55-2; Lib: mainlib; ID: 231887.

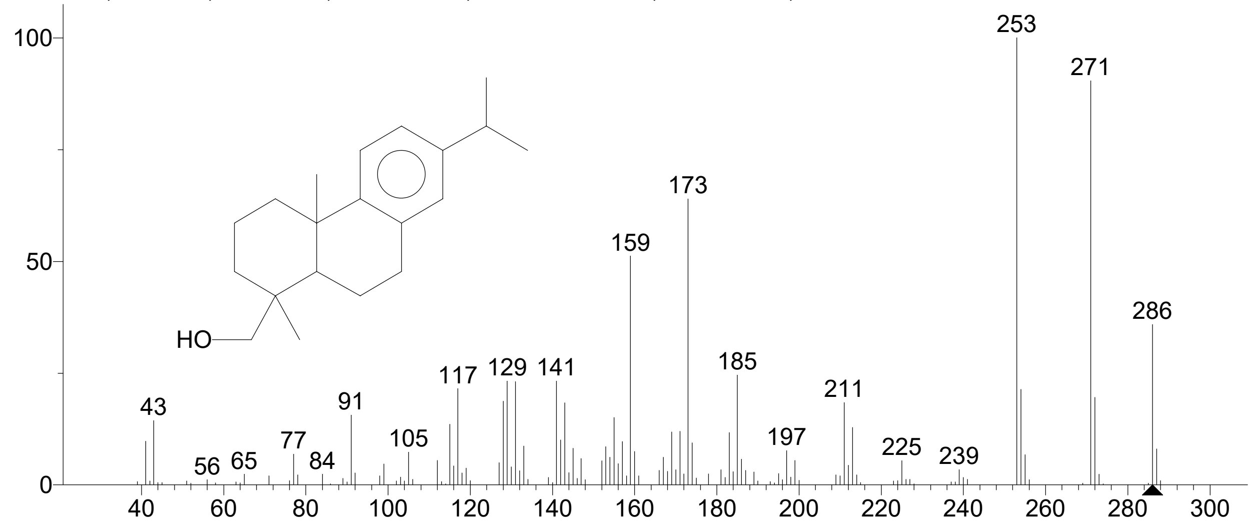


(mainlib) 1-Phenanthrenemethanol, 1,2,3,4,4a,9,10,10a-octahydro-1,4a-dimethyl-7-(1-methylethyl)-, [1R-(1α,4aβ,10aα)]-

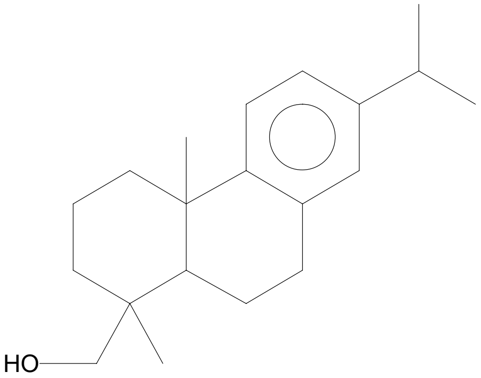


Name: 1-Phenanthrenemethanol, 1,2,3,4,4a,9,10,10a-octahydro-1,4a-dimethyl-7-(1-methylethyl)-, [1R-(1α,4aβ,10aα)
]-
Formula: $C_{20}H_{30}O$
MW: 286 Exact Mass: 286.229666 CAS#: 3772-55-2 NIST#: 412371 ID#: 231887 DB: mainlib
Other DBs: TSCA, EINECS
Contributor: NIST Mass Spectrometry Data Center
InChIKey: WSKGRAGZAQRSED-UHFFFAOYSA-N Non-stereo
10 largest peaks:

| | | | | | | | | | |
|---|---|---|---|---|---|---|---|---|---|
| 253 999 | 271 903 | 173 639 | 159 511 | 286 359 | 185 246 | 129 232 | 141 232 | 131 230 | 117 215 |

126 m/z Values and Intensities:

| | | | | | | | | | |
|---|---|---|---|---|---|---|---|---|---|
| 39 7 | 41 97 | 42 8 | 43 144 | 44 5 | 45 5 | 51 8 | 52 3 | 56 12 | 58 4 |
| 63 6 | 64 4 | 65 24 | 71 20 | 76 9 | 77 69 | 78 22 | 84 24 | 86 2 | 89 14 |
| 90 6 | 91 156 | 92 26 | 98 20 | 99 46 | 102 8 | 103 17 | 104 8 | 105 73 | 106 12 |
| 112 54 | 113 7 | 114 2 | 115 135 | 116 42 | 117 215 | 118 26 | 119 37 | 120 9 | 127 49 |
| 128 186 | 129 232 | 130 40 | 131 230 | 132 31 | 133 86 | 134 12 | 139 16 | 140 5 | 141 232 |
| 142 100 | 143 183 | 144 27 | 145 81 | 146 14 | 147 58 | 148 11 | 152 53 | 153 85 | 154 61 |

155 150 | 156 47 | 157 96 | 158 20 | 159 511 | 160 74 | 161 20 | 166 32 | 167 61 | 168 30 |
169 118 | 170 33 | 171 119 | 172 24 | 173 639 | 174 93 | 175 15 | 178 24 | 181 33 | 182 16 |
183 116 | 184 29 | 185 246 | 186 57 | 187 32 | 189 28 | 190 8 | 193 7 | 194 4 | 195 25 |
196 11 | 197 77 | 198 17 | 199 54 | 200 10 | 209 22 | 210 20 | 211 184 | 212 43 | 213 128 |
214 22 | 215 5 | 223 8 | 224 9 | 225 55 | 226 12 | 227 12 | 228 2 | 237 6 | 238 6 |
239 35 | 240 16 | 241 12 | 253 999 | 254 213 | 255 67 | 256 11 | 269 3 | 271 903 | 272 195 |
273 23 | 274 2 | 285 3 | 286 359 | 287 80 | 288 9 |

Synonyms:
1.Podocarpa-8,11,13-trien-15-ol, 13-isopropyl-
2.Abietyl alcohol, dehydro-
3.Dehydroabeityl alcohol
4.Dehydroabietinol
5.Dehydroabietol
6.Abieta-8,11,13-trien-18-ol #

Experimental RI median±deviation (#data)
Semi-standard non-polar:2360±3 (6)
Standard non-polar: 2318±N/A (1)
Polar: 3100±N/A (1)
