# Supplementary material for: Uncovering a miltiradiene biosynthetic gene cluster in the Lamiaceae reveals a dynamic evolutionary trajectory
Source: Nat Commun. 2023 Jan 20;14:343. doi: 10.1038/s41467-023-35845-1 (PMC9860074; doi:10.1038/s41467-023-35845-1)
Supplement: Supplementary file 4 — Source Data [file 41467_2023_35845_MOESM4_ESM.zip › Supplementary Fig. 12 Source Data.pdf]

Unknown; InLib=-1302

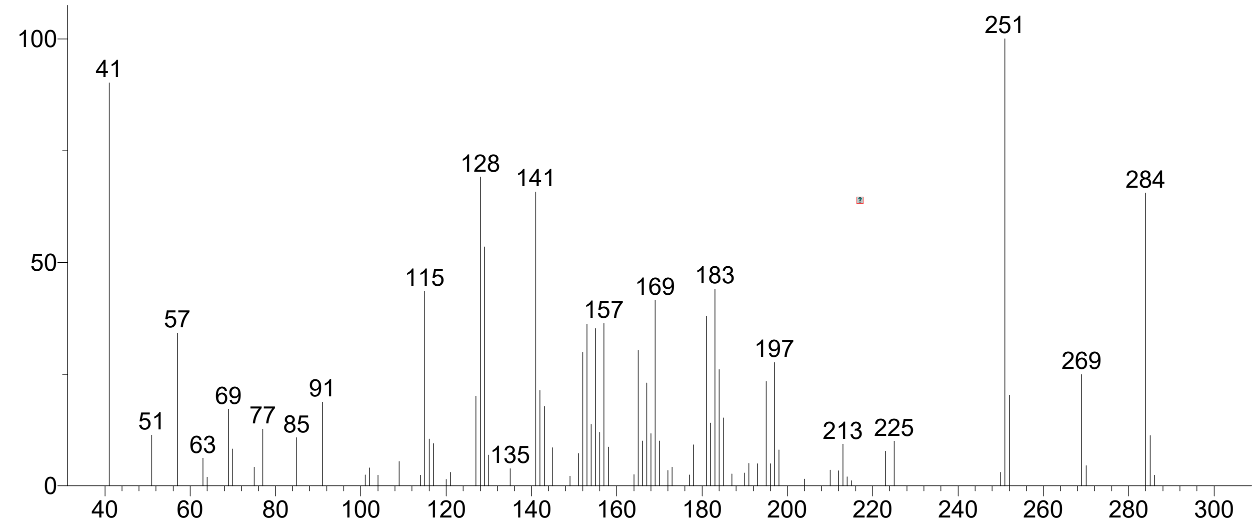

(Text File) Component at scan 2581 (15.002 min) [Model = +284u] in X:\ABBY GCMS\20211015\A_211015_020.D\DATA.MS

Name: Component at scan 2581 (15.002 min) [Model = +284u] in X:\ABBY GCMS\20211015\A_211015_020.
D\DATA.MS
MW: N/A ID#: 1016 DB: Text File
10 largest peaks:

251 999	41 901	128 690	141 657	284 654	129 533	183 440	115 435	169 415	181 379

79 m/z Values and Intensities:

41 901	51 114	57 342	63 62	64 19	69 171	70 82	75 41	77 128	85 108
91 188	101 24	102 40	104 23	109 54	114 23	115 435	116 104	117 94	120 14
121 30	127 200	128 690	129 533	130 68	135 39	141 657	142 213	143 177	145 85
149 21	151 72	152 298	153 361	154 137	155 351	156 119	157 363	158 86	164 25
165 302	166 100	167 229	168 116	169 415	170 100	172 34	173 41	177 24	178 91
181 379	182 140	183 440	184 259	185 151	187 26	190 28	191 50	193 49	195 233
196 49	197 276	198 80	204 15	210 35	212 33	213 93	214 20	215 11	223 77
225 100	250 30	251 999	252 202	269 249	270 45	284 654	285 112	286 23	

Synonyms:
no synonyms.

Hit 1 : Abieta-8,11,13-trien-3-one
C20H28O; MF: 608; RMF: 630; Prob 13.4%; Lib: mainlib; ID: 238140.

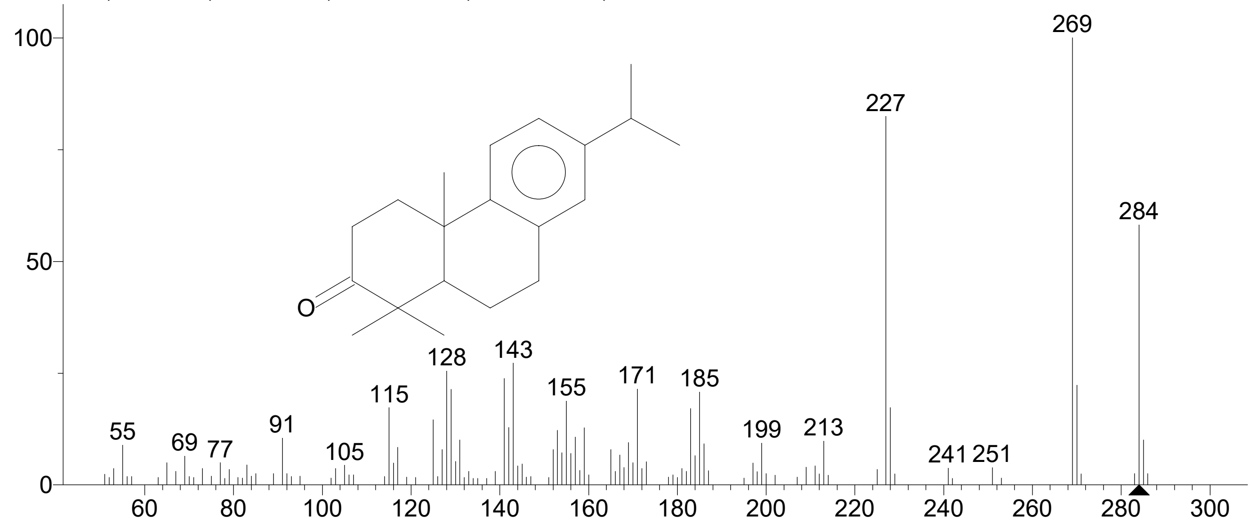

(mainlib) Abieta-8,11,13-trien-3-one

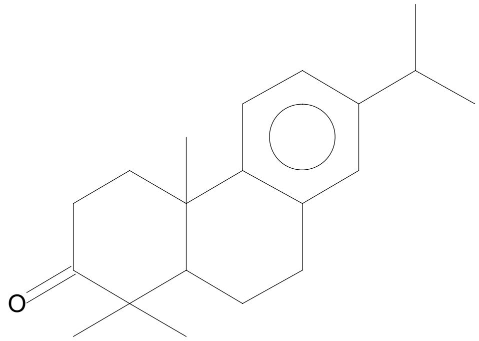

Name: Abieta-8,11,13-trien-3-one
Formula: $C_{20}H_{28}O$
MW: 284 Exact Mass: 284.214016 NIST#: 386463 ID#: 238140 DB: mainlib
Contributor: Robert E. Cox et al. Biochemical Systematics and Ecology 35 (2007) 342-362
InChIKey: ANVLVIISBTWDRN-UHFFFAOYSA-N   Non-stereo
10 largest peaks:

269 999	227 823	284 581	143 272	128 255	141 237	270 222	171 214	129 213	185 208

114 m/z Values and Intensities:

51 23	52 16	53 36	55 89	56 18	57 18	63 16	65 49	67 30	69 65
70 18	71 16	73 36	75 19	77 50	78 14	79 34	81 16	82 15	83 44
84 19	85 25	89 25	91 105	92 25	93 18	95 19	102 15	103 36	105 44
106 22	107 22	114 18	115 173	116 48	117 83	119 17	121 16	125 145	126 18
127 78	128 255	129 213	130 52	131 100	132 16	133 30	134 14	135 14	137 14
139 30	141 237	142 128	143 272	144 42	145 46	146 17	147 18	151 16	152 78
153 121	154 71	155 188	156 70	157 106	158 32	159 127	160 22	165 78	166 30
167 66	168 38	169 94	170 49	171 214	172 36	173 51	178 17	179 22	180 16

181 36	182 30	183 170	184 65	185 208	186 91	187 31	195 15	197 48	198 29
199 94	200 25	202 21	207 17	209 39	211 42	212 24	213 98	214 21	225 34
227 823	228 172	229 24	241 38	242 14	251 39	253 15	269 999	270 222	271 24
283 25	284 581	285 100	286 25						

Synonyms:
no synonyms.

Hit 2 : 1-Phenanthrenecarboxaldehyde, 1,2,3,4,4a,9,10,10a-octahydro-1,4a-dimethyl-7-(1-methylethyl)-, [1R-(1α,4aβ,
C20H28O; MF: 605; RMF: 613; Prob 11.9%; CAS: 13601-88-2; Lib: replib; ID: 3937.

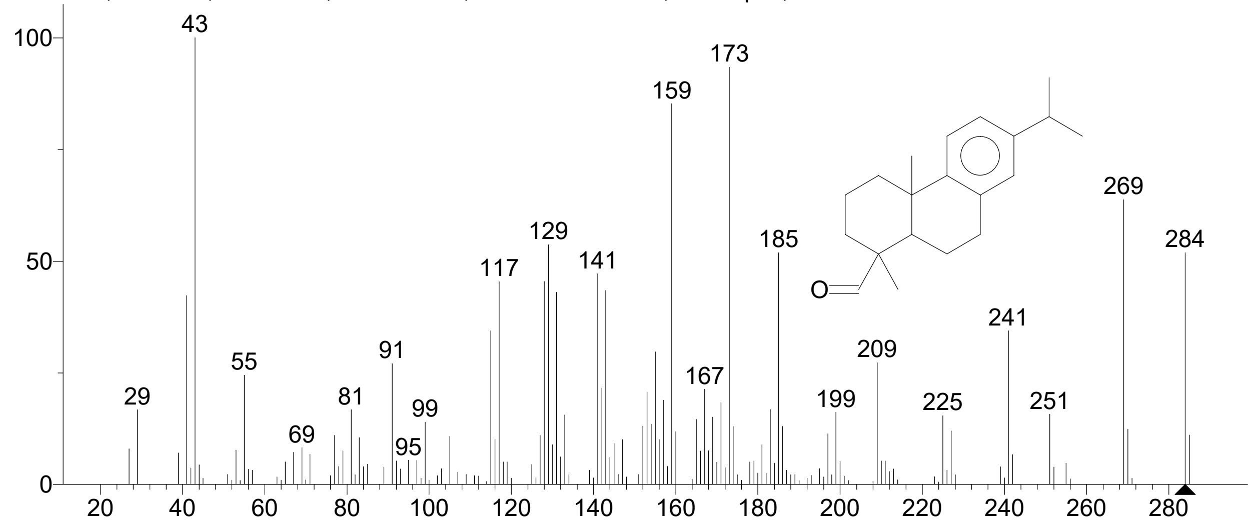

(replib) 1-Phenanthrenecarboxaldehyde, 1,2,3,4,4a,9,10,10a-octahydro-1,4a-dimethyl-7-(1-methylethyl)-, [1R-(1α,4aβ,10aα)]-

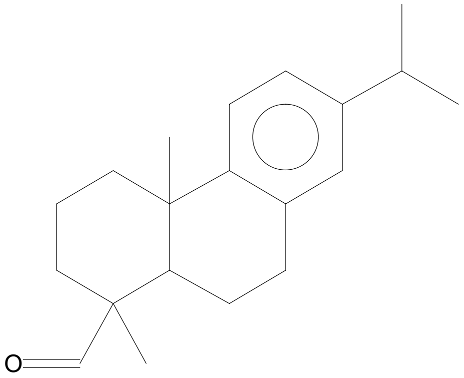

Name: 1-Phenanthrenecarboxaldehyde, 1,2,3,4,4a,9,10,10a-octahydro-1,4a-dimethyl-7-(1-methylethyl)-, [1R-(1α,
4aβ,10aα)]-
Formula: $C_{20}H_{28}O$
MW: 284 Exact Mass: 284.214016 CAS#: 13601-88-2 NIST#: 384007 ID#: 3937 DB: replib
Other DBs: None
Contributor: NIST Mass Spectrometry Data Center, 2010
InChIKey: YCLCHPWRGSDZKL-UHFFFAOYSA-N Non-stereo
10 largest peaks:

43 999	173 933	159 851	269 637	129 536	185 518	284 518	141 471	117 454	128 453

146 m/z Values and Intensities:

27 79	29 167	39 70	41 422	42 36	43 999	44 43	45 13	51 22	52 9
53 76	54 8	55 245	56 33	57 31	63 16	64 9	65 50	67 71	69 82
70 10	71 67	76 19	77 109	78 40	79 75	81 167	82 21	83 104	84 39
85 45	89 38	91 270	92 52	93 34	95 54	97 53	98 13	99 140	100 9
102 19	103 35	105 107	107 27	109 22	111 19	112 18	114 6	115 343	116 100
117 454	118 50	119 50	120 13	125 44	126 15	127 109	128 453	129 536	130 88

131 429 | 132 61 | 133 155 | 134 21 | 139 31 | 140 14 | 141 471 | 142 215 | 143 433 | 144 60 |
145 91 | 146 22 | 147 100 | 148 16 | 151 22 | 152 130 | 153 206 | 154 134 | 155 296 | 156 100 |
157 188 | 158 40 | 159 851 | 160 118 | 164 11 | 165 145 | 166 74 | 167 213 | 168 75 | 169 150 |
170 49 | 171 183 | 172 37 | 173 933 | 174 129 | 175 21 | 176 9 | 178 50 | 179 52 | 180 25 |
181 88 | 182 25 | 183 167 | 184 47 | 185 518 | 186 129 | 187 31 | 188 21 | 189 22 | 190 8 |
192 13 | 193 20 | 195 35 | 196 16 | 197 113 | 198 21 | 199 161 | 200 51 | 201 18 | 202 8 |
208 7 | 209 273 | 210 52 | 211 52 | 212 28 | 213 34 | 214 10 | 223 17 | 224 5 | 225 154 |
226 31 | 227 119 | 228 21 | 239 39 | 240 14 | 241 344 | 242 66 | 251 157 | 252 38 | 255 47 |
256 12 | 269 637 | 270 123 | 271 13 | 284 518 | 285 110 |

Synonyms:
1.(1R,4aS,10aR)-7-Isopropyl-1,4a-dimethyl-1,2,3,4,4a,9,10,10a-octahydrophenanthrene-1-carbaldehyde
2.Podocarpa-8,11,13-trien-15-al, 13-isopropyl-
3.Dehydroabietal
4.Dehydroabietic aldehyde
5.Dehydroabietinal
6.Abietaldehyde, dehydro-
7.Abieta-8,11,13-trien-18-al #

Experimental RI median±deviation (#data)
Semi-standard non-polar:2263±2 (8)
Standard non-polar: 2255±13 (7)
Polar: 2979±3 (2)

Hit 3 : Methyl 2-(4-methoxyphenyl)-6,7-dihydrobenzofuran-3-carboxylate
C17H16O4; MF: 605; RMF: 611; Prob 11.9%; Lib: mainlib; ID: 243098.

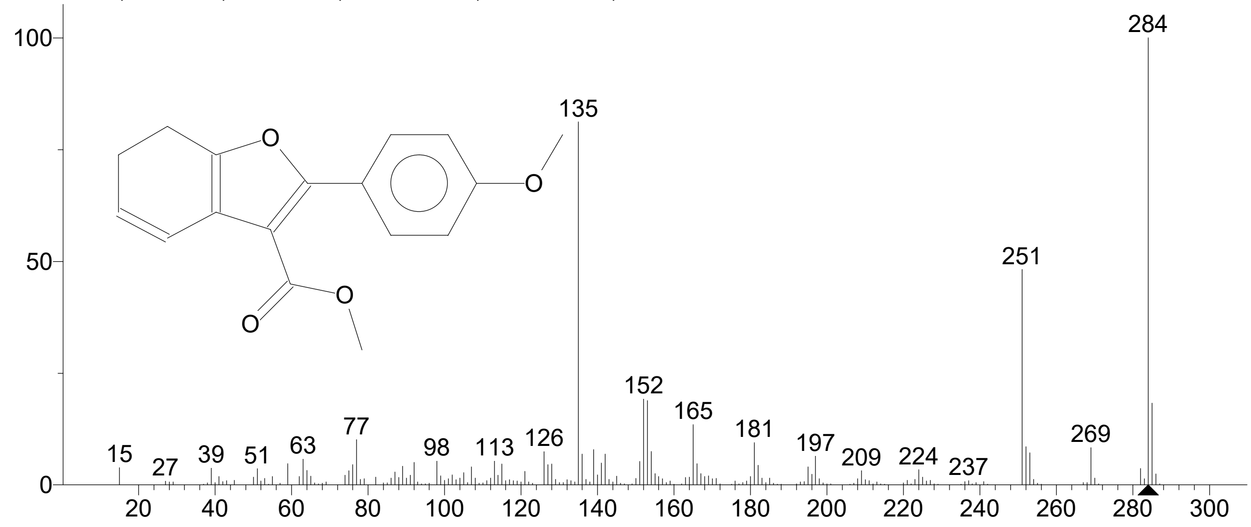

(mainlib) Methyl 2-(4-methoxyphenyl)-6,7-dihydrobenzofuran-3-carboxylate

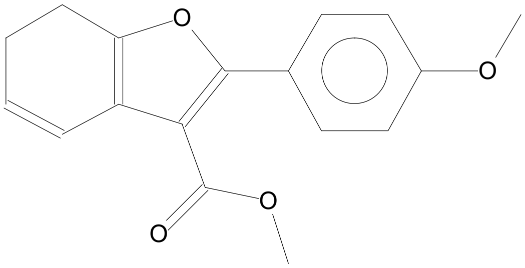

Name: Methyl 2-(4-methoxyphenyl)-6,7-dihydrobenzofuran-3-carboxylate
Formula: $C_{17}H_{16}O_4$
MW: 284 Exact Mass: 284.104858 NIST#: 458204 ID#: 243098 DB: mainlib
Contributor: NIH Small Molecule Repository/NIST Mass Spectrometry Data Center, 2012
InChIKey: DRZLMVKTTVZMGY-UHFFFAOYSA-N Non-stereo
10 largest peaks:

284	999	135	811	251	481	152	192	153	188	285	182	165	135	77	101	181	95	252	85

187 m/z Values and Intensities:

15	39	27	9	28	6	29	6	37	1	38	4	39	38	40	5	41	18	42	7
43	9	45	10	50	17	51	36	52	8	53	14	54	1	55	18	57	3	59	47
62	18	63	58	64	32	65	19	66	4	67	2	68	3	69	6	74	21	75	31
76	45	77	101	78	12	79	13	82	17	84	4	85	4	86	15	87	28	88	16
89	41	90	15	91	21	92	50	93	6	94	2	95	2	96	3	98	53	99	20
100	10	101	13	102	22	103	11	104	15	105	27	106	5	107	40	108	15	109	3
110	4	111	9	112	15	113	53	114	21	115	46	116	9	117	11	118	9	119	8
120	6	121	30	122	6	123	3	126	75	127	45	128	46	129	12	130	5	131	5

132 11	133 9	134 6	135 811	136 68	137 12	138 6	139 78	140 22	141 48
142 68	143 12	144 6	145 19	146 3	147 2	149 2	150 14	151 52	152 192
153 188	154 74	155 25	156 18	157 13	158 5	159 8	160 1	162 2	163 16
164 17	165 135	166 47	167 25	168 18	169 20	170 13	171 14	172 2	175 1
176 8	177 2	178 5	179 8	180 18	181 95	182 43	183 15	184 5	185 15
186 3	187 1	188 1	192 2	193 6	194 7	195 40	196 23	197 64	198 13
199 4	200 2	201 2	206 1	207 3	208 13	209 32	210 11	211 9	212 2
213 6	214 2	215 1	220 4	221 10	222 2	223 12	224 34	225 17	226 8
227 10	228 2	229 1	235 1	236 7	237 10	238 2	239 5	241 7	242 1
251 481	252 85	253 71	254 12	255 3	256 1	267 5	268 5	269 84	270 15
271 2	282 36	283 13	284 999	285 182	286 24	287 2			

Synonyms:
no synonyms.

Hit 4 : Androsta-1,4,6-trien-3-one, 17-hydroxy-, (17β)-
C19H24O2; MF: 603; RMF: 609; Prob 10.9%; CAS: 4075-12-1; Lib: mainlib; ID: 243096.

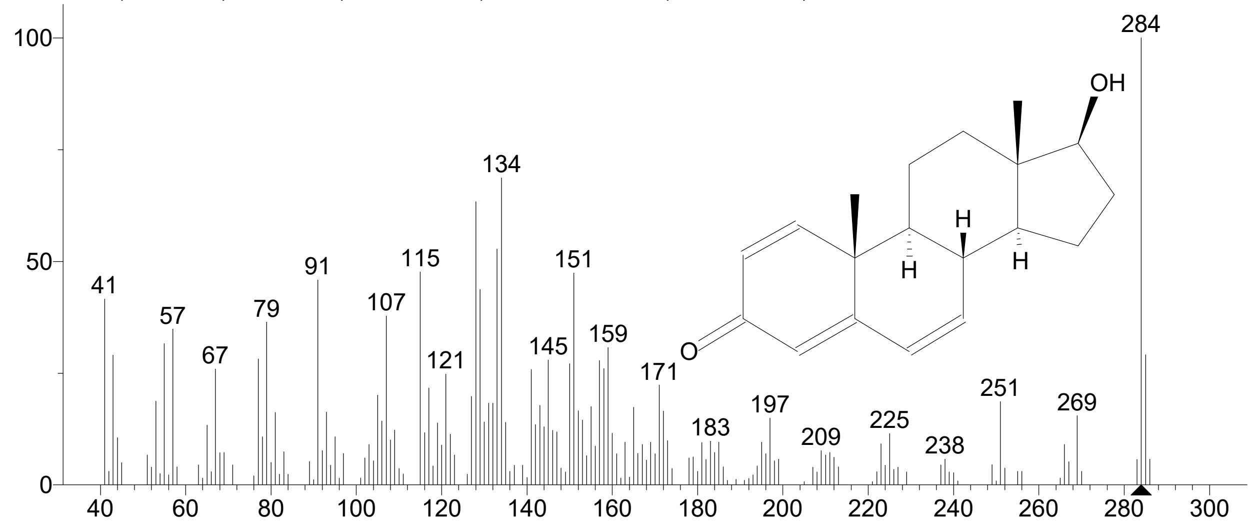

(mainlib) Androsta-1,4,6-trien-3-one, 17-hydroxy-, (17β)-

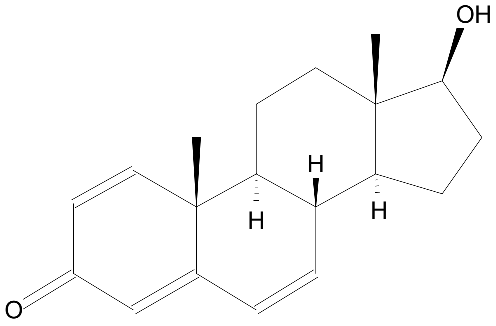

Name: Androsta-1,4,6-trien-3-one, 17-hydroxy-, (17β)-
Formula: $C_{19}H_{24}O_2$
MW: 284 Exact Mass: 284.17763 CAS#: 4075-12-1 NIST#: 126884 ID#: 243096 DB: mainlib
Other DBs: None
Contributor: LAC, NIDDK, NIH, Bethesda, MD 20892
InChIKey: WGFQJPRCLLMHRT-DYKIIFRCSA-N Non-stereo
10 largest peaks:

284 999	134 686	128 632	133 526	115 476	151 474	91 458	129 436	41 416	107 377

163 m/z Values and Intensities:

41 416	42 30	43 289	44 105	45 49	51 66	52 39	53 186	54 25	55 315
56 22	57 348	58 40	63 44	64 15	65 133	66 29	67 259	68 71	69 72
71 44	76 20	77 281	78 107	79 364	80 50	81 161	82 23	83 73	84 23
89 52	90 11	91 458	92 76	93 162	94 43	95 107	96 15	97 70	101 15
102 60	103 90	104 53	105 200	106 142	107 377	108 100	109 122	110 36	111 24
115 476	116 116	117 216	118 42	119 138	120 88	121 248	122 113	123 66	126 23
127 197	128 632	129 436	130 140	131 182	132 182	133 526	134 686	135 139	136 30

137 43	139 43	140 16	141 257	142 134	143 177	144 129	145 279	146 121	147 118
148 37	149 28	150 270	151 474	152 165	153 144	154 65	155 174	156 86	157 277
158 259	159 307	160 115	161 69	162 15	163 95	164 17	165 173	166 70	167 90
168 55	169 95	170 69	171 223	172 164	173 98	174 36	178 60	179 62	180 30
181 94	182 56	183 98	184 72	185 95	186 40	187 10	189 12	191 10	192 14
193 22	194 42	195 95	196 69	197 150	198 53	199 57	205 7	207 39	208 28
209 77	210 66	211 72	212 61	213 40	221 7	222 29	223 91	224 43	225 115
226 34	227 39	229 28	237 44	238 58	239 28	240 27	241 8	249 45	250 8
251 186	252 37	255 30	256 30	265 15	266 90	267 51	269 155	270 30	283 56
284 999	285 290	286 57							

Synonyms:
1.Androsta-1,4,6-trien-3-one, 17β-hydroxy-
2.1,4,6-Androstatrien-17β-ol-3-one
3.1,4,6-Androstatrien-3-one-17β-ol
4.17-Hydroxyandrosta-1,4,6-trien-3-one-, (17β)-
5.(17β)-17-Hydroxyandrosta-1,4,6-trien-3-one

Hit 5 : 4,9(11)-Androstadiene-3,17-dione
C19H24O2; MF: 596; RMF: 598; Prob 8.38%; Lib: mainlib; ID: 243233.

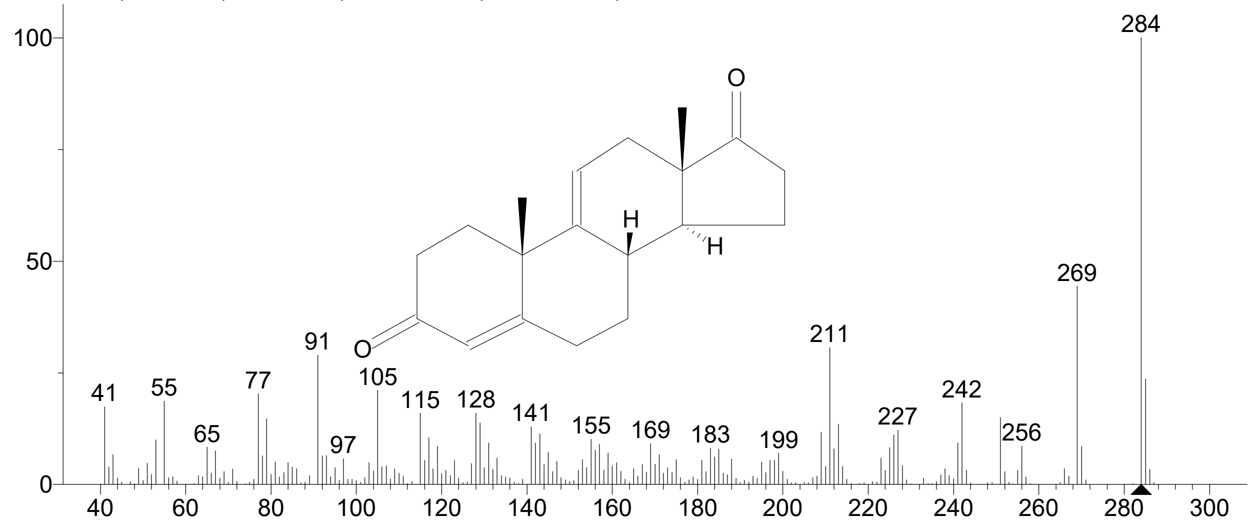

(mainlib) 4,9(11)-Androstadiene-3,17-dione

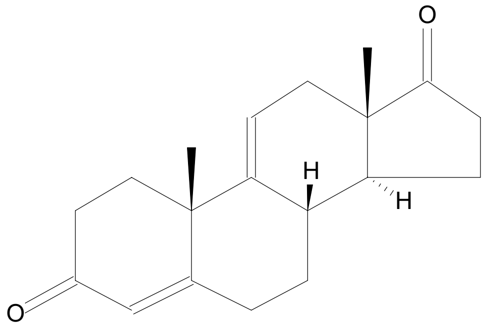

Name: 4,9(11)-Androstadiene-3,17-dione
Formula: $C_{19}H_{24}O_2$
MW: 284 Exact Mass: 284.17763 NIST#: 126893 ID#: 243233 DB: mainlib
Contributor: LAC, NIDDK, NIH, Bethesda, MD 20892
InChIKey: HJTUINCBGMVXOB-LNMJFAINSA-N Non-stereo
10 largest peaks:

284 999	269 443	211 306	91 289	285 235	105 211	77 203	55 186	242 183	41 174

211 m/z Values and Intensities:

41 174	42 38	43 66	44 14	45 5	47 6	48 1	49 36	50 8	51 47
52 22	53 99	55 186	56 15	57 17	58 7	62 2	63 19	64 17	65 83
66 25	67 75	68 13	69 28	70 4	71 34	72 6	74 1	75 5	76 11
77 203	78 63	79 146	80 22	81 50	82 16	83 26	84 48	85 38	86 35
87 3	88 5	89 19	91 289	92 63	93 64	94 17	95 37	96 9	97 58
98 11	99 11	100 8	101 4	102 15	103 48	104 30	105 211	106 39	107 41
108 12	109 34	110 25	111 18	112 2	113 6	115 159	116 53	117 104	118 35
119 85	120 24	121 31	122 20	123 54	124 14	125 3	126 5	127 46	128 160

129 137 | 130 37 | 131 92 | 132 33 | 133 58 | 134 19 | 135 16 | 136 14 | 137 5 | 138 2 |
139 11 | 141 130 | 142 92 | 143 113 | 144 44 | 145 71 | 146 28 | 147 51 | 148 15 | 149 10 |
150 6 | 151 8 | 152 31 | 153 55 | 154 37 | 155 102 | 156 76 | 157 89 | 158 32 | 159 70 |
160 41 | 161 48 | 162 29 | 163 11 | 164 4 | 165 35 | 166 18 | 167 44 | 168 27 | 169 92 |
170 45 | 171 66 | 172 25 | 173 37 | 174 27 | 175 55 | 176 15 | 177 5 | 178 10 | 179 16 |
180 11 | 181 53 | 182 28 | 183 81 | 184 61 | 185 78 | 186 25 | 187 21 | 188 56 | 189 13 |
190 3 | 191 9 | 192 5 | 193 18 | 194 13 | 195 50 | 196 26 | 197 53 | 198 53 | 199 70 |
200 29 | 201 11 | 202 3 | 203 3 | 205 4 | 206 3 | 207 15 | 208 18 | 209 116 | 210 40 |
211 306 | 212 79 | 213 134 | 214 40 | 215 11 | 216 2 | 218 2 | 219 3 | 221 6 | 222 5 |
223 58 | 224 31 | 225 82 | 226 110 | 227 122 | 228 42 | 229 10 | 230 Tr | 233 13 | 234 2 |
235 2 | 236 6 | 237 21 | 238 35 | 239 20 | 240 12 | 241 92 | 242 183 | 243 31 | 244 3 |
248 3 | 249 4 | 251 149 | 252 28 | 253 5 | 254 1 | 255 31 | 256 86 | 257 16 | 258 2 |
265 4 | 266 35 | 267 18 | 269 443 | 270 84 | 271 10 | 282 2 | 284 999 | 285 235 | 286 33 |
287 3 |

Synonyms:

1.Androst-4,9-dien-3,17-dione

Hit 6 : Allogibberic acid
C18H20O3; MF: 596; RMF: 596; Prob 8.38%; CAS: 427-79-2; Lib: mainlib; ID: 243120.

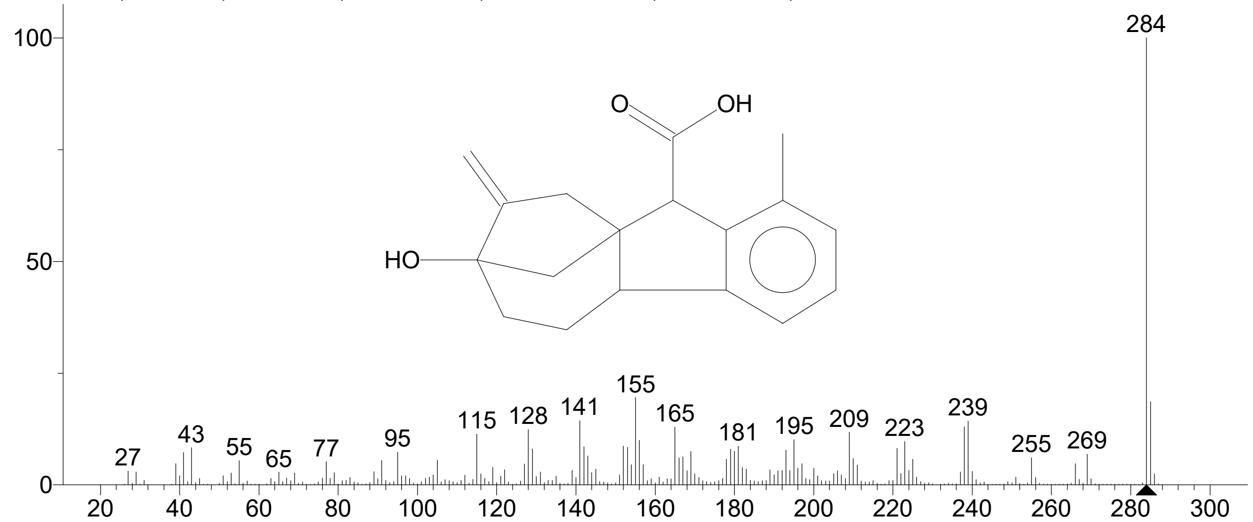

(mainlib) Allogibberic acid

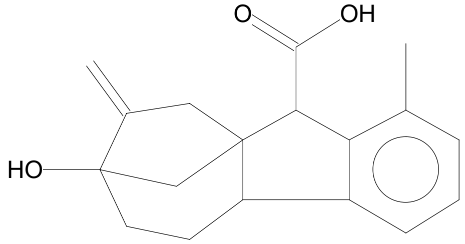

Name: Allogibberic acid
Formula: $C_{18}H_{20}O_3$
MW: 284 Exact Mass: 284.141245 CAS#: 427-79-2 NIST#: 151230 ID#: 243120 DB: mainlib
Other DBs: None
Contributor: Chemical Concepts
InChIKey: IFYWTLQMNWNCFH-UHFFFAOYSA-N Non-stereo
10 largest peaks:

284 999	155 195	285 185	141 144	239 143	165 130	238 129	128 124	209 118	115 114

252 m/z Values and Intensities:

26 1	27 31	28 3	29 28	30 1	31 10	38 1	39 47	40 20	41 72
42 7	43 83	44 5	45 14	46 Tr	47 1	50 3	51 20	52 7	53 26
54 2	55 55	56 3	57 8	58 1	59 Tr	60 1	62 2	63 14	64 7
65 28	66 7	67 15	68 9	69 26	70 3	71 6	72 1	73 Tr	74 2
75 6	76 15	77 52	78 14	79 27	80 2	81 9	82 10	83 16	84 6
85 4	86 1	87 2	88 5	89 29	90 13	91 54	92 10	93 5	94 7
95 73	96 20	97 20	98 14	99 4	100 2	101 7	102 14	103 17	104 22

105	55	106	6	107	11	108	9	109	7	110	5	111	10	112	21	113	3	114	12
115	114	116	24	117	14	118	7	119	38	120	5	121	19	122	33	123	6	124	1
125	2	126	8	127	46	128	124	129	80	130	18	131	28	132	5	133	10	134	10
135	19	136	3	137	2	138	3	139	32	140	16	141	144	142	85	143	64	144	27
145	35	146	7	147	6	148	4	149	2	150	4	151	22	152	86	153	83	154	45
155	195	156	99	157	45	158	9	159	13	160	4	161	17	162	6	163	13	164	13
165	130	166	60	167	62	168	31	169	74	170	24	171	16	172	9	173	7	174	5
175	6	176	9	177	14	178	57	179	79	180	75	181	87	182	38	183	35	184	10
185	8	186	7	187	9	188	9	189	33	190	22	191	31	192	32	193	77	194	32
195	102	196	37	197	47	198	14	199	11	200	37	201	20	202	9	203	8	204	8
205	25	206	31	207	22	208	14	209	118	210	58	211	44	212	8	213	6	214	6
215	9	216	3	217	1	218	2	219	9	220	10	221	81	222	25	223	97	224	32
225	57	226	17	227	7	228	3	229	4	230	2	232	1	233	2	234	3	235	2
236	2	237	28	238	129	239	143	240	30	241	11	242	4	243	6	244	1	245	Tr
246	Tr	247	Tr	248	2	249	7	250	4	251	17	252	3	253	1	254	4	255	61
256	16	257	3	258	Tr	259	Tr	260	Tr	261	Tr	262	Tr	263	1	264	2	265	3
266	47	267	12	268	3	269	69	270	13	271	2	272	Tr	273	Tr	274	Tr	275	Tr
276	Tr	277	Tr	278	Tr	279	1	280	Tr	281	Tr	282	1	283	4	284	999	285	185
286	24	287	2																

Synonyms:
1.7-Hydroxy-1-methyl-8-methylenegibba-1,3,4a(10a)-triene-10-carboxylic acid #

Hit 7 : Allogibberic acid
C18H20O3; MF: 587; RMF: 587; Prob 8.38%; CAS: 427-79-2; Lib: replib; ID: 37421.

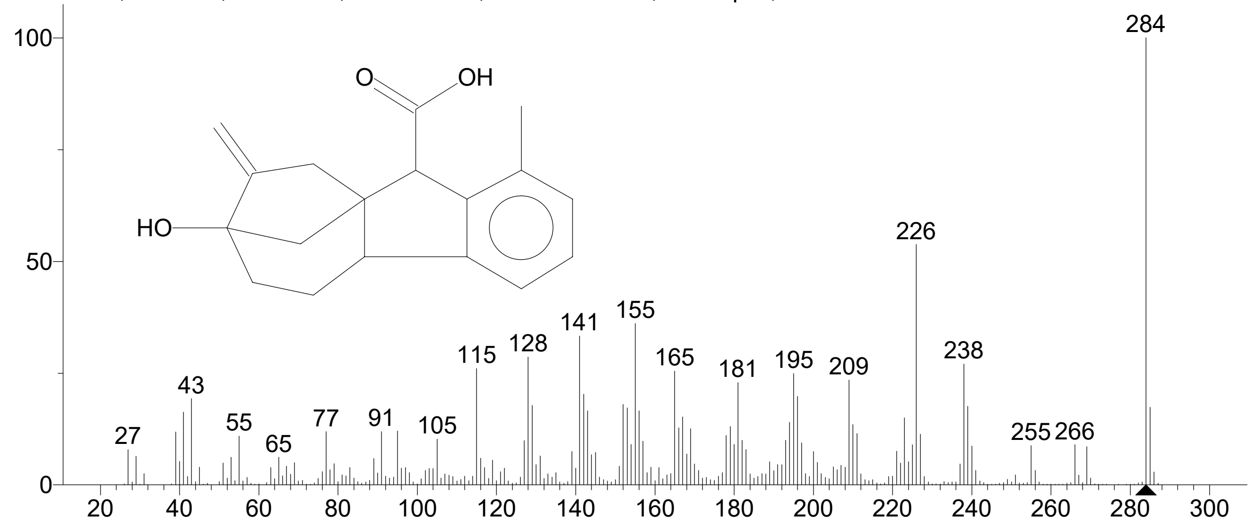

(replib) Allogibberic acid

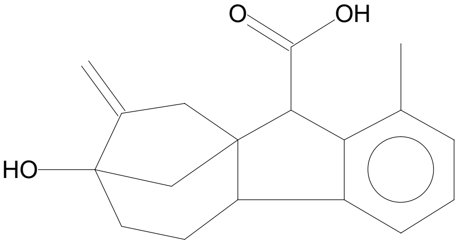

Name: Allogibberic acid
Formula: $C_{18}H_{20}O_3$
MW: 284 Exact Mass: 284.141245 CAS#: 427-79-2 NIST#: 151229 ID#: 37421 DB: replib
Other DBs: None
Contributor: Chemical Concepts
InChIKey: IFYWTLQMNWNCFH-UHFFFAOYSA-N Non-stereo
10 largest peaks:

284 999	226 537	155 361	141 333	128 286	238 270	115 260	165 255	195 249	209 235

250 m/z Values and Intensities:

26 2	27 79	28 6	29 63	30 1	31 25	38 2	39 118	40 52	41 162
42 18	43 193	44 7	45 39	46 1	47 3	50 7	51 48	52 15	53 61
54 9	55 110	56 8	57 16	58 3	59 1	60 2	62 5	63 38	64 13
65 62	66 20	67 41	68 23	69 49	70 8	71 10	72 1	73 1	74 4
75 14	76 29	77 119	78 33	79 47	80 7	81 22	82 20	83 38	84 15
85 7	86 3	87 6	88 10	89 58	90 26	91 120	92 19	93 15	94 17
95 120	96 37	97 38	98 27	99 6	100 2	101 13	102 32	103 36	104 36

105	103	106	15	107	24	108	21	109	18	110	8	111	12	112	19	113	9	114	19
115	260	116	59	117	38	118	14	119	55	120	9	121	29	122	37	123	8	124	3
125	4	126	17	127	98	128	286	129	177	130	45	131	64	132	14	133	24	134	17
135	27	136	6	137	3	138	7	139	74	140	37	141	333	142	202	143	165	144	67
145	72	146	17	147	11	148	8	149	6	150	11	151	41	152	179	153	171	154	90
155	361	156	165	157	97	158	27	159	39	160	9	161	38	162	13	163	22	164	25
165	255	166	127	167	151	168	68	169	125	170	46	171	32	172	15	173	16	174	11
175	10	176	19	177	27	178	110	179	130	180	90	181	229	182	99	183	78	184	24
185	15	186	18	187	25	188	24	189	51	190	31	191	45	192	45	193	99	194	139
195	249	196	197	197	93	198	25	199	18	200	74	201	50	202	25	203	16	204	13
205	40	206	33	207	43	208	39	209	235	210	134	211	114	212	24	213	11	214	9
215	11	216	4	217	2	218	4	219	18	220	19	221	75	222	48	223	149	224	51
225	89	226	537	227	113	228	18	229	6	230	2	231	2	232	2	233	7	234	5
235	6	236	6	237	46	238	270	239	175	240	86	241	32	242	8	243	5	244	1
245	Tr	246	1	247	3	248	5	249	12	250	6	251	22	252	4	253	3	254	5
255	88	256	32	257	6	258	1	259	1	260	Tr	261	Tr	262	1	263	1	264	3
265	5	266	90	267	21	268	5	269	85	270	15	271	2	272	1	273	1	274	1
277	Tr	279	Tr	280	1	281	1	282	4	283	6	284	999	285	173	286	28	287	3

Synonyms:
1.7-Hydroxy-1-methyl-8-methylenegibba-1,3,4a(10a)-triene-10-carboxylic acid #

Hit 8 : 5-[2-(2,3-Dihydro-1,4-benzodioxin-6-yl)ethenyl]-2-methoxyphenol
C17H16O4; MF: 583; RMF: 608; Prob 5.41%; CAS: 1257321-28-0; Lib: mainlib; ID: 243088.

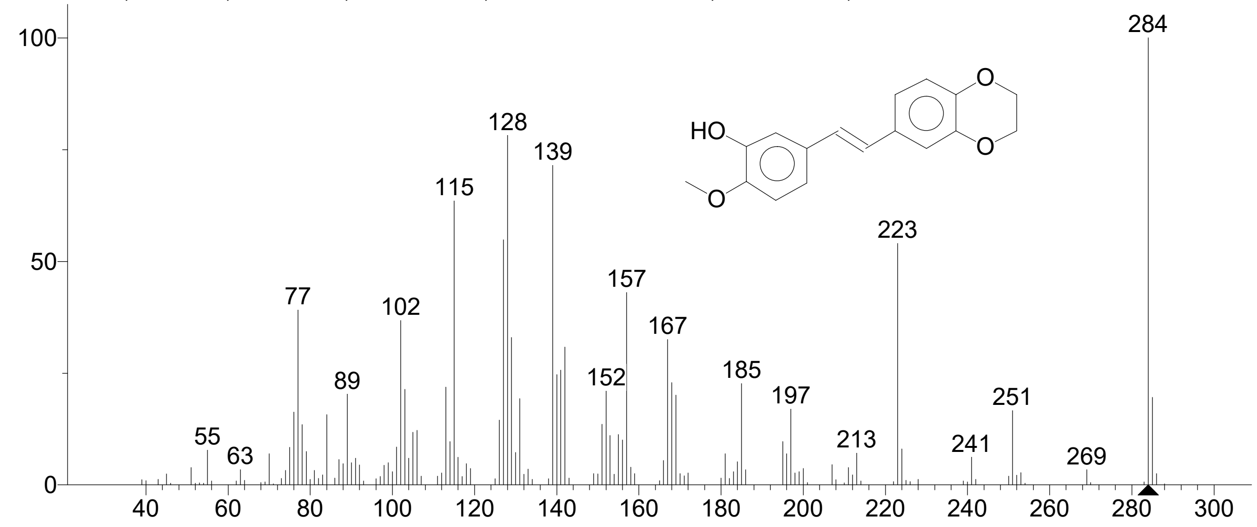

(mainlib) 5-[2-(2,3-Dihydro-1,4-benzodioxin-6-yl)ethenyl]-2-methoxyphenol

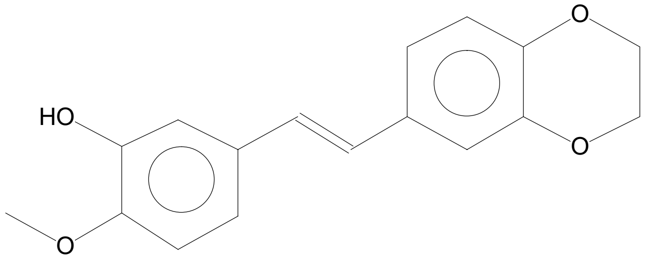

Name: 5-[2-(2,3-Dihydro-1,4-benzodioxin-6-yl)ethenyl]-2-methoxyphenol
Formula: $C_{17}H_{16}O_4$
MW: 284 Exact Mass: 284.104858 CAS#: 1257321-28-0 NIST#: 434814 ID#: 243088 DB: mainlib
Other DBs: None
Contributor: V.A.Korolev, Moscow, Russia
InChIKey: QTDSJRSHKSGNJC-NSCUHMNNSA-N Non-stereo
10 largest peaks:

284 999	128 781	139 714	115 635	127 547	223 540	157 430	77 391	102 367	129 329

137 m/z Values and Intensities:

39 11	40 9	43 12	45 24	46 3	51 38	52 3	53 4	54 3	55 78
56 8	62 8	63 35	64 10	68 5	69 6	70 69	71 2	73 14	74 32
75 83	76 162	77 391	78 134	79 74	80 12	81 32	82 14	83 22	84 156
86 15	87 56	88 47	89 203	90 49	91 59	92 44	93 8	96 13	97 18
98 43	99 49	100 29	101 84	102 367	103 213	104 59	105 117	106 121	107 19
111 19	112 26	113 218	114 96	115 635	116 61	117 18	118 47	119 36	125 13
126 144	127 547	128 781	129 329	130 72	131 192	132 23	133 35	134 12	138 13

139 714	140 246	141 256	142 307	143 15	149 25	150 24	151 135	152 210	153 110
154 23	155 112	156 100	157 430	158 39	159 25	165 9	166 54	167 325	168 228
169 200	170 25	171 20	172 26	180 15	181 69	182 14	183 29	184 51	185 227
186 33	195 96	196 69	197 170	198 26	199 29	200 36	201 5	207 45	208 11
210 4	211 38	212 22	213 71	214 8	222 7	223 540	224 80	225 10	226 7
228 12	239 8	240 6	241 62	242 12	250 19	251 166	252 21	253 27	254 3
269 34	270 5	283 6	284 999	285 195	286 25	288 2			

Synonyms:
no synonyms.

Hit 9 : 5,7,9(11)-Androstatriene, 3-hydroxy-17-oxo-
C19H24O2; MF: 575; RMF: 576; Prob 4.04%; Lib: mainlib; ID: 15194.

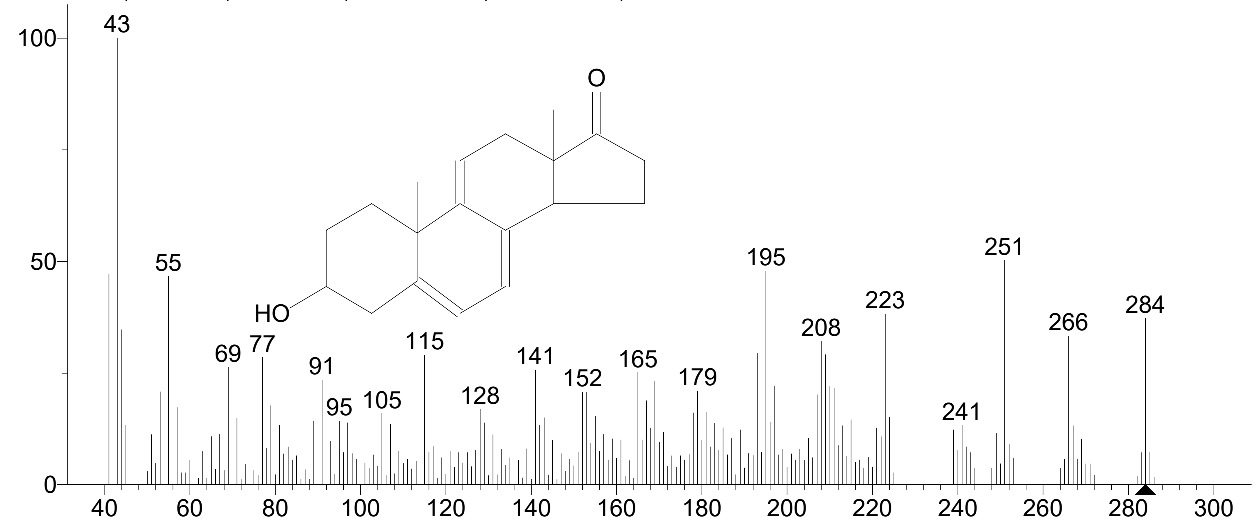

(mainlib) 5,7,9(11)-Androstatriene, 3-hydroxy-17-oxo-

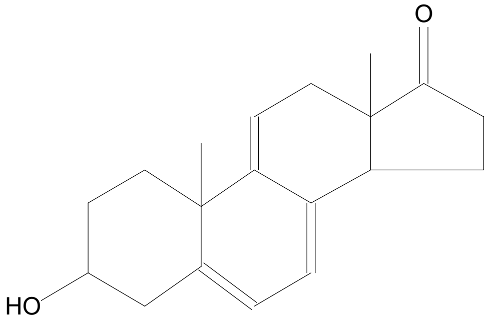

Name: 5,7,9(11)-Androstatriene, 3-hydroxy-17-oxo-
Formula: $C_{19}H_{24}O_2$
MW: 284 Exact Mass: 284.17763 NIST#: 251163 ID#: 15194 DB: mainlib
Contributor: LAC, NIDDK, NIH, Bethesda, MD 20892
InChIKey: BFFKDUOHNSMWGF-UHFFFAOYSA-N Non-stereo
10 largest peaks:

43 999	251 502	195 478	41 470	55 466	223 382	284 372	44 346	266 333	208 321

196 m/z Values and Intensities:

41 470	43 999	44 346	45 133	50 29	51 111	52 47	53 207	55 466	57 172
58 26	59 26	60 54	62 14	63 74	64 14	65 107	66 34	67 113	68 31
69 263	71 148	72 11	73 45	75 31	76 21	77 284	78 81	79 176	80 22
81 133	82 68	83 84	84 55	85 64	86 12	87 33	88 12	89 142	91 235
93 97	94 23	95 143	96 71	97 138	98 69	99 56	101 48	102 36	103 66
104 41	105 159	106 21	107 134	108 24	109 75	110 47	111 56	112 35	113 52
115 291	116 72	117 85	118 13	119 60	120 23	121 75	122 38	123 71	124 48
125 71	126 40	127 77	128 169	129 138	130 20	131 111	132 22	133 79	134 43

135 60 | 137 54 | 138 15 | 139 80 | 140 12 | 141 257 | 142 133 | 143 149 | 144 21 | 145 99 |
146 11 | 147 69 | 148 30 | 149 56 | 150 42 | 151 72 | 152 208 | 153 208 | 154 92 | 155 152 |
156 74 | 157 112 | 158 55 | 159 102 | 160 58 | 161 100 | 162 18 | 163 53 | 164 14 | 165 251 |
166 100 | 167 187 | 168 126 | 169 231 | 170 95 | 171 117 | 172 41 | 173 64 | 174 39 | 175 64 |
176 55 | 177 67 | 178 160 | 179 210 | 180 99 | 181 161 | 182 84 | 183 136 | 184 76 | 185 127 |
186 66 | 187 103 | 188 22 | 189 122 | 190 37 | 191 69 | 192 65 | 193 293 | 194 72 | 195 478 |
196 139 | 197 220 | 198 66 | 199 79 | 200 39 | 201 68 | 202 55 | 203 79 | 204 54 | 205 103 |
206 60 | 207 201 | 208 321 | 209 290 | 210 219 | 211 216 | 212 87 | 213 131 | 214 63 | 215 145 |
216 50 | 217 55 | 218 37 | 219 61 | 220 39 | 221 126 | 222 107 | 223 382 | 224 150 | 225 26 |
239 122 | 240 77 | 241 133 | 242 84 | 243 71 | 244 36 | 248 37 | 249 115 | 250 46 | 251 502 |
252 90 | 253 58 | 264 36 | 265 56 | 266 333 | 267 131 | 268 57 | 269 101 | 270 46 | 271 46 |
272 21 | 282 19 | 283 71 | 284 372 | 285 72 | 286 17 |
Synonyms:
1.3-Hydroxyandrosta-5,7,9(11)-trien-17-one #

Hit 10 : Gibberellin A73 methyl ester
C20H24O4; MF: 571; RMF: 652; Prob 3.41%; Lib: mainlib; ID: 218595.

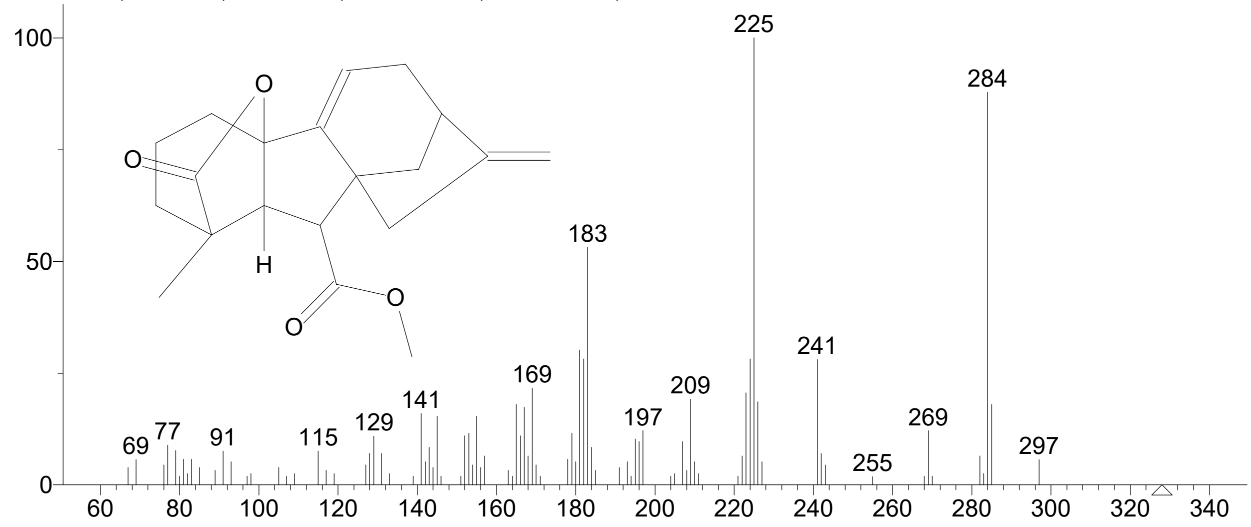

(mainlib) Gibberellin A73 methyl ester

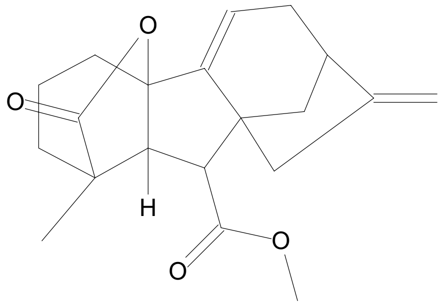

Name: Gibberellin A73 methyl ester
Formula: $C_{20}H_{24}O_4$
MW: 328 Exact Mass: 328.16746 NIST#: 440966 ID#: 218595 DB: mainlib
Contributor: The data for these pages was compiled and collated by Jake MacMillan, Paul Gaskin and Steve Croker. Link to the data base: http://www.plant-hormones.info/ga1info.htm
InChIKey: HQSDLFRIZKDOEE-UHFFFAOYSA-N Non-stereo
10 largest peaks:

225 999	284 877	183 531	181 301	182 281	224 281	241 281	169 217	223 205	209 192

89 m/z Values and Intensities:

67 38	69 57	76 44	77 89	79 76	80 19	81 57	82 25	83 57	85 38
89 32	91 76	93 51	97 19	98 25	105 38	107 19	109 25	115 76	117 32
119 25	127 44	128 70	129 109	131 70	133 25	139 19	141 160	142 51	143 83
144 38	145 153	146 19	151 19	152 109	153 115	154 44	155 153	156 38	157 64
163 32	164 19	165 179	166 109	167 173	168 64	169 217	170 44	171 19	178 57
179 115	180 51	181 301	182 281	183 531	184 83	185 32	191 38	193 51	194 19
195 102	196 96	197 121	204 19	205 25	207 96	208 32	209 192	210 51	211 25

221 19	222 64	223 205	224 281	225 999	226 185	227 51	241 281	242 70	243 44
255 19	268 19	269 121	270 19	282 64	283 25	284 877	285 179	297 57	

Synonyms:
no synonyms.
