# Supplementary material for: Uncovering a miltiradiene biosynthetic gene cluster in the Lamiaceae reveals a dynamic evolutionary trajectory
Source: Nat Commun. 2023 Jan 20;14:343. doi: 10.1038/s41467-023-35845-1 (PMC9860074; doi:10.1038/s41467-023-35845-1)
Supplement: Supplementary file 4 — Source Data [file 41467_2023_35845_MOESM4_ESM.zip › Supplementary Fig. 12, 20 Source Data.pdf]

Unknown; InLib=-133

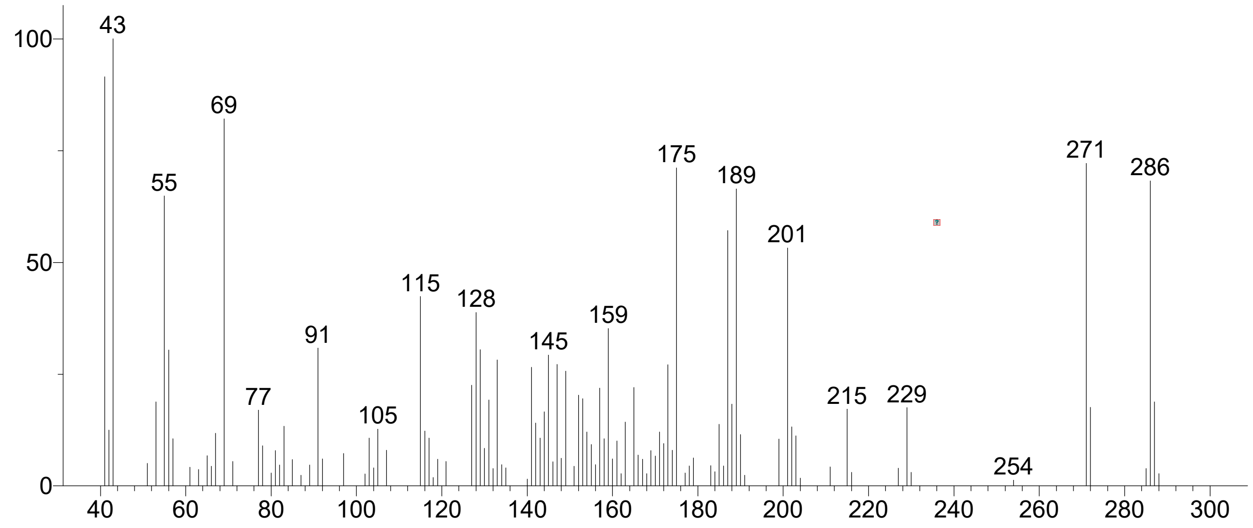

(Text File) Component at scan 2411 (14.278 min) [Model = +43u] in X:\ABBY GCMS\20211015\A_211015_024.D\DATA.MS

Name: Component at scan 2411 (14.278 min) [Model = +43u] in X:\ABBY GCMS\20211015\A_211015_024.D\DATA.
MS
MW: N/A ID#: 1017 DB: Text File
10 largest peaks:

43 999	41 913	69 820	271 721	175 711	286 681	189 664	55 648	187 570	201 532

111 m/z Values and Intensities:

41 913	42 124	43 999	51 50	53 187	55 648	56 303	57 105	61 41	63 36
65 67	66 43	67 117	69 820	71 54	77 169	78 89	80 28	81 78	82 46
83 133	85 58	87 23	89 46	91 308	92 60	97 72	102 26	103 106	104 40
105 127	107 79	115 423	116 122	117 106	118 18	119 59	121 54	127 224	128 388
129 304	130 83	131 191	132 38	133 281	134 47	135 40	140 15	141 264	142 140
143 106	144 165	145 293	146 53	147 271	148 61	149 256	151 43	152 202	153 194
154 120	155 92	156 47	157 218	158 105	159 352	160 60	161 100	162 27	163 142
165 219	166 68	167 59	168 27	169 78	170 66	171 120	172 94	173 270	174 79
175 711	177 28	178 44	179 62	183 45	184 31	185 137	186 44	187 570	188 182
189 664	190 114	191 23	199 104	201 532	202 131	203 111	204 17	211 42	215 171
216 30	227 39	229 175	230 30	254 13	271 721	272 175	285 38	286 681	287 187
288 27									

Synonyms:
no synonyms.

Hit 1 : Ferruginol
C20H30O; MF: 834; RMF: 839; Prob 57.7%; CAS: 514-62-5; Lib: mainlib; ID: 243935.

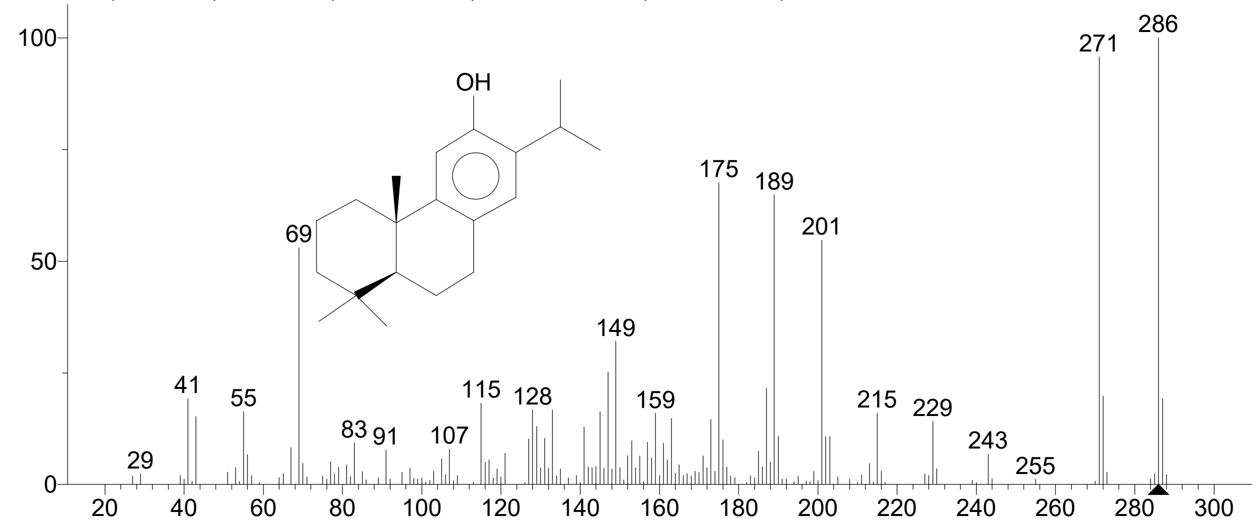

(mainlib) Ferruginol

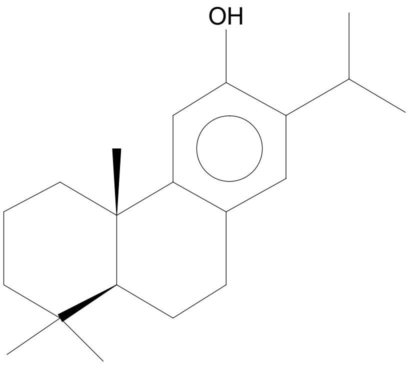

Name: Ferruginol
Formula: $C_{20}H_{30}O$
MW: 286 Exact Mass: 286.229666 CAS#: 514-62-5 NIST#: 413241 ID#: 243935 DB: mainlib
Other DBs: None
Contributor: NIST Mass Spectrometry Data Center
InChIKey: QXNWVJOHUAQHLM-AZUAARDMSA-N Non-stereo
10 largest peaks:

286 999	271 956	175 675	189 648	201 546	69 530	149 320	147 251	187 215	272 197

153 m/z Values and Intensities:

27 18	29 24	39 20	40 11	41 192	42 7	43 151	51 27	53 37	54 6
55 163	56 66	57 19	59 4	64 15	65 24	67 82	69 530	70 47	71 16
75 18	76 11	77 50	78 23	79 38	81 43	82 17	83 93	85 28	86 10
89 15	91 77	92 12	95 27	97 36	98 13	99 12	100 14	101 5	102 8
103 29	105 56	106 22	107 79	108 8	109 19	113 5	115 182	116 49	117 54
118 14	119 34	120 16	121 69	126 4	127 101	128 167	129 129	130 37	131 103
132 36	133 166	134 19	135 34	137 14	139 20	140 5	141 128	142 38	143 37

144 40	145 162	146 36	147 251	148 34	149 320	150 37	151 10	152 64	153 97
154 37	155 63	156 6	157 94	158 58	159 159	160 20	161 92	162 55	163 147
164 24	165 43	166 20	167 23	168 18	169 29	170 26	171 63	172 37	173 145
174 29	175 675	176 99	177 38	178 18	179 15	182 4	183 19	184 15	185 74
186 39	187 215	188 50	189 648	190 107	191 12	192 12	194 6	195 18	197 7
198 6	199 29	200 8	201 546	202 106	203 107	205 16	208 11	210 5	211 21
213 46	214 5	215 160	216 29	217 5	227 23	228 20	229 141	230 35	239 9
240 4	243 68	244 12	255 12	270 7	271 956	272 197	273 27	284 12	285 23
286 999	287 191	288 22							

Synonyms:
1.Abieta-9(11),8(14),12-trien-12-ol #
2.trans-Ferruginol
3.Podocarpa-8,11,13-trien-12-ol, 13-isopropyl-

Experimental RI median±deviation (#data)
Semi-standard non-polar:2325±0 (16)
Standard non-polar: 2283±N/A (1)
Polar: 2295±N/A (1)

Hit 2 : 2-Phenanthrenol, 4b,5,6,7,8,8a,9,10-octahydro-4b,8,8-trimethyl-1-(1-methylethyl)-, (4bS-trans)-
C20H30O; MF: 820; RMF: 828; Prob 36.1%; CAS: 511-15-9; Lib: mainlib; ID: 238936.

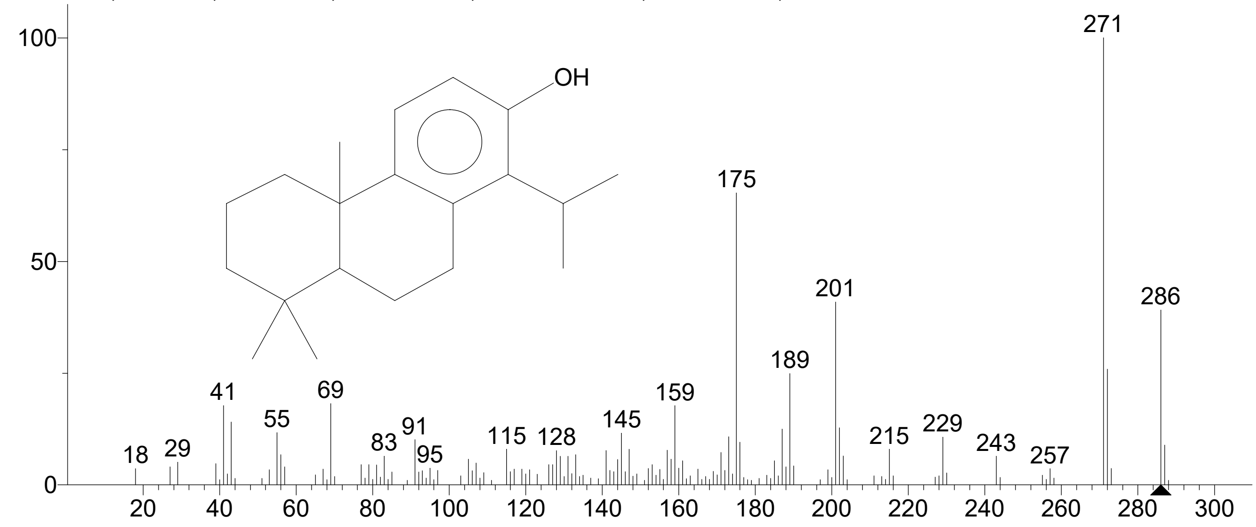

(mainlib) 2-Phenanthrenol, 4b,5,6,7,8,8a,9,10-octahydro-4b,8,8-trimethyl-1-(1-methylethyl)-, (4bS-trans)-

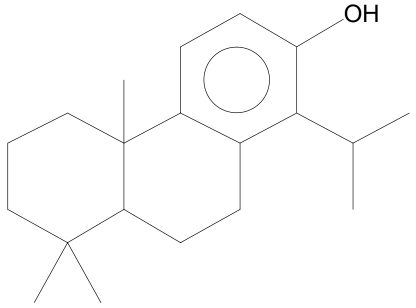

Name: 2-Phenanthrenol, 4b,5,6,7,8,8a,9,10-octahydro-4b,8,8-trimethyl-1-(1-methylethyl)-, (4bS-trans)-
Formula: $C_{20}H_{30}O$
MW: 286 Exact Mass: 286.229666 CAS#: 511-15-9 NIST#: 42592 ID#: 238936 DB: mainlib
Other DBs: NIH
Contributor: R RYHAGE MS-LAB KAROLINSKA INSTITUTET STOCKHOLM SWEDEN
InChIKey: ZRVDANDJSTYELM-UHFFFAOYSA-N Non-stereo
10 largest peaks:

271 999	175 653	201 409	286 391	272 258	189 249	69 182	41 178	159 177	43 140

136 m/z Values and Intensities:

18 37	27 40	29 51	39 47	40 11	41 178	42 24	43 140	44 14	51 14
53 33	55 117	56 67	57 40	65 22	67 35	68 11	69 182	70 18	77 45
78 15	79 45	80 12	81 44	82 17	83 65	84 12	85 28	89 10	91 101
92 28	93 31	94 15	95 38	96 11	97 32	103 20	105 57	106 31	107 48
108 15	109 27	111 10	115 80	116 29	117 35	119 35	120 24	121 33	123 23
126 45	127 45	128 77	129 63	130 18	131 63	132 25	133 67	134 18	135 21
137 15	139 13	141 76	142 32	143 29	144 56	145 116	146 29	147 79	148 19

149 24 | 151 10 | 152 36 | 153 45 | 154 21 | 155 34 | 156 12 | 157 77 | 158 57 | 159 177 |
160 37 | 161 53 | 162 13 | 163 20 | 165 35 | 166 12 | 167 18 | 168 12 | 169 30 | 170 22 |
171 72 | 172 32 | 173 107 | 174 24 | 175 653 | 176 95 | 177 16 | 178 11 | 179 10 | 181 14 |
183 21 | 184 14 | 185 53 | 186 20 | 187 124 | 188 40 | 189 249 | 190 42 | 197 11 | 199 33 |
200 16 | 201 409 | 202 127 | 203 64 | 204 11 | 211 20 | 213 18 | 214 12 | 215 80 | 216 20 |
227 17 | 228 20 | 229 107 | 230 26 | 243 64 | 244 16 | 255 21 | 256 12 | 257 36 | 258 15 |
271 999 | 272 258 | 273 36 | 286 391 | 287 88 | 288 10 |

Synonyms:
1.Podocarpa-8,11,13-trien-13-ol, 14-isopropyl-
2.Totarol
3.TOTAROL (B637832K176)
4.(+)-Totarol
5.14-Isopropylpodocarpa-8,11,13-trien-13-ol #
6.trans-Totarol

Experimental RI median±deviation (#data)
Semi-standard non-polar:2302±0 (14)
Standard non-polar: 2268±15 (7)

Hit 3 : 13-Isopropylpodocarpen-12-ol-20-al
C20H28O2; MF: 737; RMF: 788; Prob 3.53%; CAS: 24035-37-8; Lib: mainlib; ID: 238951.

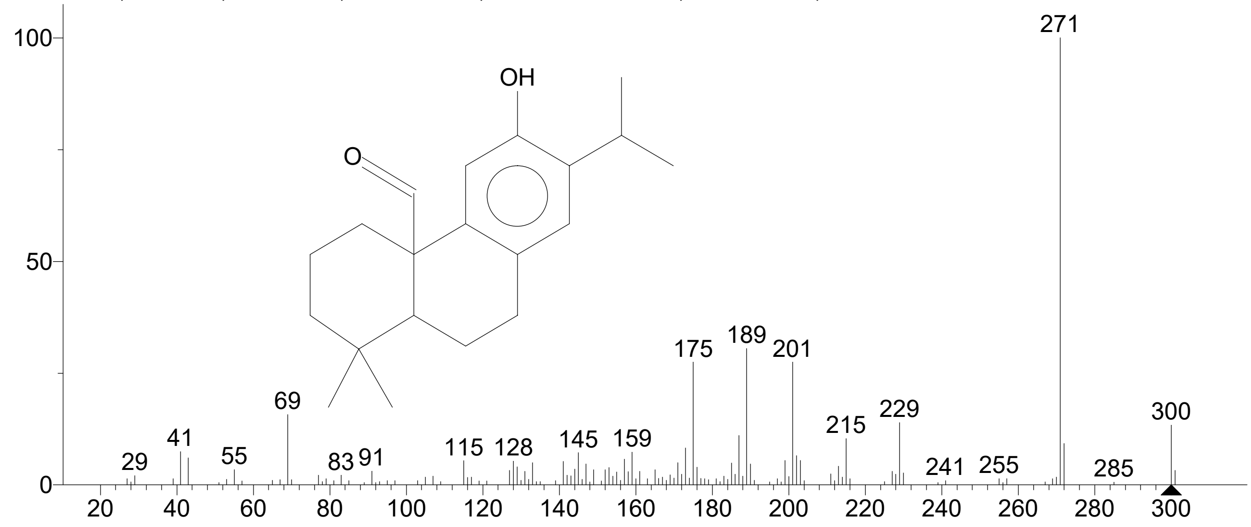

(mainlib) 13-Isopropylpodocarpen-12-ol-20-al

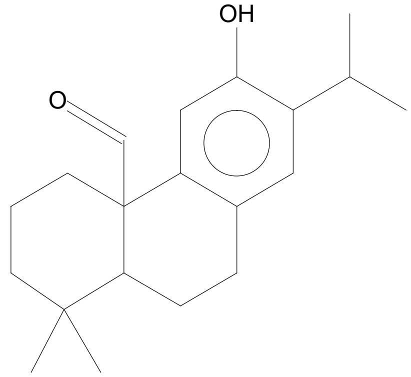

Name: 13-Isopropylpodocarpen-12-ol-20-al
Formula: $C_{20}H_{28}O_2$
MW: 300 Exact Mass: 300.208931 CAS#: 24035-37-8 NIST#: 42992 ID#: 238951 DB: mainlib
Other DBs: None
Contributor: R RYHAGE MS-LAB KAROLINSKA INSTITUTET STOCKHOLM SWEDEN
InChIKey: YPWYNONCSGZEQQ-UHFFFAOYSA-N Non-stereo
10 largest peaks:

271	999	189	305	175	274	201	274	69	158	229	140	300	134	187	110	215	104	272	92

125 m/z Values and Intensities:

27	13	28	6	29	21	39	13	41	75	43	60	51	5	53	12	55	35	57	8
65	10	67	11	69	158	70	11	77	21	78	7	79	13	81	9	83	22	85	9
89	5	91	31	92	5	93	7	95	9	97	9	103	9	105	17	107	19	109	7
115	54	116	16	117	17	119	8	121	8	127	32	128	53	129	40	130	10	131	30
132	12	133	49	134	7	135	7	139	9	141	52	142	21	143	20	144	35	145	72
146	12	147	46	148	6	149	33	151	8	152	33	153	38	154	19	155	28	156	10
157	57	158	29	159	74	160	13	161	30	163	13	165	33	166	14	167	17	168	10

169 22	170 15	171 49	172 23	173 82	174 15	175 274	176 39	177 14	178 13
179 11	181 13	182 7	183 19	184 12	185 48	186 23	187 110	188 19	189 305
190 46	191 10	195 6	197 13	198 6	199 54	200 18	201 274	202 65	203 54
204 9	211 24	212 9	213 41	214 17	215 104	216 13	225 7	227 30	228 23
229 140	230 26	239 5	241 10	255 15	256 5	257 14	267 6	269 13	270 17
271 999	272 92	285 7	300 134	301 32					

Synonyms:
1.12-Hydroxyabieta-9(11),8(14),12-trien-20-al #

Experimental RI median±deviation (#data)
Semi-standard non-polar:2569±N/A (1)
Standard non-polar: 2502±0 (2)

Hit 4 : 13-Isopropylpodocarpen-12-ol-20-al
C20H28O2; MF: 737; RMF: 788; Prob 3.53%; CAS: 24035-37-8; Lib: replib; ID: 37061.

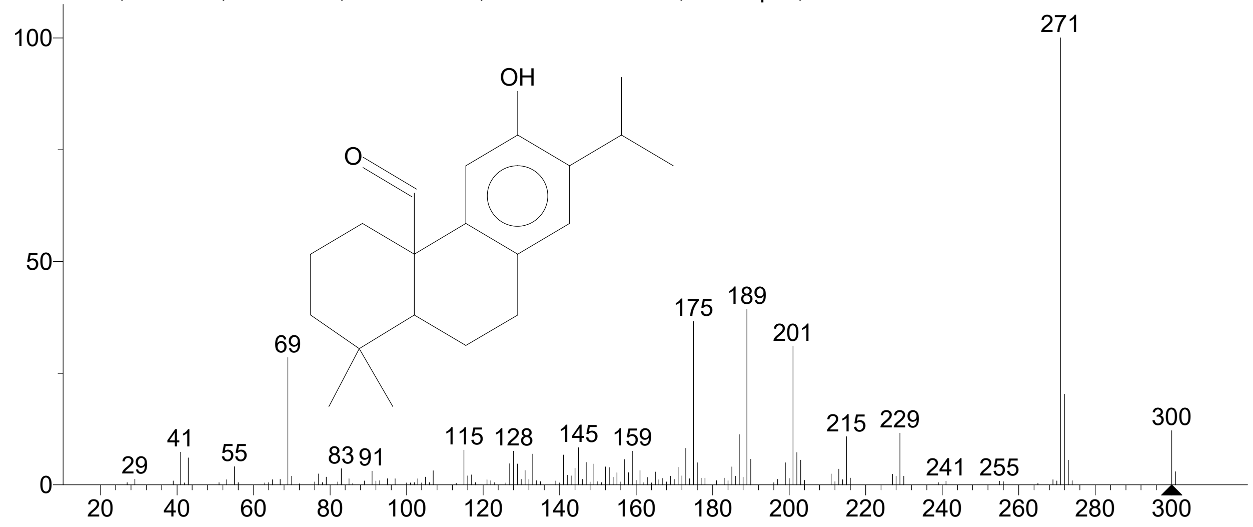

(replib) 13-Isopropylpodocarpen-12-ol-20-al

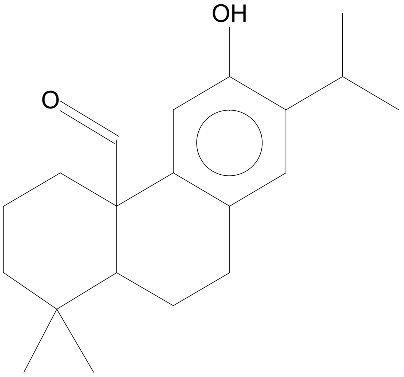

Name: 13-Isopropylpodocarpen-12-ol-20-al
Formula: $C_{20}H_{28}O_2$
MW: 300 Exact Mass: 300.208931 CAS#: 24035-37-8 NIST#: 414191 ID#: 37061 DB: replib
Other DBs: None
Contributor: NIST Mass Spectrometry Data Center
InChIKey: YPWYNONCSGZEQQ-UHFFFAOYSA-N Non-stereo
10 largest peaks:

271	999	189	392	175	365	201	310	69	284	272	202	300	122	229	116	187	112	215	108

137 m/z Values and Intensities:

27	5	29	13	39	8	41	73	42	4	43	60	51	5	53	11	55	41	56	5
63	4	64	5	65	11	67	12	69	284	70	19	72	2	76	6	77	24	78	5
79	17	82	6	83	36	85	13	86	3	89	8	91	31	92	8	93	9	95	13
97	13	100	4	101	4	102	5	103	13	104	4	105	17	106	5	107	31	113	3
115	78	116	20	117	22	118	5	121	11	122	9	123	5	126	4	127	47	128	76
129	46	130	12	131	32	132	12	133	68	134	9	135	7	139	8	140	4	141	66
142	21	143	20	144	37	145	84	146	12	147	50	148	6	149	46	150	7	151	5

152 40	153 38	154 17	155 27	156 6	157 56	158 27	159 76	160 10	161 32
162 5	163 16	164 4	165 28	166 11	167 14	168 6	169 19	170 12	171 39
172 20	173 81	174 13	175 365	176 49	177 15	178 15	181 9	183 15	184 9
185 40	186 19	187 112	188 17	189 392	190 57	196 5	197 12	199 49	200 14
201 310	202 72	203 55	204 10	211 24	212 7	213 35	214 11	215 108	216 15
227 23	228 19	229 116	230 19	239 5	241 9	255 9	256 7	265 3	269 11
270 8	271 999	272 202	273 55	274 9	300 122	301 29			

Synonyms:
1.12-Hydroxyabieta-9(11),8(14),12-trien-20-al #

Experimental RI median±deviation (#data)
Semi-standard non-polar:2569±N/A (1)
Standard non-polar: 2502±0 (2)

Hit 5 : Ferruginol
C20H30O; MF: 710; RMF: 904; Prob 57.7%; CAS: 514-62-5; Lib: replib; ID: 37524.

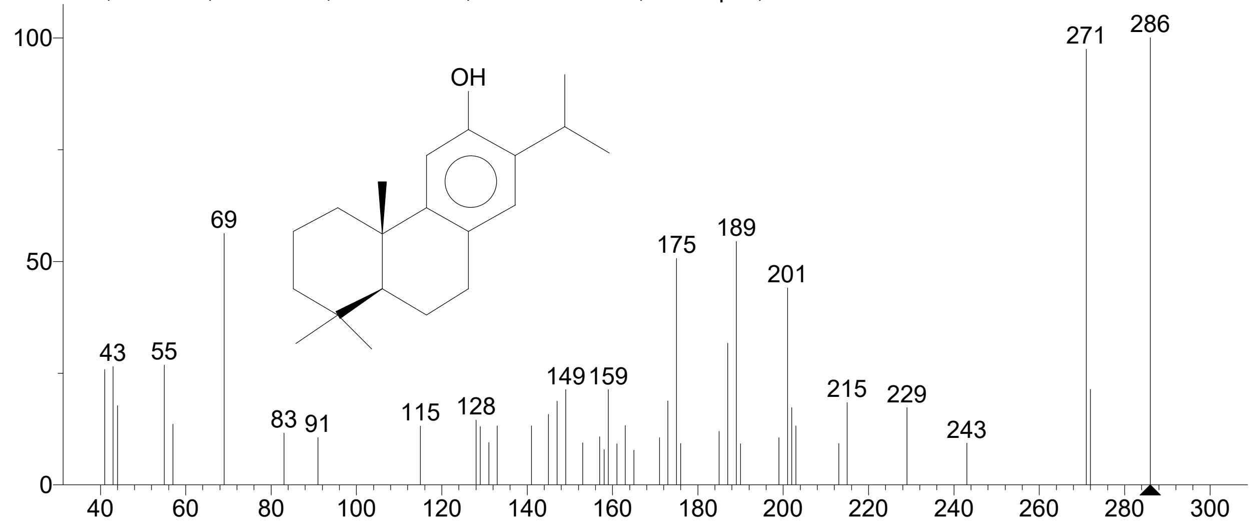

(replib) Ferruginol

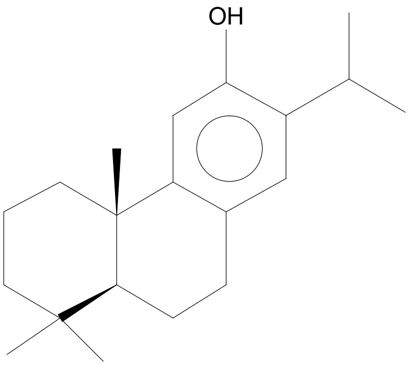

Name: Ferruginol
Formula: $C_{20}H_{30}O$
MW: 286 Exact Mass: 286.229666 CAS#: 514-62-5 NIST#: 148606 ID#: 37524 DB: replib
Other DBs: None
Contributor: ASES Database, Dalian Institute, P.R. China
InChIKey: QXNWVJOHUAQHLM-AZUAARDMSA-N Non-stereo
10 largest peaks:
286 999 | 271 973 | 69 562 | 189 544 | 175 506 | 201 440 | 187 316 | 55 268 | 43 265 | 41 257 |
43 m/z Values and Intensities:
41 257 | 43 265 | 44 176 | 55 268 | 57 135 | 69 562 | 83 116 | 91 106 | 115 132 | 128 145 |
129 130 | 131 94 | 133 131 | 141 131 | 145 157 | 147 186 | 149 213 | 153 93 | 157 107 | 158 78 |
159 213 | 161 91 | 163 132 | 165 77 | 171 105 | 173 187 | 175 506 | 176 92 | 185 119 | 187 316 |
189 544 | 190 91 | 199 105 | 201 440 | 202 172 | 203 131 | 213 92 | 215 184 | 229 173 | 243 93 |
271 973 | 272 213 | 286 999 |
Synonyms:
1.Abieta-9(11),8(14),12-trien-12-ol #

2.trans-Ferruginol
3.Podocarpa-8,11,13-trien-12-ol, 13-isopropyl-

Experimental RI median±deviation (#data)
Semi-standard non-polar:2325±0 (16)
Standard non-polar: 2283±N/A (1)
Polar: 2295±N/A (1)

Hit 6 : Pisiferol
C20H30O2; MF: 706; RMF: 767; Prob 0.96%; CAS: 24035-36-7; Lib: mainlib; ID: 238950.

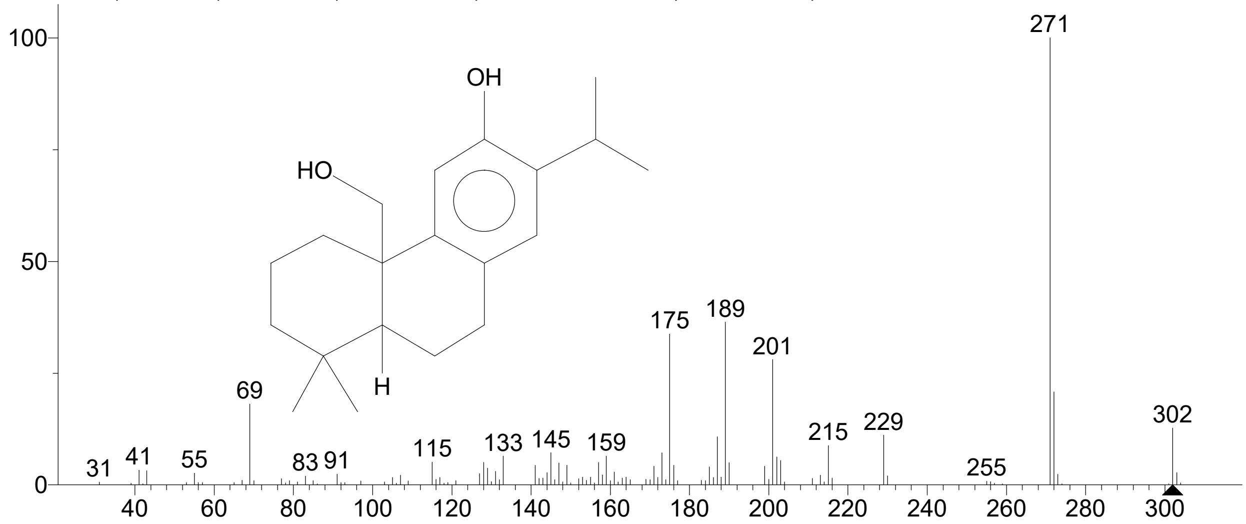

(mainlib) Pisiferol

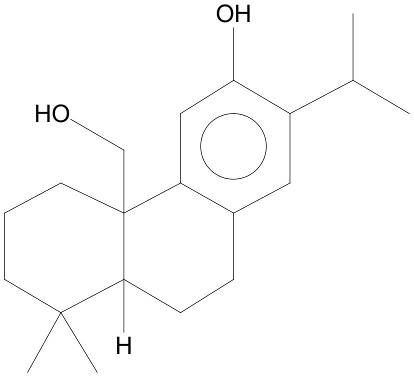

Name: Pisiferol
Formula: $C_{20}H_{30}O_2$
MW: 302 Exact Mass: 302.22458 CAS#: 24035-36-7 NIST#: 414875 ID#: 238950 DB: mainlib
Other DBs: None
Contributor: NIST Mass Spectrometry Data Center
InChIKey: NKGGFTFDYGTUSL-UHFFFAOYSA-N Non-stereo
10 largest peaks:

271 999	189 364	175 337	201 280	272 207	69 181	302 128	229 111	187 107	215 88

108 m/z Values and Intensities:

31 7	39 3	41 33	42 2	43 31	53 5	55 27	56 5	57 5	65 5
67 10	69 181	70 9	77 13	78 4	79 9	81 6	83 20	85 9	86 2
91 25	92 5	93 5	97 8	103 6	105 16	106 3	107 21	109 8	115 51
116 12	117 16	118 3	119 5	121 9	127 25	128 50	129 37	130 7	131 30
132 11	133 64	141 43	142 14	143 15	144 27	145 72	146 11	147 48	148 6
149 43	150 4	152 13	153 17	154 10	155 17	156 4	157 50	158 22	159 65
160 9	161 28	162 6	163 15	164 17	165 11	169 12	170 11	171 41	172 16

173 71	174 11	175 337	176 43	177 9	183 10	184 9	185 40	186 16	187 107
188 17	189 364	190 49	199 41	200 12	201 280	202 62	203 54	204 6	211 14
213 21	214 6	215 88	216 15	229 111	230 20	255 9	256 7	257 3	259 2
271 999	272 207	273 23	274 2	301 2	302 128	303 27	304 4		

Synonyms:
1.4a(2H)-Phenanthrenemethanol, 1,3,4,9,10,10a-hexahydro-6-hydroxy-1,1-dimethyl-7-(1-methylethyl)-, (4aR,10aS)-
2.4a(2H)-Phenanthrenemethanol, 1,3,4,9,10,10a-hexahydro-6-hydroxy-1,1-dimethyl-7-(1-methylethyl)-, (4aR-trans)-
3.Podocarpa-8,11,13-triene-12,17-diol, 13-isopropyl-
4.(+)-Pisiferol

Experimental RI median±deviation (#data)
Semi-standard non-polar:2615±N/A (1)

Hit 7 : 2-Phenanthrenol, 4b,5,6,7,8,8a,9,10-octahydro-4b,8,8-trimethyl-1-(1-methylethyl)-, acetate, (4bS-trans)-
C22H32O2; MF: 661; RMF: 678; Prob 0.20%; CAS: 15340-82-6; Lib: mainlib; ID: 239104.

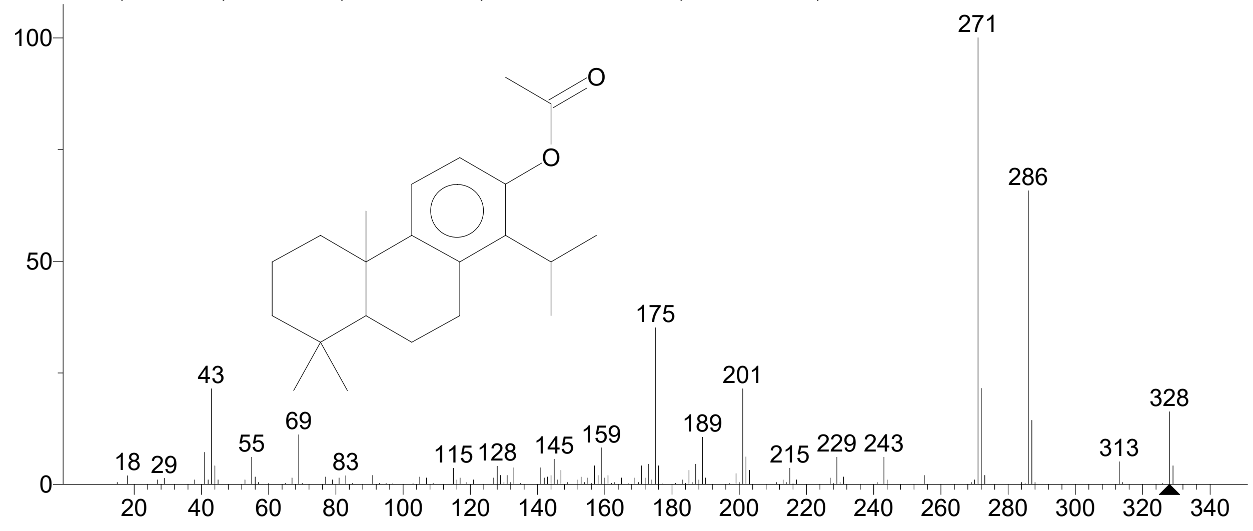

(mainlib) 2-Phenanthrenol, 4b,5,6,7,8,8a,9,10-octahydro-4b,8,8-trimethyl-1-(1-methylethyl)-, acetate, (4bS-trans)-

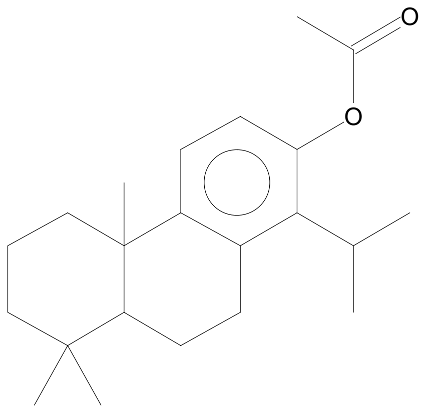

Name: 2-Phenanthrenol, 4b,5,6,7,8,8a,9,10-octahydro-4b,8,8-trimethyl-1-(1-methylethyl)-, acetate, (4bS-trans)-
Formula: $C_{22}H_{32}O_2$
MW: 328 Exact Mass: 328.24023 CAS#: 15340-82-6 NIST#: 15316 ID#: 239104 DB: mainlib
Other DBs: None
InChIKey: ORVBSFQTFRBNRP-UHFFFAOYSA-N Non-stereo
10 largest peaks:

271 999	286 657	175 351	43 214	201 214	272 214	328 163	287 143	69 112	189 106

121 m/z Values and Intensities:

15 4	18 20	27 10	29 14	38 10	41 71	42 10	43 214	44 41	45 10
53 10	55 61	56 16	57 4	60 2	65 2	67 14	69 112	70 2	77 16
79 10	81 14	83 20	85 2	91 20	93 2	95 2	97 2	103 2	105 16
107 14	109 2	115 37	116 10	117 14	119 4	121 10	127 14	128 41	129 20
130 4	131 20	132 4	133 37	135 2	141 37	142 14	143 16	144 20	145 57
146 10	147 31	149 4	152 10	153 16	154 4	155 14	156 2	157 41	158 20
159 82	160 14	161 20	162 2	163 4	165 14	167 2	169 14	170 4	171 41
172 14	173 45	174 10	175 351	176 41	177 2	181 2	183 10	184 2	185 31

186 4 | 187 45 | 188 10 | 189 106 | 190 14 | 197 2 | 199 24 | 200 4 | 201 214 | 202 61 |
203 31 | 211 4 | 213 10 | 214 4 | 215 37 | 216 2 | 217 10 | 227 14 | 228 2 | 229 61 |
230 4 | 231 16 | 241 4 | 243 61 | 244 10 | 255 20 | 269 4 | 270 10 | 271 999 | 272 214 |
273 20 | 284 4 | 285 2 | 286 657 | 287 143 | 288 4 | 313 51 | 314 4 | 326 2 | 328 163 |
329 41 |

Synonyms:
1.Podocarpa-8,11,13-trien-13-ol, 14-isopropyl-, acetate
2.Totaryl acetate
3.14-Isopropylpodocarpa-8,11,13-trien-13-yl acetate #
4.Totarol acetate
5.2-Phenanthrenol, 4b,5,6,7,8,8a,9,10-octahydro-4b,8,8-trimethyl-1-(1-methylethyl)-, 2-acetate, (4bS,8aS)-
6.2-Phenanthrenol, 4b,5,6,7,8,8a,9,10-octahydro-4b,8,8-trimethyl-1-(1-methylethyl)-, acetate, (4bS,8aS)-

Experimental RI median±deviation (#data)
Semi-standard non-polar:2417±0 (2)
Standard non-polar: 2437±0 (2)

Hit 8 : Podocarp-7-en-3-one, 13β-methyl-13-vinyl-
C20H30O; MF: 661; RMF: 668; Prob 0.20%; CAS: 7715-48-2; Lib: mainlib; ID: 243782.

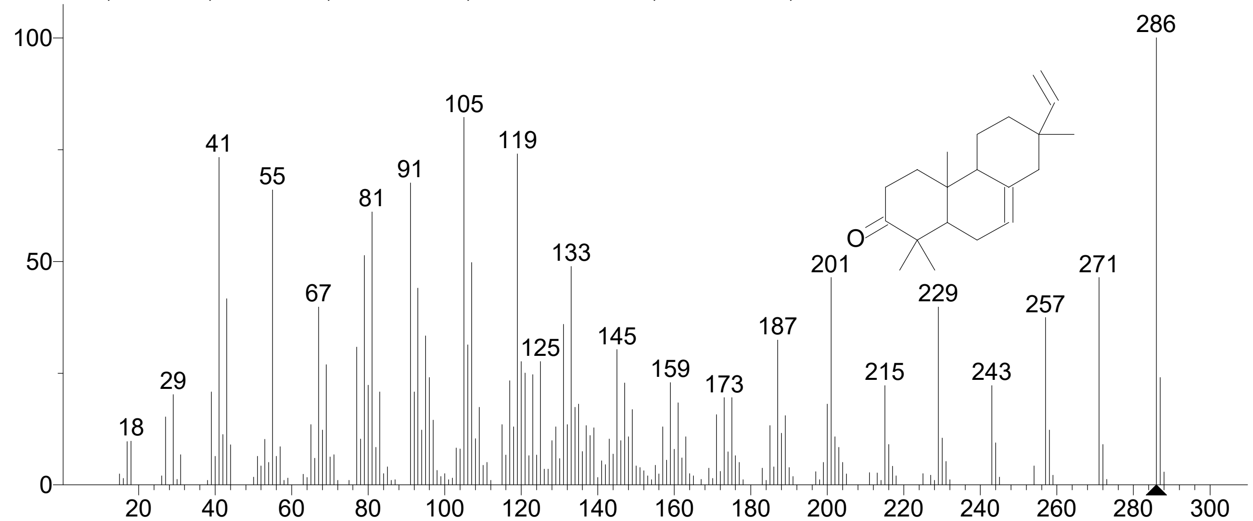

(mainlib) Podocarp-7-en-3-one, 13β-methyl-13-vinyl-

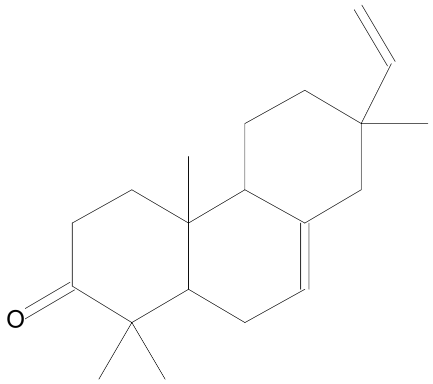

Name: Podocarp-7-en-3-one, 13β-methyl-13-vinyl-
Formula: $C_{20}H_{30}O$
MW: 286 Exact Mass: 286.229666 CAS#: 7715-48-2 NIST#: 42588 ID#: 243782 DB: mainlib
Other DBs: None
Contributor: R RYHAGE MS-LAB KAROLINSKA INSTITUTET STOCKHOLM SWEDEN
InChIKey: YAXFLCDQLAZOPS-UHFFFAOYSA-N Non-stereo
10 largest peaks:

286 999	105 821	119 740	41 732	91 675	55 659	81 610	79 512	107 496	133 488

177 m/z Values and Intensities:

15 24	16 14	17 96	18 98	26 20	27 151	29 202	30 12	31 67	38 10
39 207	40 63	41 732	42 112	43 415	44 89	50 17	51 63	52 42	53 101
54 50	55 659	56 63	57 85	58 10	59 15	63 23	64 16	65 134	66 59
67 398	68 122	69 268	70 62	71 67	72 10	75 10	77 307	78 102	79 512
80 222	81 610	82 83	83 207	84 25	85 40	86 10	87 11	91 675	92 207
93 439	94 122	95 332	96 239	97 144	98 32	99 18	100 25	101 11	102 15
103 82	104 80	105 821	106 312	107 496	108 103	109 173	110 43	111 50	112 10

115 134	116 66	117 232	118 129	119 740	120 276	121 249	122 65	123 246	124 66
125 276	126 35	127 35	128 98	129 129	130 58	131 358	132 134	133 488	134 173
135 180	136 74	137 132	138 110	139 127	140 16	141 53	142 45	143 102	144 68
145 302	146 98	147 227	148 107	149 168	150 42	151 38	152 31	153 20	154 11
155 43	156 24	157 129	158 55	159 229	160 79	161 183	162 60	163 107	164 25
165 20	167 12	169 37	170 14	171 156	172 30	173 195	174 73	175 195	176 65
177 50	178 11	183 37	184 10	185 132	186 40	187 324	188 115	189 154	190 38
191 18	197 29	198 11	199 50	200 180	201 463	202 107	203 83	204 50	205 24
211 27	213 26	214 10	215 222	216 90	217 41	218 20	225 25	227 21	228 10
229 398	230 104	231 52	232 11	243 222	244 93	245 17	254 42	257 374	258 122
259 21	271 463	272 90	273 12	286 999	287 239	288 28			

Synonyms:
1.Pimara-7,15-dien-3-one #

Experimental RI median±deviation (#data)
Semi-standard non-polar:2257±22 (3)

Hit 9 : Podocarp-7-en-3-one, 13β-methyl-13-vinyl-
C20H30O; MF: 651; RMF: 653; Prob 0.20%; CAS: 7715-48-2; Lib: replib; ID: 37498.

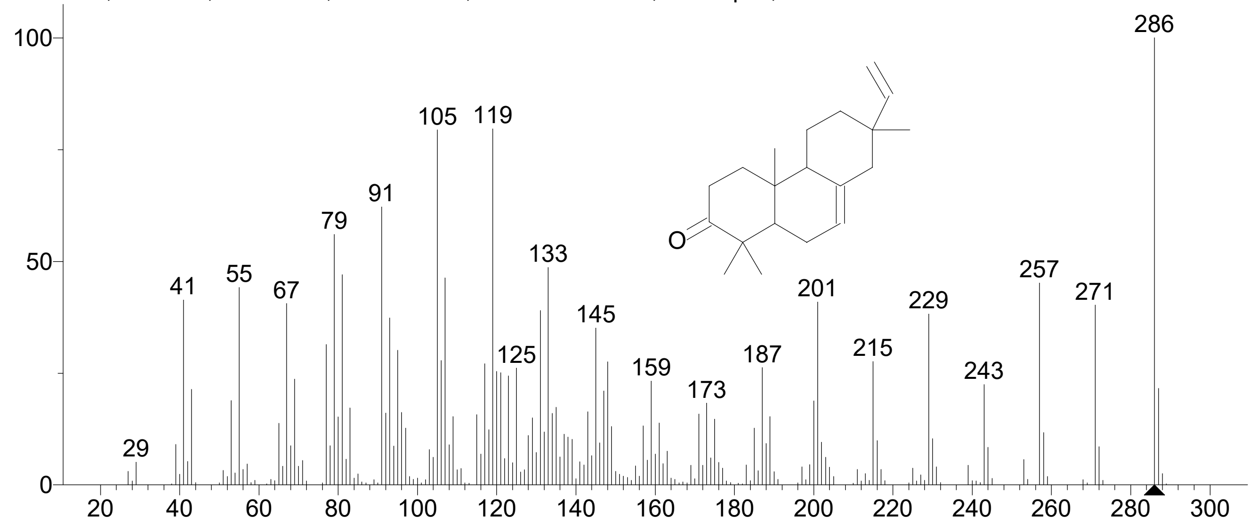

(replib) Podocarp-7-en-3-one, 13β-methyl-13-vinyl-

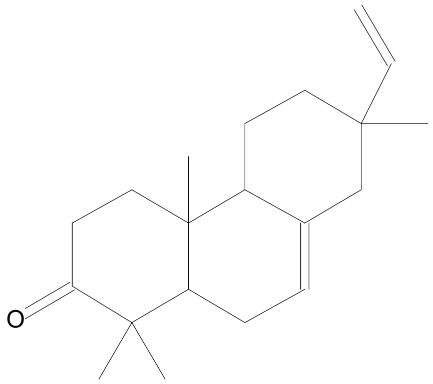

Name: Podocarp-7-en-3-one, 13β-methyl-13-vinyl-
Formula: $C_{20}H_{30}O$
MW: 286 Exact Mass: 286.229666 CAS#: 7715-48-2 NIST#: 466212 ID#: 37498 DB: replib
Other DBs: None
Contributor: NIST Mass Spectrometry Data Center
InChIKey: YAXFLCDQLAZOPS-UHFFFAOYSA-N Non-stereo
10 largest peaks:

286 999	119 796	105 793	91 621	79 560	133 486	81 469	107 462	257 452	55 442

193 m/z Values and Intensities:

27 30	28 8	29 51	38 2	39 90	40 23	41 413	42 52	43 213	44 5
50 4	51 32	52 18	53 188	54 26	55 442	56 34	57 46	58 5	59 10
62 2	63 12	64 9	65 137	66 41	67 405	68 87	69 236	70 41	71 54
72 8	76 4	77 313	78 87	79 560	80 151	81 469	82 57	83 171	84 15
85 24	86 6	87 4	89 11	90 4	91 621	92 160	93 372	94 86	95 300
96 161	97 126	98 18	99 12	100 15	101 4	102 11	103 78	104 61	105 793
106 277	107 462	108 89	109 152	110 33	111 36	112 4	113 3	115 156	116 68

117 270	118 123	119 796	120 253	121 250	122 58	123 243	124 49	125 261	126 28
127 33	128 110	129 149	130 72	131 389	132 118	133 486	134 159	135 173	136 62
137 113	138 106	139 101	140 13	141 51	142 44	143 163	144 65	145 351	146 93
147 209	148 274	149 130	150 30	151 23	152 19	153 16	154 10	155 42	156 19
157 131	158 55	159 232	160 68	161 138	162 47	163 75	164 15	165 12	166 4
167 6	168 4	169 43	170 13	171 158	172 43	173 183	174 60	175 146	176 50
177 37	178 8	179 5	181 3	182 2	183 44	184 9	185 126	186 31	187 262
188 92	189 152	190 29	191 12	196 4	197 40	198 11	199 45	200 187	201 409
202 95	203 61	204 39	205 18	210 3	211 34	212 8	213 25	214 10	215 276
216 98	217 34	218 9	224 4	225 37	226 8	227 22	228 10	229 382	230 103
231 40	232 5	239 43	240 9	241 8	242 5	243 224	244 83	245 14	253 56
254 12	257 452	258 116	259 18	268 11	269 4	271 402	272 85	273 10	286 999
287 215	288 25	289 2							

Synonyms:

1.Pimara-7,15-dien-3-one #

Experimental RI median±deviation (#data)

Semi-standard non-polar:2257±22 (3)

Hit 10 : 5-Androstene, 4,4-dimethyl-
C21H34; MF: 650; RMF: 651; Prob 0.14%; Lib: mainlib; ID: 239102.

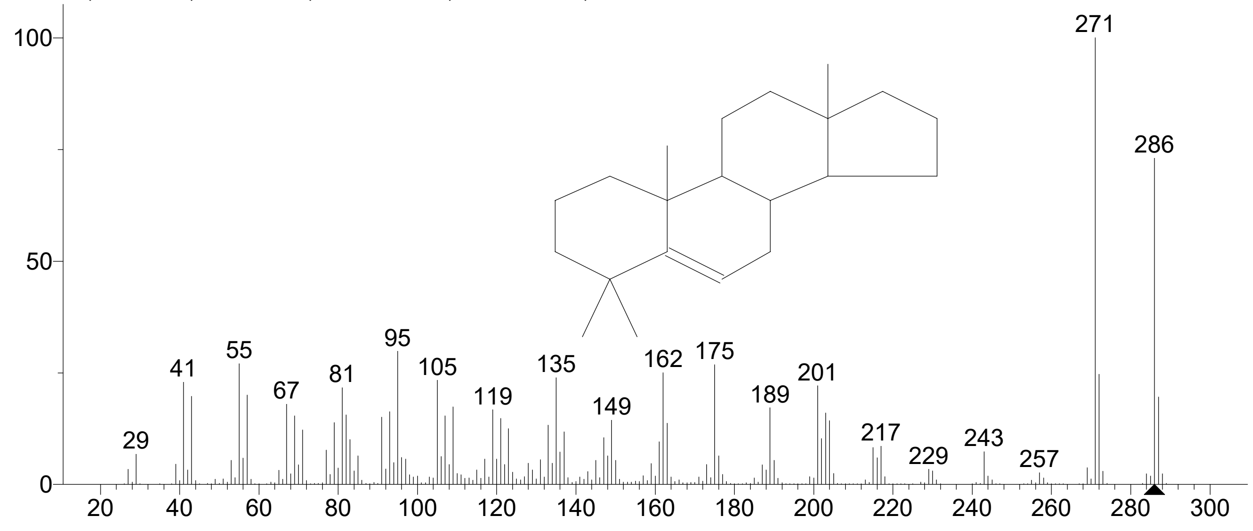

(mainlib) 5-Androstene, 4,4-dimethyl-

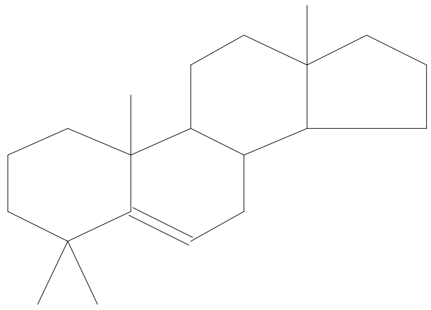

Name: 5-Androstene, 4,4-dimethyl-
Formula: $C_{21}H_{34}$
MW: 286 Exact Mass: 286.26605 NIST#: 194154 ID#: 239102 DB: mainlib
Contributor: Chemical Concepts
InChIKey: RAYCKELWUDCADQ-UHFFFAOYSA-N   Non-stereo
10 largest peaks:

271 999	286 730	95 298	55 271	175 268	162 250	272 246	135 239	105 233	41 229

231 m/z Values and Intensities:

26 1	27 33	28 5	29 68	30 1	35 1	38 1	39 45	40 8	41 229
42 32	43 196	44 8	45 1	47 2	48 1	49 11	50 2	51 12	52 5
53 53	54 15	55 271	56 58	57 199	58 11	59 1	60 Tr	62 Tr	63 5
64 3	65 31	66 11	67 180	68 23	69 153	70 43	71 121	72 8	73 1
74 2	75 2	76 3	77 76	78 22	79 138	80 36	81 217	82 155	83 100
84 30	85 63	86 9	87 2	88 1	89 4	90 2	91 150	92 34	93 162
94 48	95 298	96 60	97 56	98 21	99 16	100 18	101 3	102 3	103 16
104 14	105 233	106 62	107 153	108 44	109 173	110 24	111 21	112 13	113 14

114 9 | 115 32 | 116 13 | 117 56 | 118 16 | 119 167 | 120 56 | 121 147 | 122 44 | 123 124 |
124 27 | 125 12 | 126 10 | 127 17 | 128 47 | 129 32 | 130 12 | 131 55 | 132 16 | 133 132 |
134 47 | 135 239 | 136 72 | 137 117 | 138 15 | 139 4 | 140 6 | 141 16 | 142 11 | 143 28 |
144 10 | 145 53 | 146 15 | 147 104 | 148 63 | 149 144 | 150 53 | 151 11 | 152 5 | 153 5 |
154 5 | 155 7 | 156 6 | 157 19 | 158 8 | 159 46 | 160 18 | 161 95 | 162 250 | 163 136 |
164 16 | 165 6 | 166 10 | 167 3 | 168 3 | 169 4 | 170 4 | 171 16 | 172 7 | 173 44 |
174 15 | 175 268 | 176 63 | 177 22 | 178 6 | 179 2 | 180 1 | 181 1 | 182 1 | 183 3 |
184 2 | 185 14 | 186 5 | 187 43 | 188 32 | 189 171 | 190 53 | 191 13 | 192 3 | 193 Tr |
194 Tr | 195 1 | 196 Tr | 197 1 | 198 1 | 199 17 | 200 14 | 201 221 | 202 102 | 203 159 |
204 142 | 205 24 | 206 2 | 207 Tr | 208 Tr | 209 Tr | 210 Tr | 211 Tr | 212 Tr | 213 10 |
214 4 | 215 82 | 216 59 | 217 86 | 218 17 | 219 1 | 220 Tr | 221 Tr | 222 Tr | 223 Tr |
225 Tr | 227 5 | 228 3 | 229 34 | 230 30 | 231 10 | 232 1 | 240 Tr | 241 4 | 242 2 |
243 73 | 244 18 | 245 10 | 246 1 | 247 Tr | 253 Tr | 254 Tr | 255 9 | 256 3 | 257 27 |
258 14 | 259 3 | 260 Tr | 261 Tr | 262 Tr | 263 Tr | 267 1 | 268 1 | 269 37 | 270 12 |
271 999 | 272 246 | 273 29 | 274 2 | 283 2 | 284 23 | 285 18 | 286 730 | 287 195 | 288 23 |
289 2 |

Synonyms:
1.4,4,10,13-Tetramethyl-2,3,4,7,8,9,10,11,12,13,14,15,16,17-tetradecahydro-1H-cyclopenta[a]phenanthrene
2.4,4-Dimethylandrost-5-ene #

Experimental RI median±deviation (#data)
Semi-standard non-polar:2219±N/A (1)
